# Ancient genomes reveal an extensive kinship network and endogamy in a Three-Kingdoms period society in Korea

**DOI:** 10.1126/sciadv.ady8614

**Published:** 2026-04-08

**Authors:** Hyoungmin Moon, Daewook Kim, Alina N. Hiss, Don-Nyeong Lee, Juhyeon Lee, Eirini Skourtanioti, Guido Alberto Gnecchi-Ruscone, Johannes Krause, Eun Jin Woo, Choongwon Jeong

**Affiliations:** ^1^ School of Biological Sciences, Seoul National University, Seoul 08826, Republic of Korea.; ^2^ Institute for Data Innovation in Science, Seoul National University, Seoul 08826, Republic of Korea.; ^3^ Museum of Yeungnam University, Yeungnam University, Gyeongsan 38541, Republic of Korea.; ^4^ Department of Archaeogenetics, Max Planck Institute for Evolutionary Anthropology, 04103 Leipzig, Germany.; ^5^ Department of History, Sejong University, Seoul 05006, Republic of Korea.

## Abstract

The burial complex of the Imdang-Joyeong site at Gyeongsan in southeastern Korea is notable for the large number of tombs constructed within ~100 years (fourth and sixth centuries CE) and widespread practice of human sacrifice. Analyzing genome-wide data from 78 individuals, we detected 11, 23, and 20 pairs of the first, second, and three-or-more-distant degree relatives, respectively, revealing a dense network of kinship in the Imdang-Joyeong society. We found five individuals from closely related parents, suggesting the practice of consanguineous marriage in both grave owners and the sacrificed. We also observed adult female descendants buried together with their kin, unlike several recent archeogenetic studies in Europe reporting a strict pattern of female exogamy. We detected no discernible genetic difference between grave owners and the sacrificed. Our analysis provides bioarcheological information on the burial customs and social structure of the Three-Kingdoms period society in Korea.

## Teaser

Ancient genomes reveal a dense network of sex-unbiased kinship and endogamy in a fourth to sixth century CE society in Korea.

## INTRODUCTION

Understanding kinship practices in ancient societies is crucial for reconstructing their social organization and cultural practices. Although kinship is a complex interplay between biological and social practices, biological kinship can provide means for this reconstruction. Recent advances in genome-scale ancient DNA (aDNA) studies allow an accurate reconstruction of biological kinship between buried individuals and parental relatedness. While studies on European cemeteries, ranging from the Neolithic to Medieval periods (*1*–*6*), have revealed a common pattern of patrilocal descent and female exogamy, studies outside of Europe are scarce. Ancient genome studies of biological kinship outside ancient Europe provide cases against the generalization of patrilocality with strict female exogamy as a prevalent practice worldwide. For example, the genomic evidence of matrilocal descent has been reported in the prehistoric society in the southwestern United States (*7*), a Neolithic society in eastern China (*8*), and a Neolithic settlement in Anatolia (*9*). There are also a few cases in which ancient societies commonly practiced consanguineous marriage within family groups (*10*), such as a prehistoric Aegean society (*11*) and two cases from Neolithic societies in China (*8*, *12*).

The Three-Kingdoms period of Korea (ca. 57 BCE to 668 CE) encompasses the history of three major political entities—Goguryeo, Baekjae, and Silla—which reigned over the Korean Peninsula and parts of present-day northeast China (*13*, *14*). Despite their geographical proximity, the three kingdoms differed in burial customs and marriage practices. Specifically, early Silla burials practiced Sunjang, a coburial of sacrificed human individual(s) together with the grave owner (*14*–*16*). Such an act of human sacrifice has been widely recorded across the globe, stemming from different cultural contexts (*17*–*19*). Different motivations for this mortuary practice have been suggested including, but are not limited to, resource conflicts (*20*, *21*), ritualistic practices (*22*), and justification of the accumulation of wealth and power (*23*, *24*). Sunjang in Silla burials are archeologically detected between the late third century CE to the early sixth century CE, consistent with the historical record of its prohibition in 502 CE by royal decree (*25*, *26*). Consequently, multiple sacrificial burials have been found in southeastern Korea, where the ancient Silla kingdom was centered (*27*). Silla is also thought to have practiced different marital customs from that of its neighbors, such as Goguryeo. Most notably, Silla royal elites are documented to have practiced consanguineous marriage, which is rarely observed in Goguryeo and Baekjae records. Historical accounts of consanguineous marriage are thought to be related to the consolidation of the rank and social status within Silla royals and local elites (*28*, *29*). In turn, royal families in Goguryeo have records of practicing levirate marriage, in which the brother of a deceased man married his widow (*30*), while no such cases have been documented in the historical records of Silla. However, because of limited ancient genome studies in Korea (*31*–*33*), no corroborating genomic evidence so far has been reported regarding the marriage customs of the Three-Kingdoms period Koreans, let alone Silla. Among these few studies, one reported a coburied family containing extended paternal and maternal relatives (*31*), suggesting a possibility that some Three-Kingdoms period Koreans may have had a family structure with limited sex bias.

**Fig. 1.**
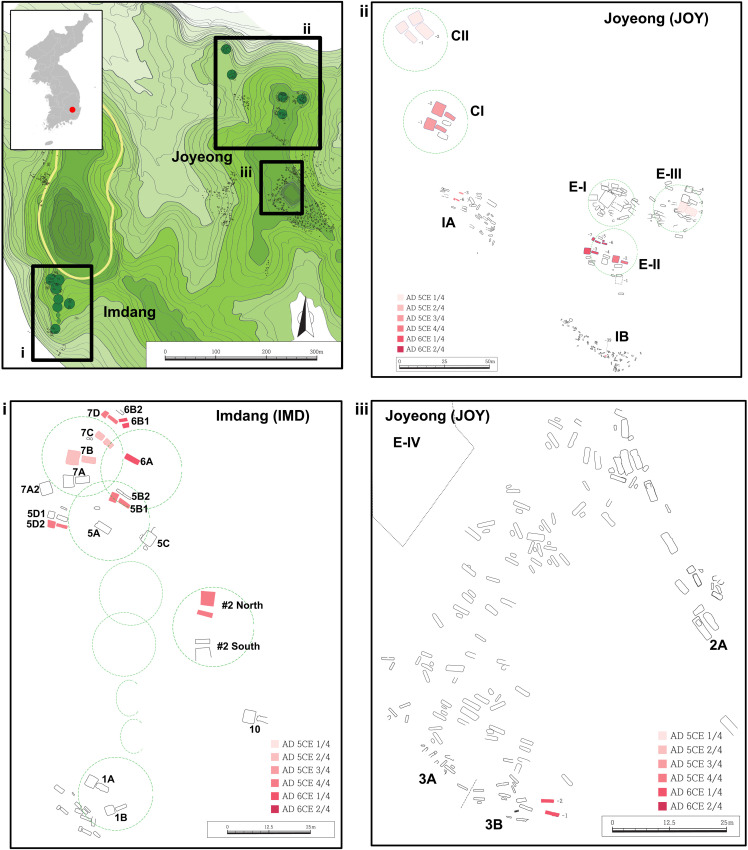
The location of the Imdang and Joyeong burial site analyzed in this study. The three main geographical locations of the tombs consisting of the Imdang-Joyeong burial complex with separate zoom-in panels (i to iii). The green gradient represents elevation, and the green circles represent the position of dirt mounds of the tombs. Estimated time periods of burials are based on archeological evidence and are depicted by color gradient.

The Imdang-Joyeong burial complex, located in Gyeongsan City, Gyeongsangbuk-do Province, Republic of Korea, is among the most famous archeological sites in the Three-Kingdoms period Korea and provides many cases of sacrificial burials of Silla. The complex comprises three sites, Imdang, Joyeong, and Bujeok. Among the three sites, the Imdang and Joyeong sites have undergone extensive archeological excavations since the first excavation of 1982 led by the Yeungnam University Museum (*34*) (Fig. 1 and text S1). So far, more than 1600 graves, 25,000 burial goods, and human remains of 259 individuals have been recovered from the Imdang-Joyeong burial complex. The burial complex includes consecutively constructed graves from the fourth to sixth century, spanning 100 years, which corresponds to approximately three to four human generations. (*34*, *35*). The owners of the graves are assumed to be of Silla origin and were local ruling family groups descended from elites of Abdok, a small state assimilated into Silla during the fourth century CE (*36*).

At least 20 tombs made of two chambers show signs of Sunjang, consisting of a rectangular main chamber (jukwak) containing both the grave owner and sacrificed individuals and a square subsidiary chamber (bukwak) containing only sacrificial individuals if any (*15*, *35*, *37*). Both chambers typically contain one or two sacrificed individuals, although up to five individuals have been identified in two main chambers and four individuals in one subsidiary chamber (*35*). The abundance of graves constructed during a short period and burial of different statuses of individuals provides an opportunity to study the ancient mortuary practices and shed light on past social structures of Three-Kingdoms period Korea. While the Imdang-Joyeong society may not represent the Three-Kingdoms period Korea nor the Silla Kingdom, they still provide a rare opportunity to study the structure of a local Silla society from a bioarcheological perspective. A previous study using isotopic analysis was able to differentiate the dietary patterns between Imdang-Joyeong grave owners and sacrificed individuals, suggesting a socioeconomic stratification between elite members and sacrificed individuals (*14*, *16*). However, aDNA studies on the Imdang-Joyeong complex to discover patterns of kinship are lacking. While there have been two previous studies using mitochondrial DNA (*38*, *39*) that provided insights into the social structure of the Imdang-Joyeong ancients, these analyses alone were not powerful enough to reconstruct exact pedigrees due to the limitations of only using uniparental haplogroups. Therefore, key questions regarding the Imdang-Joyeong ancients remain unresolved: What are the familial relationships between the buried Imdang-Joyeong ancients? Do sacrificed individuals share kinship with each other? Is there a genetic differentiation between grave owners and sacrificed individuals? Did the Imdang-Joyeong ancients practice close-kin marriage (hereafter “endogamy” for brevity) as documented for the Silla Royal elites?

In this study, we present genome-wide data from 78 individuals across 44 graves, reconstructing an extensive kinship network of ancient individuals from the Imdang-Joyeong site. Individuals born from consanguineous marriage were observed from both grave owners and sacrificed individuals, supporting endogamous practices regardless of social status. We also find similar kinship connectivity between adult males and females, suggesting that the Imdang-Joyeong ancients provide a rare example of a society with a substantial level of endogamy with limited gender bias. Last, we show that the Imdang-Joyeong ancients had no detectable difference in genetic profiles between the Imdang and Joyeong burial sites and buried status.

## RESULTS

### Curation of data

We screened genomic DNA from 182 skeletal elements of 172 individuals from the Imdang-Joyeong site, which included human petrous parts, femur, tibia, teeth, and other skeletal remains (fig. S1 and data S1). The names of skeletal elements were given the prefix IMD if they were from the Imdang burial site and JOY if they were from the Joyeong site. On the basis of archeological evidence, 97 elements were assigned to grave owners, among which 32 elements from the two-chambered tombs. Furthermore, 61 elements were classified as bones from sacrificed individuals, and the remaining 24 skeletal elements were ambiguous of grave ownership. Of these, we selected 85 skeletal elements with sufficient endogenous human DNA (>0.1%) and clear signs of aDNA postmortem damage patterns expected for the corresponding library preparation protocols. We applied in-solution DNA capture targeting 1,233,013 single-nucleotide polymorphisms (SNPs) from a panel commonly used for human population genetics (“1240K”) (*40*–*42*). Considering skeletal elements from the same individual and merging information of duplicate libraries (data S1 and S2), genome-wide data were obtained from 78 individuals; 25 individuals (4 grave owners, 12 sacrificed, and 9 of ambiguous burial status) were from the Imdang site, and 53 individuals (22 grave owners, 26 sacrificed, and 5 of ambiguous burial status) were from the Joyeong site. On the basis of the sequence read depth ratio between sex chromosomes and autosomes, 34 individuals were identified as males, 42 were identified as females, and 2 were of ambiguous sex (figs. S1 and S2). By making pseudo-haploid genotype calls for 1240K sites using the pileupCaller program, we retrieved between 5551 and 1,060,866 ancestry-informative SNPs covered by at least one high-quality read for these 78 individuals. To avoid contamination affecting our analyses, we flagged 14 individuals being potentially contaminated. Specifically, 4 individuals were flagged as contaminated based on ≥5% mitochondrial (MT) contamination (JOY012, JOY073, JOY076, and JOY078), 2 individuals based on ≥5% X chromosomal contamination for males (IMD023 and IMD024), 2 individuals with ambiguous genetic sex (IMD033 and JOY083), and 6 individuals who failed all contamination estimates (IMD039, JOY054, JOY106, JOY107, JOY121, and JOY123). For kinship analysis, we used information of all 78 individuals regardless of flag. We include contaminated individuals because contamination is unlikely to generate false-positive kinship between two individuals unless they are both contaminated by the same individual. No kinship closer than fourth degree was called for any two individuals that both had contamination or had no available contamination estimate. We also ignored third-degree or further kinship estimates between pairs that included an individual with coverage lower than 0.05× on 1240K SNP sites to avoid false-positive kinship signals based on the performance of KIN on low-coverage samples (*43*). For population-based analyses, we excluded the flagged 14 individuals with potential contamination and an additional 8 individuals with first degree kinship with another individual (IMD001, IMD031, JOY003, JOY011, JOY018, JOY035, JOY077, and JOY118), retaining 56 individuals. Last, 32 individuals with more than 400,000 called 1240K SNPs were used for hapROH and ancIBD analysis.

### Evidence of endogamy from the Imdang-Joyeong kinship pedigree

**Fig. 2.**
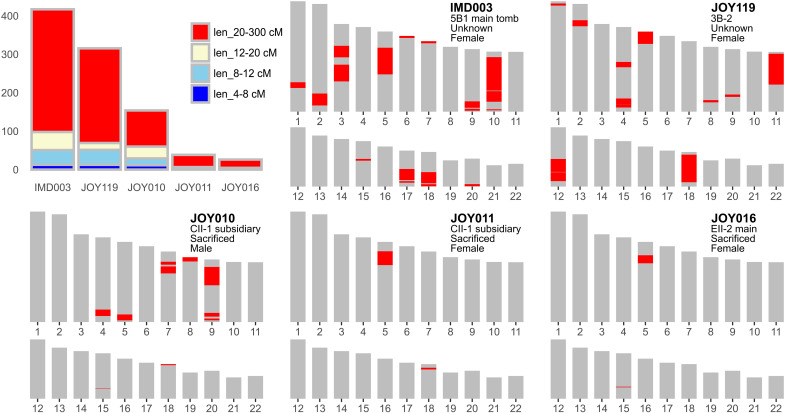
ROH distribution of five Imdang-Joyeong ancients. We present the cumulative genetic length and physical position of ROH for five Imdang-Joyeong ancient individuals with ROH length longer than 20 cM. For each individual, the physical position of the ROH segments (red segments) is displayed on the autosomes (gray bar) based on genome coordinates from GRCh37, base coordinates starting from bottom to top.

**Fig. 3.**
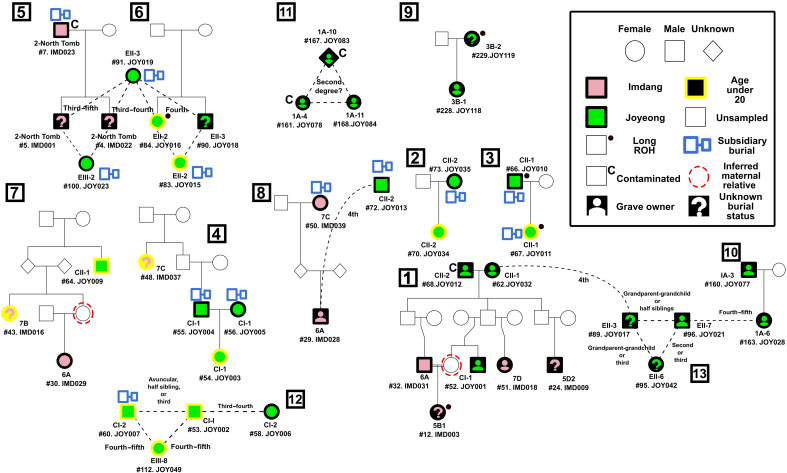
Reconstructed kinship relationships between Imdang and Joyeong ancients. We present the likely pedigrees and kinship relationships of 42 individuals. Family groups closer than third degree are numbered in groups indicated by number in squares (families 1 to 13). Relationships that were unable to exactly pinpoint were expressed as dotted lines. Individuals are labeled by their tomb, archeological report number, and sample ID. Individuals that are colored pink (Imdang) or green (Joyeong) are sampled individuals, while individuals with no colors are unsampled individuals. Yellow lines surrounding a shape represents that the individual’s age of death was under 20. Shapes next to the individual depict their burial status.

Previous studies have suggested the possibility of sacrificed individuals buried in the same tomb sharing close kinship (*15*, *35*, *37*). However, there has been no attempt to test these claims. To detect close biological relatedness between Imdang and Joyeong ancients, we used KIN (*43*), which can identify close genetic relationships within our low-coverage aDNA dataset. In addition, we considered MT and Y haplogroups to rule out certain implausible relationships leveraging their uniparental inheritance pattern, and the number and total length of long runs of homozygosity (ROH) genomic segments to identify individuals born from consanguineous marriage (Fig. 2, figs. S3 and S4, and data S1 and S4). Considering the biological information including osteological age of death estimates, along with archeological information such as grave construction order, we found 54 pairs of family relationships between 42 individuals: 11 first degree, 23 second degree, and at least 20 third or more distant degrees of relatedness (data S4). This expectedly large number of genetic relatives enabled us to recreate 10 pedigrees with individuals connected by at least one second degree relationship (families 1 to 10) and three more groups of individuals who were connected by second to third degree relationships (families 11 to 13) (Fig. 3 and text S2). We note that some of these families are also connected, in that three pairs of families share third to fourth degree relationships through certain family members. While the Imdang and Joyeong burial sites were geographically separated from each other (Fig. 1), we observe that family relationships spanned both burial sites for two pedigrees by a second-degree relationship, and more distant relations around fourth degree existed between the two sites for three cases, suggesting close connectivity between the two burial sites (Fig. 3). Last, we only detect two individuals who had within third-degree kinship with an individual of different burial status from a single grave (grave 6A; grave owner: IMD028 of family 8, sacrificed: IMD031 of family 1), suggesting kinship stratification between the grave owners and sacrificed individuals.

Based on our pedigree, we analyzed each family to find patterns of interest. We first investigated family 1, a family made mostly of grave owners with seven individuals closely related up to second degree. This pedigree, starting from the founder grave owner pair JOY012 (male) and JOY032 (female), spans four generations. The founders were buried in two adjacent graves (Joyeong CII-1 and CII-2), which were already suggested to be couple’s graves before this research (*37*). The second-generation individuals were not sampled, while the third and fourth generation individuals were mostly located in separate graves. This was different from the burial patterns of sacrificed families, where parents and children were coburied in the same tomb (families 2 to 5).

An interesting individual of the grave owner family is a female individual IMD003. IMD003 is the daughter of male IMD031 and the unsampled sister of male JOY001, sharing the mitochondrial haplogroup C4a1a with JOY001, and exhibits multiple long ROH segments (a total of 319.22 centimorgan (cM) of ROH segments longer than 20 cM), corresponding to their parents being a first cousin or closer relationship (Fig. 2 and fig. S5). IMD003 provides direct evidence of close consanguineous marriage among grave owners, which may reflect a shared cultural practice among the Silla people consistent with textural records (*28*, *29*). IMD003 is not the only individual who was born from these consanguineous marriages. We found four more individuals with ROH longer than 20 cM, although we were unable to specify the exact familial relationship of their parents unlike IMD003 (Figs. 2 and 3 and fig. S4). Note that we observe consanguinity not only for grave owners but also for sacrificed individuals (e.g., JOY010 and JOY011 of family 3), which suggests practices of consanguineous marriage regardless of social status. Last, we found no case matching a pedigree pattern expected from levirate unions, a common practice in pastoral societies such as the Xiongnu and Avar (*6*, *44*), as expected from lack of textural evidence for such practice in Silla unlike Goguryeo, which had heavy ties with pastoralist societies (*30*).

To detect patterns of exogamy, we first investigated the distribution of uniparental haplogroups (fig. S3 and data S1). We found that male grave owner individuals mostly had Y haplogroup O1b2a, which was also the most common Y haplogroup in our dataset. In contrast, mitochondrial haplogroups were much more diverse compared to Y haplogroups (fig. S3). While these findings may superficially seem to support a patrilocal family structure for grave owners (6 of the 13 males), we observe the haplogroup O1b2a in 8 of the 13 sacrificed males as well, suggesting that this haplogroup may have been a common one at the population scale. O1b2 is a common Y haplogroup found in present-day Koreans. Previously called O2b under the International Society of Genetic Genealogy (ISOGG) nomenclature (*45*, *46*), O1b2 has been reported at high frequencies in unrelated Korean males, consisting ~22 to 39% of the total Y haplogroup distribution (*47*–*49*).

While lower haplogroup diversity of Y chromosome than mitochondria may be consistent with a patrilocal family structure, there is evidence for the opposite. Specifically, we observe cases of adult females sharing inferred maternal relatives (Fig. 3). For example, females IMD016 (osteological age of 8.5 to 13.5 years) and IMD029 (21 to 35 years) of family 7 shared the same mitochondrial haplogroup and were second degree relatives (likely of avuncular relationship), and both were relatives with male JOY009. This suggested the existence of an unobserved maternal relative shared between IMD016 and IMD029, providing evidence of an adult female sharing kinship with a coburied individual through a maternal lineage. Another inferred maternal relative of an adult female is the mother of IMD003, who married a full cousin (family 1). We also observe cases of adult female individuals with their own graves having distant relatives such as IMD003 and IMD018 (family 1) and JOY006 (family 12). Last, we report a case of a father-daughter pair where the daughter (JOY028) was buried with her fetus (family 10). These findings suggest the possibility that there may have been limited sex-based exogamy in the family structures of Imdang-Joyeong ancients.

### Similar level of genetic connections between adult males and females

**Fig. 4.**
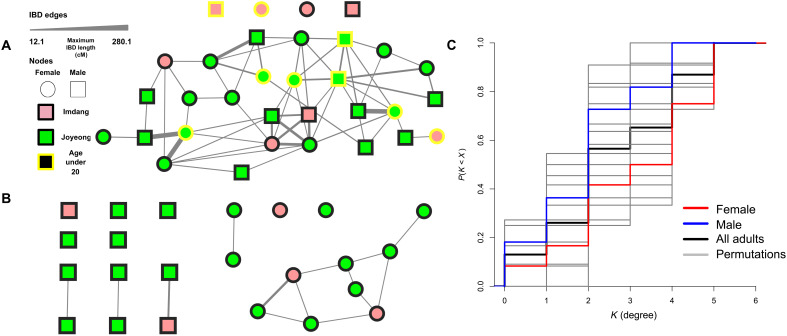
Analysis of IBD network sharing between Imdang-Joyeong individuals based on ancIBD results. (**A**) Network visualization of IBD connections (edge) between individuals (node). The color of the node represents the burial site, while the color of the outline being yellow indicates the age of death under 20. The width of the edges (strength) represents the maximum IBD length shared between the two individuals. (**B**) Networks of adult male individuals only (left) and adult female individuals only (right) networks. (**C**) Network statistics of the adult-only network. Degree of connection refers to the number of connections a node has. Empirical cumulative distribution function (eCDF) of degree of connection being smaller than K. Red lines depict females, blue lines depict males, and gray lines are eCDFs for 1000 permutations on node sex labels.

Our pedigree analysis was limited to detecting relationships up to the fourth degree relatives. To further analyze kinship relations between Imdang-Joyeong individuals, we analyzed the identity-by descent (IBD) segment sharing between 32 individuals using ancIBD (Fig. 4A and fig. S7). We created the network of IBD sharing using the maximum IBD segment length more than 12 cM as weights for edges and individuals as nodes. We compared the distribution of the number of connections (degree) and the sum of the maximum IBD (strength) of a node between sexes (Fig. 4C and data S5). For an exogamous matrilocal society, one would expect more IBD connections and higher degree centrality between adult females than males. For an exogamous patrilocal society, an opposite pattern would be expected. We find a lack of evidence to differentiate between the distribution of degree and strength of connection in 11 adult males and 12 adult females within the adult-only IBD network (Fig. 4C and data S5). These findings, along with the finding of five individuals with ROH lengths longer than 20 cM, suggest that the Imdang-Joyeong complex ancients practiced endogamy with limited genetic sex bias.

### Depleted long IBD sharing between grave owners and sacrificed individuals

We analyzed patterns of IBD sharing between grave owners and sacrificed individuals. We first removed individuals with unknown burial status, excluding IMD003 and JOY119. IMD003 and JOY119 were considered as grave owners, as their ambiguity of burial status resulted from their association with other coburied individuals later identified as duplicates in this study (text S1), by categorizing the edges of the IBD network into three groups, “Within Sacrificed” and “Within Owners” for edges connecting the node pairs from the same burial status and “Between” if the node pairs are of different burial status (fig. S8). We found that while grave owners and sacrificed individuals share short IBD segments of length 12 to 32 cM, IBD segments over 32 cM are nonexistent. These findings suggest that while some background biological relatedness was shared between the two groups, which could either be grave specific or population-wide, closer biological relatedness between grave owners and sacrificed individuals were scarce or depleted, contributing to the genetic stratification observed between the two burial classes.

### Homogeneous genetic profiles of Imdang-Joyeong ancients

**Fig. 5.**
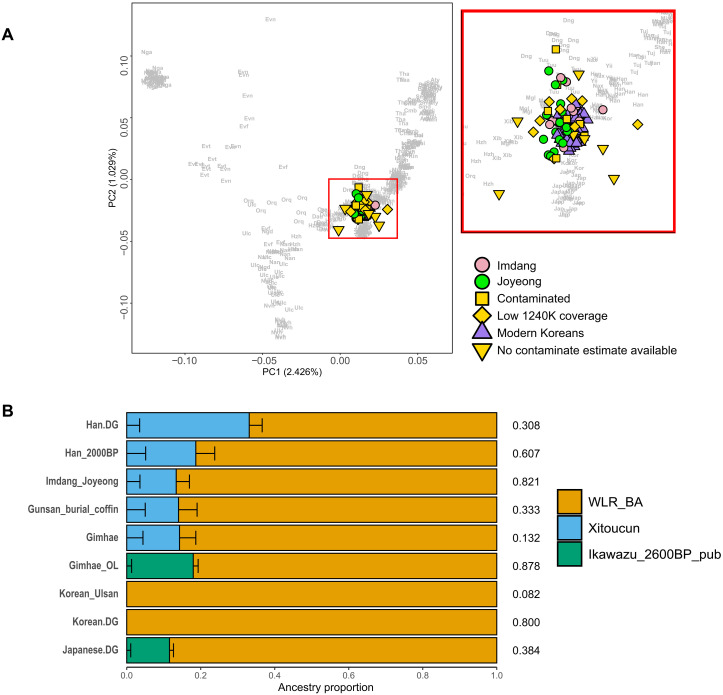
Genome-wide ancestry profile of Imdang-Joyeong ancient individuals. (**A**) “East Eurasian” PCA for Imdang-Joyeong ancient individuals and modern Koreans. Ancient individuals are depicted as squares, while modern individuals are labeled as circles. Individuals who were flagged as contaminated or had under 0.05× depth coverage for 1240K SNP are flagged as yellow shapes. Gray letters are three letter abbreviations of the modern populations used for calculation of PCA. The variances explained by the first two PCs are shown in brackets. (**B**) qpAdm modeling of Imdang-Joyeong ancients, published ancient Three-Kingdoms period Koreans, ancient East Asians, and present-day East Asians as a mixture of three sources: West Liao River Bronze age individuals (WLR_BA), Late Neolithic South Chinese (Xitoucun), and a Jomon from the Ikawazu shell-mound site (Jomon_Ikawazu). Horizontal error bars represent ±1 SE estimated by qpAdm through 5 cM of block jackknifing.

So far, only two studies have investigated the genome-wide genetic profile of ancient Three-Kingdoms period Koreans (*31*, *32*). Two of eight individuals from Gimhae city close to the southern coast of the Korean peninsula exhibited signals of admixture from Jomon-related populations (*32*). On the basis of these previous observations, we investigated the possibility of substructure within our Imdang-Joyeong ancients. We did principal components analysis (PCA) following the methods of (*50*), projecting ancient individuals onto the top PCs of 378 present-day Eastern Eurasians (Fig. 5A). We observe Imdang-Joyeong ancients closely clustering with present-day Koreans, except for low-coverage or contaminated individuals. This was also observable when we projected ancient individuals onto the top PCs of 2077 present-day Eurasians (fig. S9). To further detect shared genetic drift between Imdang-Joyeong ancients and published individuals, we calculated outgroup-*f*3 statistics in the form *f*3(Mbuti; Imdang-Joyeong, worldwide) (fig. S10 and data S6). The Imdang-Joyeong ancients showed high shared drift with present-day Koreans and published Three-Kingdoms period Koreans. We also find no significant difference in ancestry depending on buried status or burial site using *f*4 statistics *f*4(Mbuti, world-wide; sacrificed individuals, grave owners) and *f*4(Mbuti, worldwide; Imdang ancients, Joyeong ancients) (fig. S11 and data S7 and S8). These findings suggest homogeneous genetic makeup within the Imdang-Joyeong burial complex.

To further test the homogeneity of Imdang-Joyeong ancients, we formally modeled the genetic profiles of Imdang-Joyeong ancients and other ancient Three-kingdom period Koreans using the qpAdm-based admixture modeling (Fig. 5B and data S9). We performed qpAdm using distal sources as (*31*). We used Bronze Age ancient humans from the Longtoushan site (“WLR_BA”) as a northern East Asian source, and Late Neolithic ancients from the Xitoucun site of southern China (“Xitoucun”) as another source. We also checked whether including a Jomon individual from the Ikawazu site (“Jomon_Ikawazu”) as the third component of ancestry can improve model fit. A two-way modeling using WLR_BA and Xitoucun as a proxy was plausible for all Three-Kingdoms period ancient Koreans, excluding the two Gimhae outlier individuals. Including Jomon_Ikawazu as the third ancestry only improved the fit for the two Gimhae outlier individuals, while the other ancient Koreans did not require Jomon_Ikawazu as the third source. On the basis of these findings, we concluded that Imdang-Joyeong ancients did not differ from other Three-Kingdoms period Koreans and showed no sign of admixture from a Jomon-related source. Still, reflecting geographic distance, we did not detect kinship within fourth degree between the Imdang-Joyeong ancients with other Three-Kingdoms period Koreans, i.e., those from Gimhae Daeseong-dong, Yuha-ri, and Gunsan Dangbuk-ri sites (data S10). These findings suggest that the Imdang-Joyeong ancients share a genetic continuity that reflects a stable population structure in southern Korea during the Three-Kingdoms period.

## DISCUSSION

On the basis of the genomic DNA of 78 individuals, we find an extensive kinship network from a fourth to sixth century burial complex in Korea, spanning two burial sites Imdang and Joyeong. We present the first genetic pedigree of the Imdang-Joyeong ancients consisting of 13 families where individuals were connected by up to third-degree relative pairs. Our pedigree of the Imdang-Joyeong ancients provides valuable insights into the archeology of the Imdang-Joyeong complex. One area of this contribution is to decide the construction order of graves within the Imdang-Joyeong complex. For example, we found cases of the offspring’s grave buried close to the parent (Fig. 3, families 9 and 10). By leveraging on the generational sequence of grave owners, our pedigree can be coanalyzed with archeological evidence to provide valuable insights for predicting the construction age of consecutive graves.

The parents of the individual with the longest ROH, IMD003, were first cousins or closer (Fig. 2 and figs. S3 and S4). Four more individuals show evidence of consanguineous marriage, of which two were sacrificed father and daughter buried in a subsidiary chamber together suggesting consanguineous marriages in two successive generations. Consanguineous marriage is commonly associated with societies practicing intracommunity marriage, where unions are made within specific societal groups (*51*, *52*). The observation of consanguineous individuals in both grave owner families and sacrificed individuals suggests that the Imdang-Joyeong society practiced intracommunity marriage, likely within social status.

Our genetic analysis is in line with previous suggestions of familial relationships for both grave owners and sacrificed individuals. We found a marital relationship between the owner of two graves CII-1 and CII-2 (Figs. 1 and 3; family 1, JOY012 and JOY032), confirming previous suggestions of a marital relationship based on archeological evidence (*35*, *37*). The two graves were constructed parallel to each other with similar sizes, same chamber direction, and same burial construction style, with each grave containing gender-specific burial goods (*35*). Considering the case of CII-1 and CII-2, similar parallel pairs of graves in the Imdang-Joyeong burial complex, such as #2 North and #2 South tombs, may be likely marital tombs as well, although all skeletal elements from the main chambers of the two tombs were of ambiguous burial status. Specifically, the two individuals from #2 North tomb main chamber (IMD001 and IMD22) were brothers and they were sons of a male buried in the subsidiary chamber (IMD023) (family 5). This suggests that the possibility of the grave owners of at least one of the tombs is yet to be sampled to validate this hypothesis. We also found decisive evidence of three cases of families in which parents and their offsprings were sacrificed together in the same grave (Fig. 3; families 2 to 4). Our genetic findings are the first to confirm the acts of Sunjang of an entire household and suggest that these practices might be common for sacrificial burials of the Three-Kingdoms period. Genetic relatedness among sacrificial individuals over generations may suggest the presence of families that served as sacrificial individuals for the grave owner class for consecutive generations. The endogamous kinship structure, along with the possibility of sacrificial families, suggests a culture of strong inheritance of social status through kinship. A proto–Three-Kingdoms period sacrificial grave located at Jisan-dong, Goryeong, is suggested to have entire families being sacrificed based on archeological evidence (*53*); thus, it may show a root of this practice, although the family relationship remains to be tested (*35*).

Our study is limited in that multiple individuals are of ambiguous burial status, which may constrain our results. In addition, the relatively small numbers of successfully analyzed individuals compared to the total number of buried individuals limits us to generalize our findings. The small number of samples is limited by the preservation of aDNA in our samples, which is restricted due to the acidic soil environment of the Korean peninsula. Continued research is therefore crucial to obtaining higher-quality data from the ancient Korean populations. Last, we caution that the observed kinship practices of the Imdang-Joyeong society may not be a general practice by other local societies of the Silla Kingdom nor the broader Three-Kingdoms period Korea. An interesting venue of future research could be archeogenomic analysis of contemporaneous burial sites around the Imdang-Joyeong burial complex. For example, the excavation sites at Daedong and Bujeok in Gyeongsan city are of the same archeological context as the Imdang-Joyeong burial complex, which may show genetic connection at a regional scale. Also, the excavation site at Goejeon-dong, Daegu city provides another venue of analysis of a Silla context burial site. These sites around the Imdang-Joyeong burial complex have the potential to uncover intercommunity kinship connections and to test the representativeness of the Imdang-Joyeong society, shedding light to the unknown local kinship practices of Silla.

A previous study on fourth to fifth CE Three-Kingdoms ancient Koreans from the Daeseong-dong tumuli of Gimhae City reports two individuals with Jomon-related ancestry, who were likely immigrants or their descendants from the Japanese archipelago (*32*). While the Imdang-Joyeong individuals cannot be generalized for all Three-Kingdoms period ancient Koreans, our findings of previously published Three-Kingdoms period Koreans (*31*) and Imdang-Joyeong ancient individuals having no Jomon affinity suggest that it is unlikely a general characteristic of the Three-Kingdoms period southern Korea to have the immigrants from the Japanese archipelago or their recent descendants. Archeogenetic studies on more sites from the Three-Kingdoms period Korea and Kofun period Japan are required to delineate migration patterns between Korea and Japan during this period.

Our research is the first to analyze the genome-wide composition of closely related individuals from an ancient Three-Kingdoms period of Korea. Through genetic data, we present evidence of a distinctive family structure that differs from patrilocal systems observed in ancient Europe. We also present a case of high consanguinity within a local society, which is in contrast with contemporary societies of the East Asian steppes such as the Avars (*6*). In addition, we demonstrate that ancients of the Imdang-Joyeong burial site exhibited genetic homogeneity, irrespective of burial context. We believe further archeogenetic studies on the Korean peninsula will reveal more information on the population dynamics and family structures of ancient East Asia.

## MATERIALS AND METHODS

### Archeological context of the Imdang-dong and Joyeong-dong ancient burial grounds

Imdang-dong and Joyeong-dong are in an alluvial plain in Gyeongsan City, Republic of Korea. The burial ground served as a cemetery for the local ancient Korean population, containing ~1600 burials, of which 182 tombs have been identified and selected for this study. The construction age of the tombs ranges from fourth to seventh century CE and were constructed by digging out the bedrock and creating a wooden chamber within the excavated area. Mud and water seeped into the chambers from the bedrock, preserving the bones of the humans and animal carcasses buried within. Among the 182 tombs, 20 tombs differ in size with the rest of the tombs, each consisting of two chambers with evidence of sacrificed individuals coburied with the owner. The chambers also contained burial goods, ritual vessels, and food for the deceased. The size of the tomb and the act of human sacrifice indicate the high status of its owners. The owners of the tombs are local elites, being descendants of the Abdok state assimilated into Silla during fourth century CE.

### Ancient DNA laboratory work and sequencing

We extracted genomic DNA for 182 skeletal samples representing 172 individuals excavated from the Imdang and Joyeong burial complex. For each sample, we extracted metagenomic DNA from 50 mg of bone powder and prepared double-strand or single-strand double-indexed Illumina sequencing libraries. Ninety-two samples including 69 petrous parts of the temporal bone, 21 femur, and 2 tibiae were powdered by a dental drill at the Yeungnam University Museum, South Korea. The remaining 77 teeth and 11 small bones were powdered at Max Planck Institute, Germany. DNA extraction and library preparation were conducted in accordance with published and established protocols (*54*, *55*). Genomic DNA of 46 samples (33 petrous and femur, 11 teeth bone extract, and 2 bones) were extracted from the Max Planck Institute for the Science of Human History (MPI-SHH; current Max Planck Institute for Geoanthropology), Jena, Germany. The genomic DNA of 136 ancient human samples (36 petrous, 21 femur, 2 tibiae, 66 teeth, and 11 bones) were extracted from the Max Planck institute for Evolutionary Anthropology (MPI-EVA), Leipzig, Germany. Laboratory works up to library preparation were performed in specialized clean rooms dedicated to processing aDNA. Samples processed at MPI-SHH were used to build double-strand libraries, while samples processed at MPI-EVA were used to build single-strand libraries. For samples processed at MPI-SHH, we included partial treatment of the uracil-DNA-glycosylase (UDG) enzyme based on previously published protocol (*56*) to restrict base deamination to the 5′ and 3′ ends of the reads. After an initial round of sequencing, 85 samples exhibiting human DNA preservation levels between 0.1 and 88.8% were selected for further analysis. The libraries were then enriched using in-solution capture for 1,233,013 ancestry-informative SNPs (the “1240K panel”) (*40*–*42*). All sequencing of libraries were conducted using single-end 76–base pair (bp) sequencing on the Illumina HiSeq 4000 platform, in accordance with the manufacturer’s protocols.

### Processing and authentication of aDNA

The sequenced aDNA data were first trimmed to remove Illumina adapter sequences from raw reads using AdapterRemoval v2.3.0 (*57*). Adapter-trimmed reads shorter than 35 bp were removed to exclude human-prokaryote ambiguous reads (*58*). Remaining adapter-trimmed reads were mapped to the human reference genome with decoy sequences (hs37d5) using the aln/samse modules in BWA (Burrows-Wheeler aligner) v0.7.17 with “-n 0.01” option (*59*). Reads that were not treated with UDG were mapped with additional parameter “-l 9999” to disable seeding considering the high amount of deamination of bases. PCR duplicates were removed using DeDup v0.12.5 (*60*). Uniquely mapped reads with Phred-scaled mapping quality score 30 or higher were kept using Samtools v1.9 (*61*) for downstream analysis.

We reviewed the authenticity of our dataset by analyzing postmortem deamination and contamination of DNA. We first tabulated the postmortem base deamination patterns typical of aDNA molecules using mapDamage v2.0.9 (*62*). We then estimated nuclear and mitochondrial contamination levels using the ANGSD (analysis of next generation sequencing data) contamination module v0.937 (*63*) and the schmutzi program (*64*).

### 1240K SNP panel genotyping

To reduce the biases from low coverage and cytosine deamination, we made pseudo-haploid genotype calling considering the damage pattern of our libraries. The two bases of the 5′ and 3′ ends of each read were soft-masked for partially UDG treated double-strand libraries using the trimBAM module of bamUtils v1.0.14 (*65*) to remove 5′ C>T and 3′ G>A deamination. After creating mpileup files with Samtools mpileup with options -B, -R, -q30, and -Q30, we used the pileupCaller program v1.5.2 with option “--randomHaploid” to randomly select a high-quality base (base quality score 30 or higher) from a high-quality read (mapping quality score 30 or higher) for each SNP locus and use the chosen base to represent a pseudo-haploid genotype. For single-strand libraries, we included the “--singleStrandMode” flag when running pileupCaller. Then, we detected duplicate samples from the same individual based on the pairwise mismatch rate (PMR) (*7*) of pseudo-haploid genotypes for all pairs of libraries: Duplicate pairs show approximately half of the PMR values (0.118), while pairs from unrelated individuals have (0.235), twice the value of the duplicates. After detecting duplicates, BAM files of the same library were merged and were genotyped again to create pseudo-haploid genotype data per individual. For two individuals with libraries with different UDG treatments, genotypes were merged by filling in missing SNPs of the low missing library’s genotype with the other library’s genotype.

### Processing whole-genome sequences of present-day Koreans

We follow the procedures of a previous study (*31*) to process whole-genome sequence of 104 Koreans from the KoVariome dataset (*66*). On the basis of PCA and genetic relatedness estimated using PMR, we filter out two genetic outliers and one individual from each relative pair up to the second degree.

### Data curation

The generated genotype data of Imdang-Joyeong ancients were merged with the present-day KoVariome data and previously published datasets of modern and ancient individuals genotyped on the 1240 K SNP panel for general population genetics analysis. Specifically, we include 11 unrelated Three-Kingdoms period ancient individuals (*31*, *32*) for comparative analysis with contemporaneous Imdang-Joyeong ancients. For PCA, the genotype data of Imdang-Joyeong ancients were merged with genome-wide genotype data of 2077 present-day Eurasians genotyped on the Affymetrix Axiom Genome-Wide Human Origins 1 Array. Information on population labels and publications are provided in data S3.

### Per individual assessment

The autosomal, X, Y, and MT coverage of each individual was calculated at SNP positions from the 1240K panel and whole mitochondrial positions. The genetic sex of each individual was determined by comparing the coverage of autosomal and sex chromosome regions. Specifically, we compare X-to-autosome depth ratio against Y-to-autosome depth ratio to decide genetic sex. We then assigned MT and Y chromosomal haplogroups. We first generated the mitochondrial consensus sequences for endogenous reads with quality of 10 or higher (“-q 10” flag) using the log2fasta program in the Schmutzi package. We then used HaploGrep2 v2.1.20 (*67*) to assign MT haplogroups. For Y chromosomal haplogroups of the males, we used pileupCaller v1.4.0.5 option “--majorityCall” to call two sets of SNPs: 13,508 Y chromosome SNPs retrieved from the ISOGG Y-DNA haplogroup tree v.11.04 and 36,152 Y chromosome SNPs retrieved from the ISOGG Y-DNA haplogroup tree v.15.73 (*68*). We then used a modified version of the yHaplo program (*69*) to call Y haplogroups of male samples. We used “-ancStopThresh 10” following the developer’s recommendations.

### Testing consanguinity (ROH)

Consanguineous individuals have long portions of homozygous genome, also known as ROH. To estimate ROH of our pseudo-haploid data to understand the parental relatedness, we applied hapROH (*10*). Based on the recommended coverage range for samples of 400,000 or more 1240K SNPs covered by at least one high-quality read, we were able to estimate ROH length of 34 individuals.

### Estimation of genetic relatedness

To infer the genetic kinship between the Imdang and Joyeong individuals, we used the KIN software (*43*), which identifies genetic relatedness by inferring identity-by-descent (IBD) segments through a hidden Markov model approach. BAM files including high-quality unique mapped reads overlapping the 1240K panel positions were preprocessed and analyzed for ROH segments using KINgaroo, which is included in the KIN python package. We used default parameters with contamination correction disabled (-c 0) and provided a bed file specifying the position and reference/alternative alleles of the 1240K SNPs. KINgaroo returns the estimate of long runs of homozygosity to account for overestimation of close relationships due to consanguineous marriage.

We then ran KIN with the outputs of KINgaroo with default settings. KIN returns all log-likelihood scores for different cases of relatedness: unrelated, fifth degree, fourth degree, third degree, grandparent-grandchild, half siblings, avuncular, siblings, parent-child, and identical. Although the summary provided by KIN classifies relationships based on the highest log-likelihood value, categorizing distant relatives (fifth and fourth degree) as unrelated and avuncular, half siblings, and grandparent-grandchild as second degree, we consider all values for comprehensive analysis. Specifically, to account for the uncertainty of the estimate provided by KIN, we first filtered out all relationships where the log-likelihood difference between the model with the maximum log-likelihood value and the unrelated model was less than two. Then, we considered within a relationship models that only differ by one with the maximum log-likelihood value as a plausible relationship. We removed kinship estimates when either (i) the classification was third degree or farther with at least one individual with <0.05× 1240K SNP coverage or (ii) both individuals were contaminated or have no estimate of contamination available. We cross-checked our inference with PMR estimates, uniparental haplogroup information, ROH length, and archeological data such as estimated age of death and age of tomb construction.

### Imputation and phasing

We used GLIMPSE to impute and phase our data for IBD-based analysis, following the instructions given for the ancIBD pipeline (*70*). We first created genotype likelihood VCF files for biallelic SNPs from the 1000 Genomes Project Phase 3 reference panel using bcftools mpileup and bcftools call on bam files. For partial-UDG libraries, we used bam files masked 2 base pairs at the 5′ and 3′ end to exclude postmortem deamination damage. For single-strand non-UDG libraries, we calculated genotype likelihoods by separating the bam file into positive strand and negative strand bam files using samtools view while discarding C/T SNP calls for positive strand and discarding G/A SNP calls for negative strand. We ran GLIMPSE with default parameters and the HapMap b37 genetic map using the phased 1000 Genomes Project Phase 3 dataset as reference haplotypes. We used GLIMPSE_phase with imputation and phasing on 2,000,000 bp with buffer size of 200,000 bp. We then used GLIMPSE_ligate, GLIMPSE_sample, and bcftools to obtain imputed and phased vcf files with genotype posterior probabilities on the 1240K sites.

### IBD sharing analysis

To analyze IBD sharing, we used ancIBD (*70*). We used the imputed and phased data, filtering out SNPs with genotype posterior probabilities lower than 0.99 after imputation. The HapBLOCK function of ancIBD was used to perform estimation with default parameters with IBD blocks longer than 6 cM. IBD segments with 1240K SNP density lower than 220 per cM were excluded by default. IBD segments longer than 12 cM were considered to conservatively filter out false-positive IBD segments.

### IBD and ROH simulation

We simulated the distribution of IBD and ROH segments based on biological kinship using Ped-sim (*71*). We followed the methods described in (*71*) by incorporating a sex-specific recombination map (*72*) and a crossover interference model (*73*). For the IBD distribution, we simulated 14 familial relationships, including first-degree (parent-child, siblings), second-degree (grandparent-grandchild, avuncular, half-siblings, double cousins), first to third-degree full cousins, first to second-degree half cousins, and third to fifth-degree great-grandparent relationships. For the ROH simulation, we simulated the family relationships of the grave burial family containing seven uncovered individuals 1000 times to get the empirical distribution of ROH length for IMD003. The simulated results were the physical and genetic region of an IBD segment for each pair of individuals. Ped-sim also returns values IBD1 for one IBD segment sharing, IBD2 for two IBD segment sharing, and HBD for homozygous by descent in a segment region. To match the output with ancIBD, we merged neighboring IBD1 and IBD2 segments using a custom R script. We also filtered simulated IBD segments shorter than 12 cM or segments with fewer than 220 1240K panel SNPs covered per cM, which were the threshold used to filter false IBD segments using ancIBD.

### IBD network analysis

We used Cytoscape (v.3.10.2) to visualize the IBD network inferred by ancIBD and calculate network statistics for degree centrality. Specifically, we used “Analyze Network” under “Tools.” We used the R package igraph to calculate node strength, which was the sum of the maximum IBD segments per node. Statistical inference and permutation were done in R v.4.4.2 using custom scripts.

### Principal components analysis

We ran PCA on present-day individuals genotyped on the HumanOrigins array using smartpca v.18140 from the EIGENSOFT v8.0.0 software suite (*50*). We ran PCA for two sets of populations: present-day Eurasians (*n* = 2077) and East Eurasians (*n* = 378). Ancient individuals were projected onto the existing PCs using the option “lsqproject: YES” option (*74*).

### Population genetic analysis

We used the functions from R library ADMIXTOOLS2 v2.0.6 (*75*). All functions were run by providing the prefix of the genotype file. We calculated outgroup-*f*3 and *f*4 statistics using Mbuti, a Central African population as an outgroup to measure shared drift between worldwide populations and ancient Koreans or within ancient Koreans of interest. To explicitly model ancestry proportions of ancient Medieval East Asian populations, we used the function qpadm. We used the following set of populations as a right group: Central African hunter-gatherers Mbuti (Mbuti.DG, *n* = 5), Western European Hunter Gatherers (WHG, *n* = 10), Indigenous Andamanese islanders (Onge.DG, *n* = 2), Late Pleistocene Native American individual from the Upward Sun River site in Alaska (USR1, *n* = 1), 10,000 year-old ancient Paleo-Siberian from northeast Siberia (Kolyma_M, *n* = 1), early Neolithic Iranians from the Ganj Dareh site (Iran_N, *n* = 8), Early Neolithic farmers from western Anatolia (Anatolia_N, *n* = 23), Early to Middle Bronze Age Tarim Basin mummies (Tarim_EMBA1, *n* = 12), Early Neolithic hunter-gatherers from the west Baikal Region (Baikal_EN, *n* = 18), Early Neolithic Shandong ancients from China (Shandong_EN, *n* = 3), Early Neolithic individuals from the southern Chinese Liangdao site (Liangdao, *n* = 2), and Jomons from the Funadomari site in Japan (Jomon_Funadomari, *n* = 2).

## Supplementary Materials

### The PDF file includes:

Supplementary Text S1 and S2 Figs. S1 to S11 Legends for data S1 to S10 References
