# Supplementary material for: Ancient genomes reveal an extensive kinship network and endogamy in a Three-Kingdoms period society in Korea
Source: Sci Adv. 2026 Apr 8;12(15):eady8614. doi: 10.1126/sciadv.ady8614 (PMC13060608; doi:10.1126/sciadv.ady8614)
Supplement: Supplementary file 1 — Supplementary Text S1 and S2 Figs. S1 to S11 Legends for data S1 to S10 References [file sciadv.ady8614_sm.pdf]

Science Advances
AAAS

Supplementary Materials for

Ancient genomes reveal an extensive kinship network and endogamy in a Three-Kingdoms period society in Korea

Hyoungmin Moon *et al.*

Corresponding author: Daewook Kim, mozart0107@hanmail.net; Johannes Krause, krause@eva.mpg.de; Eun Jin Woo, redqin@sejong.ac.kr; Choongwon Jeong, cwjeong@snu.ac.kr

Sci. Adv. **12**, eady8614 (2026)
DOI: 10.1126/sciadv.ady8614

The PDF file includes:

Supplementary Text S1 and S2
Figs. S1 to S11
Legends for data S1 to S10
References

Other Supplementary Material for this manuscript includes the following:

Data S1 to S10

Supplementary Text 1. Archeological Description

S1.1 Provenance

Excavations were conducted in 1982, 1988, and 1989 by the Yeungnam University Museum. The principal investigator of the project was Young Wha Chung (at that time, Director of Yeungnam University Museum). The burial site from which the human remains were excavated are from the Imdang-Joyeong burial site (Gyeongsan, Republic of Korea). The time period of the burial sites was validated by both relative dating based on construction methods and excavated artifacts such as pottery within the tomb. All human remains are located at the Yeungnam University Museum in Gyeongsan, Republic of Korea, under the supervision of Dr. Daewook Kim.

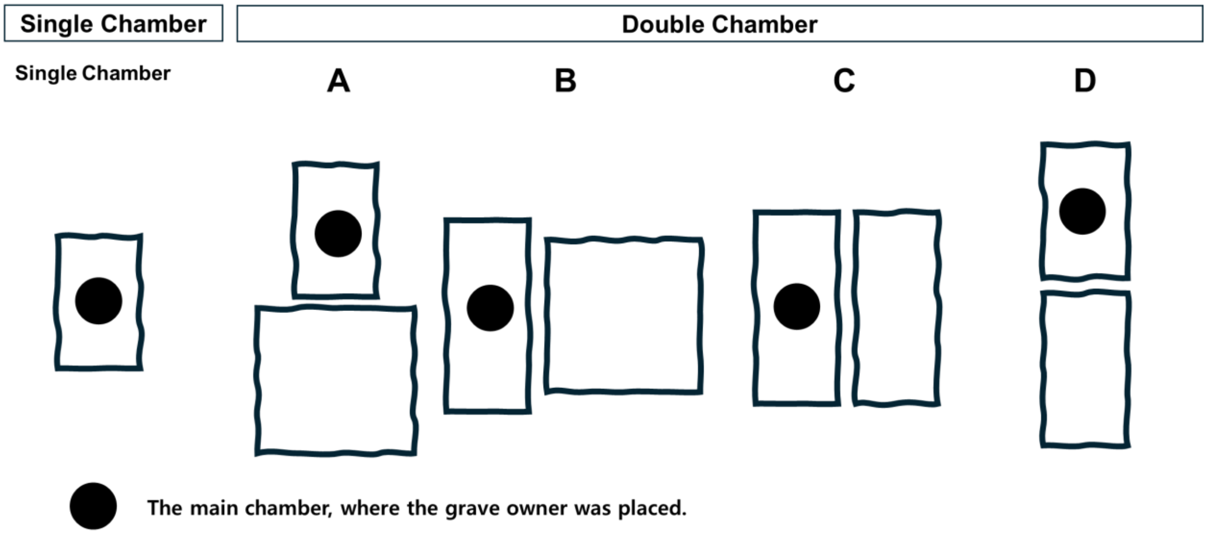

Figure S1.1.1. Burial types found in the Imdang-Joyoung burial complex and investigated in this study. Burial types are first divided into the single or double chamber burials depending on the number of chambers in each burial. Double chamber burials are further separated into the four types (types A to D) depending on the shape of the subsidiary chamber and its position relative to the main chamber.

S1.2 Archaeological Site and Sample information

Here we provide a summary of the archaeological backgrounds for each burial and ancient individuals found therein. For each burial, we describe the type, dating information, and individuals found therein, together with a schematic figure of the burial. In the table, we provide individual-level information of those whom we retrieved the genome-scale data in each burial.

- Imdang #2 South Tomb

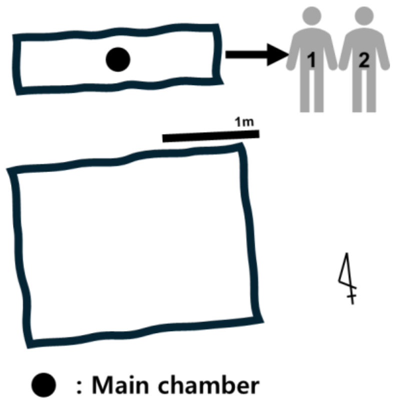

Imdang #2 South Tomb is a wooden chamber tomb of type B. The tomb was dated to the last quarter of the 5th century CE based on archaeological contexts. Both the main and subsidiary chambers were disturbed by grave robbery. Bone fragments from two individuals were discovered at the bottom of the main chamber, and the ownership of the grave was ambiguous. The subsidiary chamber was destroyed; no skeletal elements were recovered from it.

Archaeological ID	Sample ID	Age	Sex	Chamber	Status
001	IMD019	31 – 40	F	Main	Unknown
002	IMD020	adult	F	Main	Unknown

- Imdang #2 North Tomb

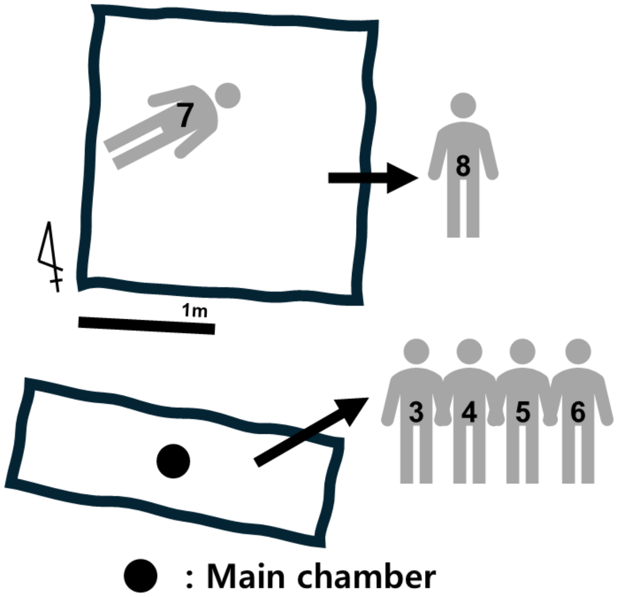

Imdang #2 North Tomb is a wooden chamber of type B. The tomb was constructed shortly after the construction of Imdang #2 South Tomb and was contextually dated to the last quarter of the 5th century CE based on archeological evidence. The main chamber was disturbed by grave robbery. A total of six individuals were found in the Imdang #2 North Tomb, four and two from the main and subsidiary chambers, respectively. The ownership of the tomb was ambiguous. All individuals from the subsidiary chamber were classified as sacrificed. From the subsidiary chamber, one

individual (007, IMD023) was found at the northwest corner of the chamber with their head placed towards the northeast, with the skeletons disturbed due to the collapse of the chamber. The individual was bent into an S-shape, with the upper body from the head to the chest and both legs curved. The individual was wearing a gilt-bronze thin round earring. Another individual (008) is thought to be placed near 007 or on top of the wooden coffin, based on the small space available within the subsidiary chamber.

Archaeological ID	Sample ID	Age	Sex	Chamber	Status
005	IMD001	21 – 35	M	Main	Unknown
003	IMD021	36 – 50	F	Main	Unknown
004	IMD022	21 – 35	M	Main	Unknown
007	IMD023	30	M	Subsidiary	Sacrificed

- Imdang 5A

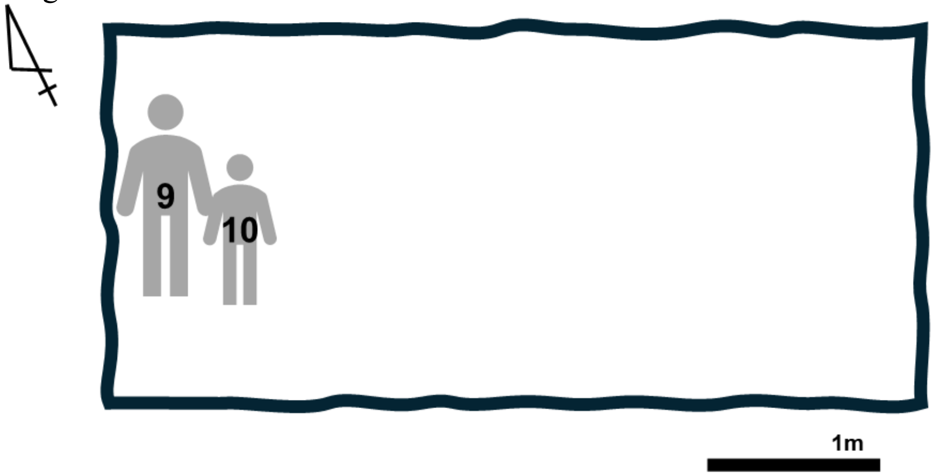

Imdang 5A is a lateral-entry single chamber tomb made of stone. The tomb was constructed during the last quarter of the 5th century CE based on archeological evidence. The tomb likely had a flat ceiling based on the shape of the stones forming the walls and the stones found at the bottom of the chamber. The chamber was disturbed by grave robbery. A total of two individuals were discovered from the chamber between the burial platform and the west wall, and both individuals were classified as sacrificed. While the grave owner was not discovered, it is likely that the grave owner was placed above the burial platform with their head placed towards the east, while the two sacrificed were placed between the pedestal and the west wall with their heads placed towards the north.

Archaeological ID	Sample ID	Age	Sex	Chamber	Status
010	IMD024	3	M	Room	Sacrificed

- Imdang 5B1

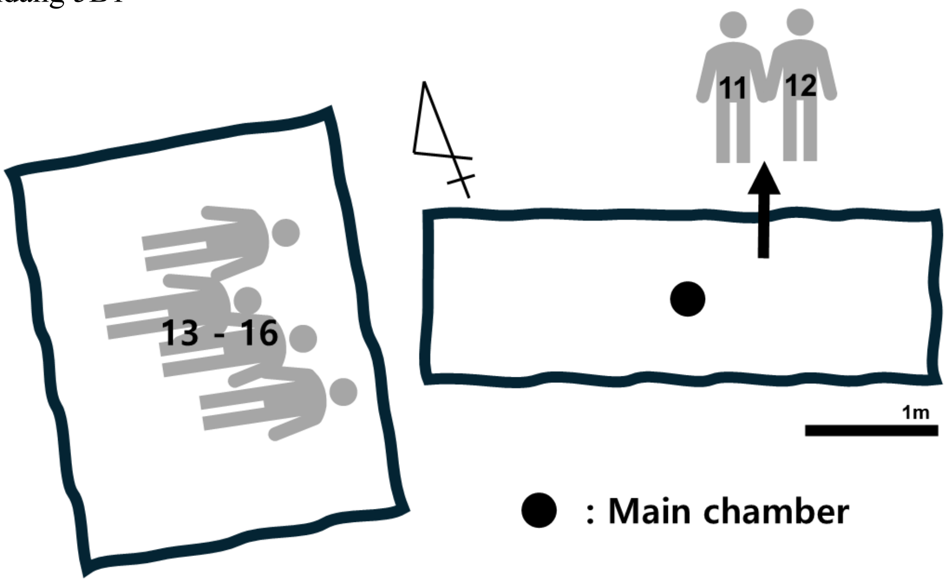

Imdang 5B1 is a wooden chamber tomb of type A. The tomb was dated during the last quarter of the 5th century CE based on archaeological context. The main chamber was disturbed by grave robbery. The main chamber contained a rich set of grave goods including ornaments such as a gilt-bronze crown, necklace with curved jade pendants, tube-shaped jade, and a silver ring, along with multiple pottery with ironware. The subsidiary chamber was constructed by vertical excavation with a wooden chamber placed inside. The subsidiary chamber contained an assortment of pottery placed near the chamber walls. Smaller pottery was placed in between the larger pottery. The pottery contained bones from livestock and shell remains. The chamber also contained exquisite saddlery, horse equipment, farming and working tools. A total of five individuals were found in the Imdang 5B1 tomb, one and four from the main and subsidiary chambers, respectively. From the main chamber, initially bones from two individuals were discovered, making the burial status ambiguous; however, both individuals turned out to be the same individual based on the analysis of this study. From the subsidiary chamber, all four individuals were labelled as sacrificed. Two were placed towards the southern wall while the rest were placed towards the western and northern wall each. The remains were scattered, likely due to the collapse of the wooden chamber, although their heads were likely positioned towards the east based on the position of the legs.

Archaeological ID	Sample ID	Age	Sex	Chamber	Status
011	IMD003	36 - 50	F	Main	Unknown
012	IMD003	21 - 35	F	Main	Unknown
013	IMD004	15 – 18	M	Subsidiary	Sacrificed
014	IMD005	21 – 35	M	Subsidiary	Sacrificed
015	IMD006	21 – 35	M	Subsidiary	Sacrificed
016	IMD007	36 - 50	F	Subsidiary	Sacrificed

- Imdang 5D1

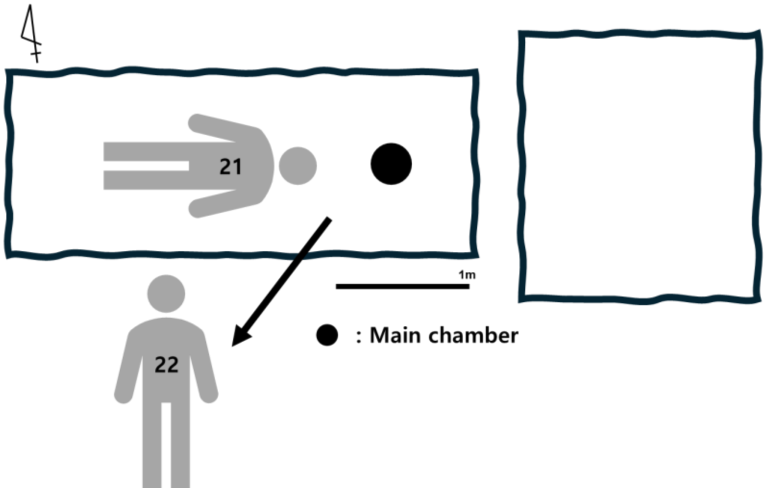

Imdang 5D1 is a wooden chamber of type A. The tomb was dated to the the second quarter of the 5th century CE. Two individuals were discovered within the main chamber. The grave owner was buried with their head oriented towards the east, surrounded by ornaments.

Archaeological ID	Sample ID	Age	Sex	Chamber	Status
021	IMD008	21 - 35	F	Main	Grave Owner

- Imdang 5D2

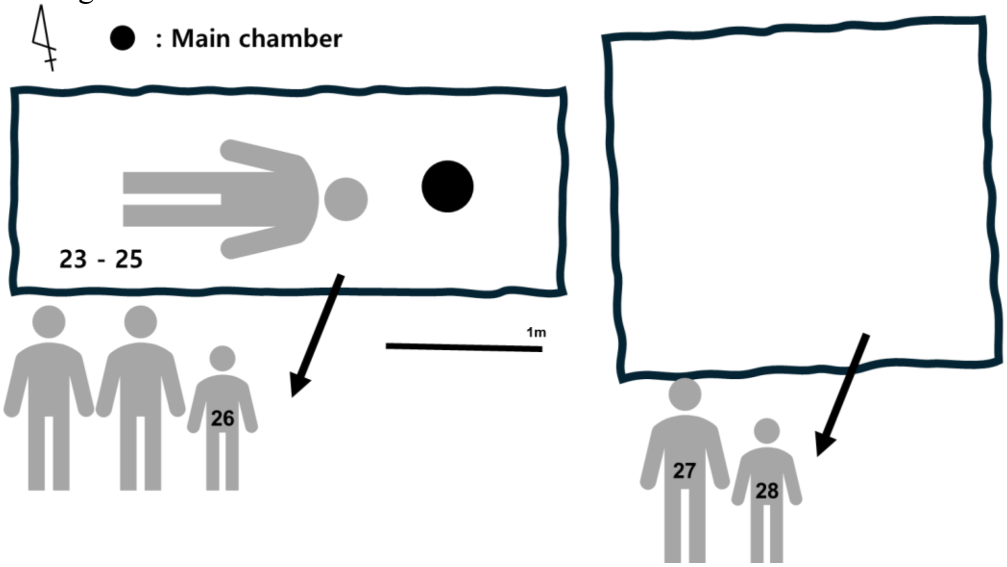

Imdang 5D2 is a wooden chamber of type A. The tomb was dated to the last quarter of the 5th century CE based on archaeological context. A total of six individuals were discovered, four and two individuals from the main and subsidiary chambers, respectively. The ownership of the grave was ambiguous, and the two individuals from the subsidiary chamber were classified as sacrificed. One individual from the main chamber was initially reported to have been buried with ornaments placed around their head and feet, while the head was placed towards the east; however, it is not clear who this individual was among the four individuals excavated from the main chamber.

Archaeological ID	Sample ID	Age	Sex	Chamber	Status
024	IMD009	36 - 50	M	Main	Unknown
027	IMD010	Adult	F	Subsidiary	Sacrificed
028	IMD027	4 - 8	F	Subsidiary	Sacrificed

- Imdang 6A

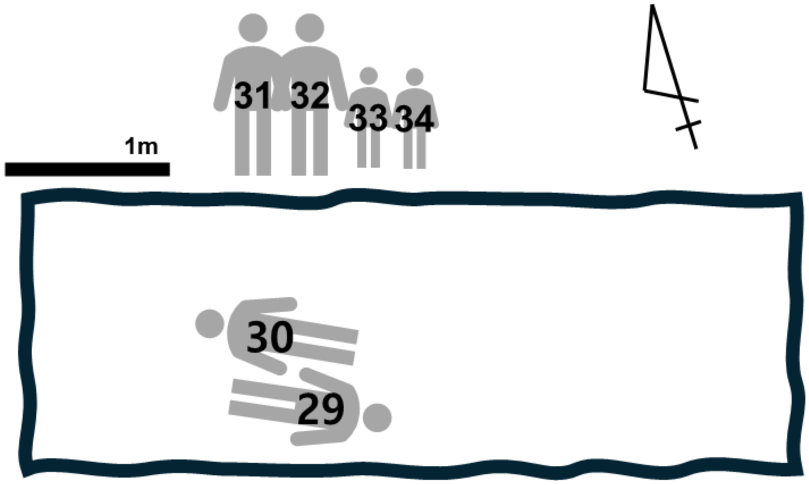

Imdang 6A is a tomb with a single wooden main chamber with no subsidiary chamber. The tomb was dated to the first quarter of the 6th century CE based on archaeological context. The chamber was disturbed by grave robbery. Ornaments such as a gilt-bronze crown, golden round and thin earrings, necklace with curved jade pendants, various pottery, shellfish remains, and fish bones were discovered. A total of six individuals were discovered within the chamber. The grave owner (029; IMD028), whose lower limbs were discovered under the fallen capstone of the tomb had a silver waist belt above the lower limb bone with a gilt-bronze sword with ring pommel to the left and gilt-bronze shoes near the feet. The head was likely positioned towards the east. One sacrificed individual (030; IMD029), placed towards the right of the grave owner had her crania well preserved with her arm and lower limbs discovered towards the east. The individual was likely positioned right of the grave owner's feet, with the head placed towards the south. The position of the remaining four individuals was ambiguous. Considering that the chamber was full of burial goods with not enough space, some of the sacrificed individuals may have been placed above the wooden coffin.

Archaeological ID	Sample ID	Age	Sex	Chamber	Status
029	IMD028	21 – 35	M	Inside	Owner
030	IMD029	21 – 35	F	Inside	Sacrificed
032	IMD031	41- 60	M	Inside	Sacrificed
034	IMD011	4 - 8	F	Inside	Sacrificed

- Imdang 6B1

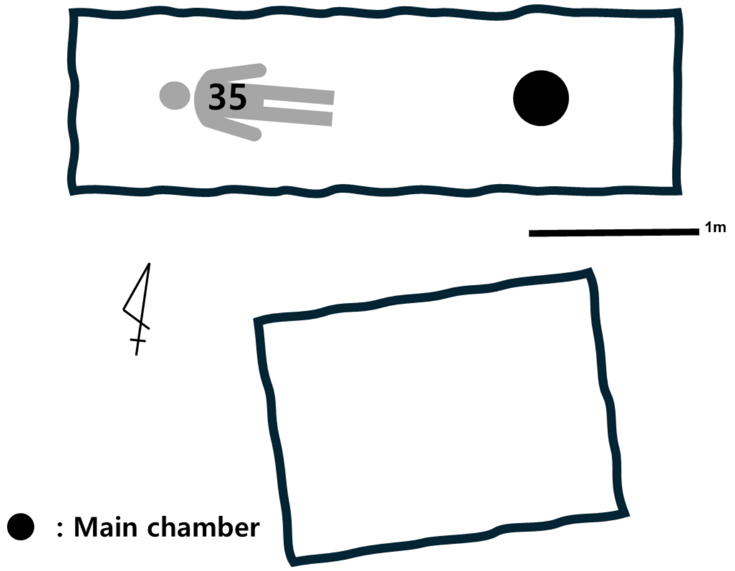

Imdang 6B1 is a wooden chamber tomb of type B. The tomb was dated to the first quarter of the 6th century CE based on archeological evidence. The main chamber was disturbed by grave robbery. A single individual was discovered within the main chamber. The grave owner was situated within a wooden coffin in the middle of the chamber, with the head pointing eastward. Only parts of the lower limbs were recoverable, likely due to the disturbance caused by grave robbery.

Archaeological ID	Sample ID	Age	Sex	Chamber	Status
035	IMD033	36 – 50	U	Main	Owner

- Imdang 7B

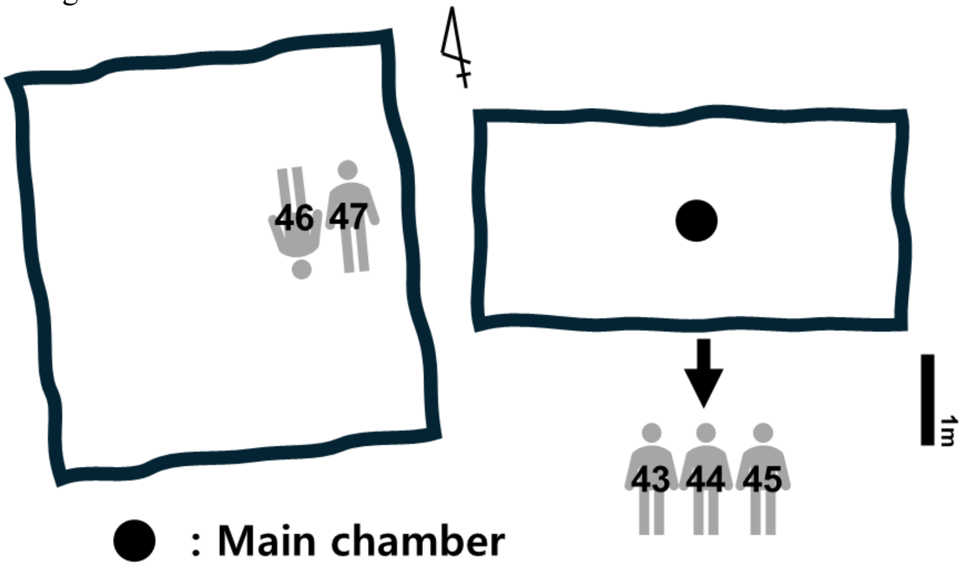

Imdang 7B is a wooden chamber tomb of type A. The tomb was dated to the second quarter of the 5th century CE based on archaeological evidence. The main and subsidiary chambers contained a wooden coffin installed at the center. The main chamber was disturbed by grave robbery. Large

pots were lined up towards the southern wall, with soft earthenware buried around the sides. Fish bones, animal bones, and seashells were contained inside the pottery. An assortment of weaponry and horse equipment was found between and above the pottery, along with iron arrowheads and spearheads. Multiple shark vertebra was found at the center of the tomb. A total of five individuals were found in the Imdang 7B tomb, three and two from the main and subsidiary chambers, respectively. Among the three individuals from the main chamber, one individual had their head pointed towards the east with a gilt-bronze crown with ornaments, silver decorated gilt-bronze waist belt, glass-bead necklace, and iron sword. The cranial and chest bones of another individual were found west of the center under the covering stones, but the exact assignment of the skeletal elements were ambiguous, leaving the ownership of the grave uncertain. All skeletons discovered from the subsidiary tomb were discovered from the northeast side of the chamber. While the skeletons were heavily degraded, making it difficult to discern exact posture, the skeleton towards the center (046) had their head towards the south, while the skeleton towards the east had their head towards the north (047).

Archaeological ID	Sample ID	Age	Sex	Chamber	Status
043	IMD016	8.5 – 13.5	F	Main	Unknown

- Imdang 7C

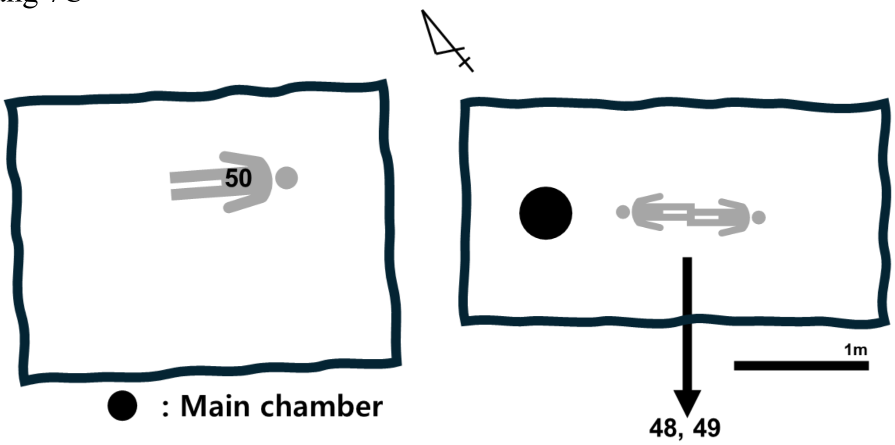

Imdang 7C is a wooden chamber tomb of type A. The tomb was dated to the second quarter of the 5th century CE based on archaeological evidence. The floor of the main chamber was levelled to place a wooden coffin inside. The main chamber contained a plethora of goods, including a gilt-bronze crown, golden fat earrings, silver rings, necklace with curved jade pendants and curved jade. Towards the east of the main chamber, where the head of the grave owner likely laid, a partition was set to bury various pottery. Towards the southwest of the main chamber, thin earrings and glass beads were discovered, likely from a sacrificed individual. A total of three individuals were discovered from the tomb, two and one from the main and subsidiary chambers, respectively. The ownership of the tomb was ambiguous. The individual from the subsidiary chamber was buried together with multiple horse equipment and pottery.

Archaeological ID	Sample ID	Age	Sex	Chamber	Status
048	IMD037	2 – 4	F	Main	Unknown
050	IMD039	21 - 35	F	Subsidiary	Sacrificed

- Imdang 7D

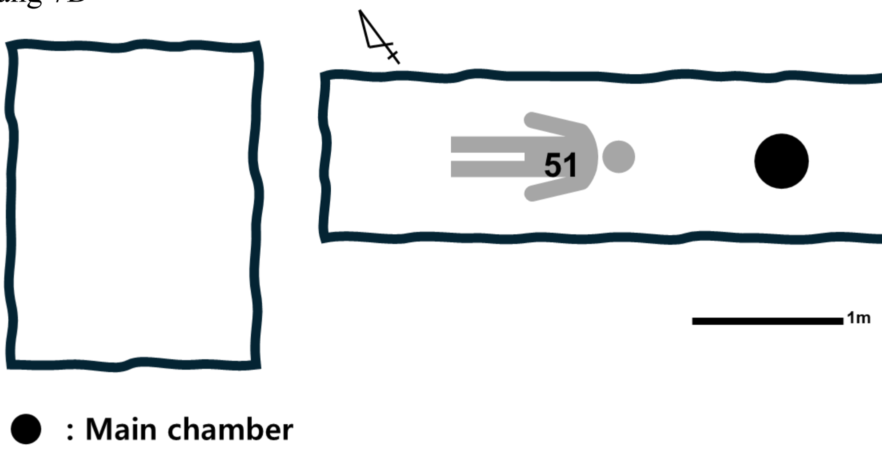

Imdang 7D is a tomb of type A. The tomb was dated to the last quarter of the 5th century CE. Only one individual was discovered in the main chamber within a wooden coffin. The head of the grave owner was placed towards the southeast, accompanied by various ornaments. The skeleton was well preserved with a few disturbed elements.

Archaeological ID	Sample ID	Age	Sex	Chamber	Status
051	IMD018	21 – 35	F	Main	Owner

- Joyeong 1A-2

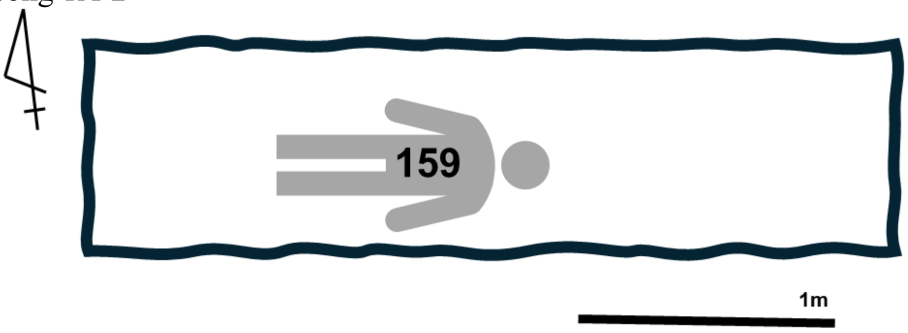

Joyeong 1A-2 is a tomb with a single wooden chamber. The tomb was dated to the first quarter of the 6th century CE. Only one individual was discovered within the chamber, who was the grave owner. The grave owner was likely straightened before burial, although several bones were disoriented from their original position and were heavily decayed. The ribs were found at the easternmost side, with the pelvic girdle, cranial, humerus, radius, ulna, tibia, fibula, and femur towards the west. The disturbance of the position of the bones is likely due to the collapse of the cover of the dirt mound of the tomb, rather than by human disturbance.

Archaeological ID	Sample ID	Age	Sex	Chamber	Status
159	JOY076	3.5 – 6.5	F	Inside	Owner

- Joyeong 1A-3

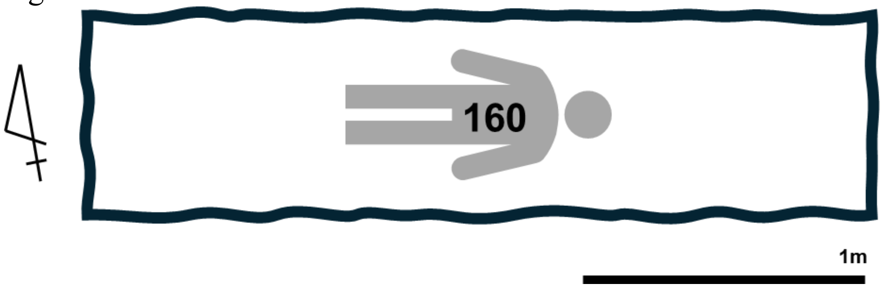

Joyeong 1A-3 is a tomb with a single wooden chamber. The tomb was dated to the third quarter of the 5th century. The tomb was destroyed prior to excavation. Only one individual was discovered and was the owner of the grave. They were located across the center and western part of the chamber, with their head pointing towards the east with their body stretched out. However, due to the destruction of the tomb, only the arms, ribs, fragments of the left femur, right tibia, and fibula were intact. Some parts of the cranial and femur were recovered from the soil during excavation.

Archaeological ID	Sample ID	Age	Sex	Chamber	Status
160	JOY077	21 – 35	M	Inside	Owner

- Joyeong 1A-4

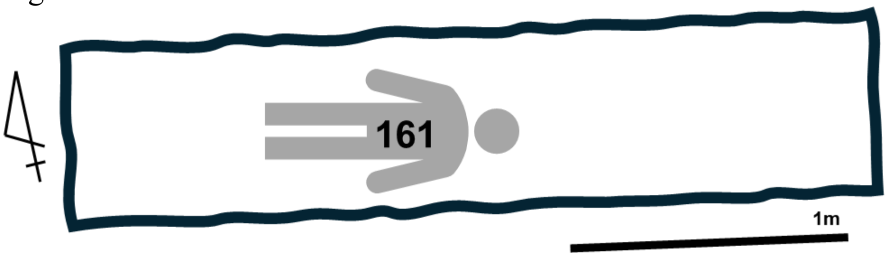

Joyeong 1A-4 is a tomb with a single wooden chamber. The tomb was dated to the third quarter of the 5th century based on archaeological evidence. The eastern part of the tomb served as a space for funerary goods. Only one individual was discovered from the tomb and was the grave owner. They had their head placed towards the east, with most of the skeletal remains decayed, leaving only parts of the crania and limbs in place. Other parts of the crania and lower jawbone were recovered right under the southern side of the tomb, under the remains of a fallen wall. Decayed radius, femur, tibia, and fibula were scattered across the tomb.

Archaeological ID	Sample ID	Age	Sex	Chamber	Status
161	JOY078	36 - 50	F	Inside	Owner

- Joyeong 1A-6

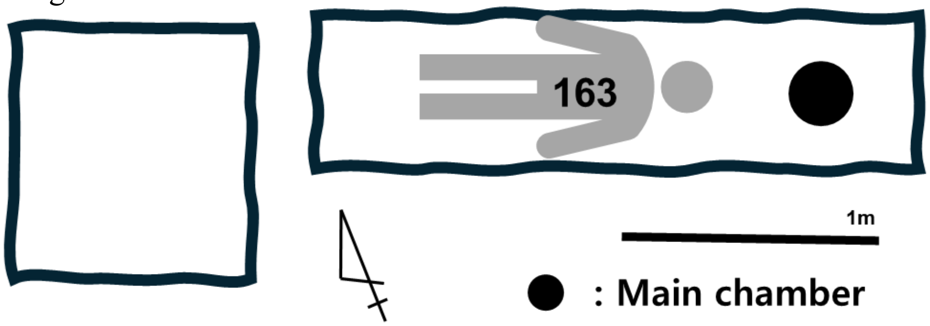

Joyeong 1A-6 is a double chamber tomb of type A. The tomb was dated to the last quarter of the 5th century CE based on archaeological evidence. Only one individual was discovered from the tomb and was the grave owner. The body of the owner was intact, with only the cranial shifted slightly towards the north. The head was placed towards the east with the body stretched out. Bones from a fetus were discovered alongside the grave owner.

Archaeological ID	Sample ID	Age	Sex	Chamber	Status
163	JOY028	21 - 35	F	Inside	Owner

- Joyeong 1A-10

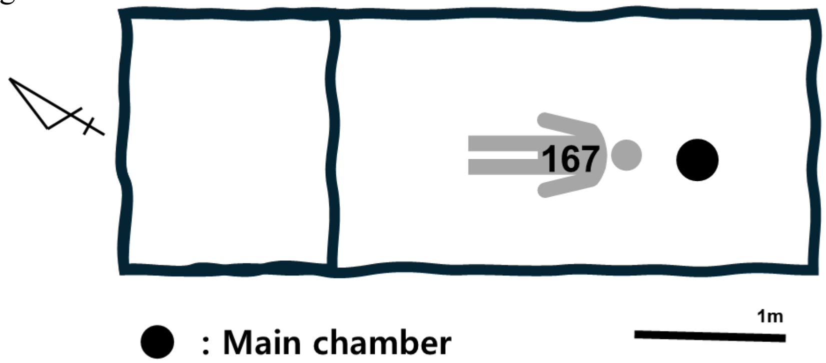

Joyeong 1A-10 is a wooden chamber tomb of type D. The tomb was dated to the last quarter of the 5th century CE based on archaeological evidence. Only one individual was discovered from the tomb and was the grave owner. The grave owner was placed with their head placed towards the southeast and body stretched out. The bones have undergone serious decay and were in bad condition.

Archaeological ID	Sample ID	Age	Sex	Chamber	Status
167	JOY083	21 - 35	Undetermined	Inside	Owner

- Joyeong 1A-11

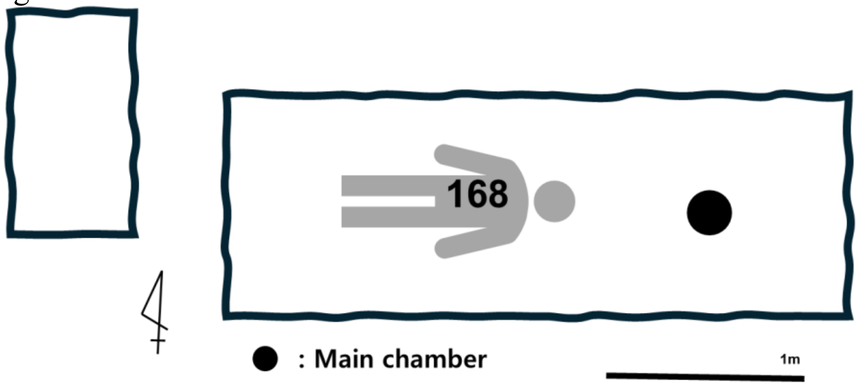

Joyeong 1A-11 is a wooden tomb of type A. The tomb was dated to the first quarter of the 6th century CE based on archaeological evidence. Only one individual was discovered from the tomb and was the grave owner. The bones were heavily decayed with the body stretching across the center and west of the tomb, head pointing eastward. The cranial was heavily damaged and shifted towards the northwest, upper body was slightly disturbed, while the lower body remained intact.

Archaeological ID	Sample ID	Age	Sex	Chamber	Status
168	JOY084	36 - 50	F	Inside	Owner

- Joyeong 1A-19

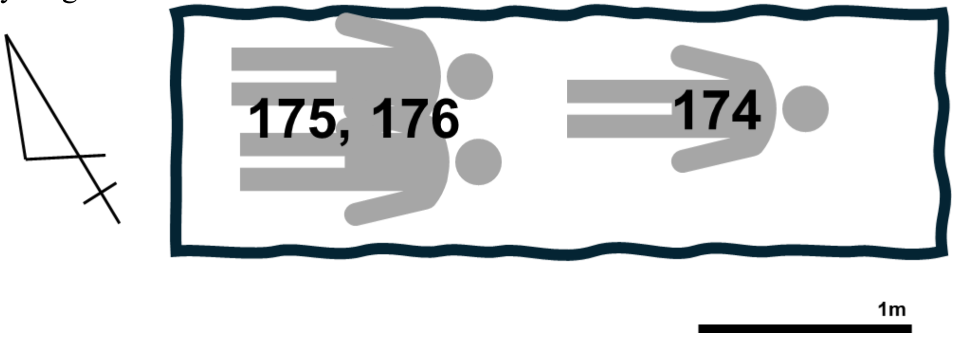

Joyeong 1A-19 is a single wooden chamber tomb of considerable size. The tomb was dated between the end of the 3rd century CE and start of the 4th century CE. A wall likely separated the middle of the tomb into a main chamber-like compartment and subsidiary chamber-like compartment, similar to a type D tomb. A total of three individuals were found within the tomb. A total of three individuals were discovered within the chamber. While all individuals had their head towards the southeast with their bodies stretched out, the individual towards the southeast (174) was decided as the grave owner based on the abundance of ornaments surrounding the individual, along with the southeast position being of higher importance compared to the northwest. The remaining two individuals were classified as sacrificed. The bones of the grave owner have undergone heavy decay with only parts of the crania and lower body remaining. The two sacrificed individuals were in relatively better condition.

Archaeological ID	Sample ID	Age	Sex	Chamber	Status
176	JOY091	21 - 35	F	main	Sacrificed

- Joyeong 1B-39

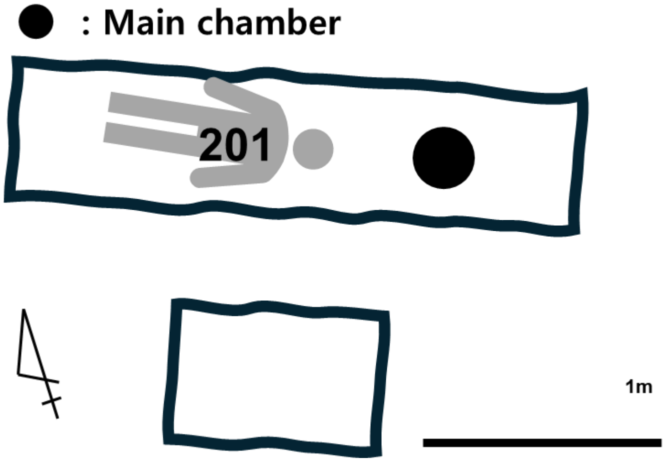

Joyeong 1B-39 is a wooden chamber tomb of type B. The tomb was dated to the first quarter of the 6th century CE. Only one individual was found from the tomb, inside the main chamber. The skeletal remains were abundant but were heavily disrupted. The humerus, radius, and ulna were scattered across the southern long wall of the tomb. The pelvic girdle and femur were located at the western center of the tomb relatively unscathed, while the tibia and fibula were clustered around the west short side of the tomb wall. Based on the position of the bones, the grave owner was likely placed stretched out with their head towards the southeast.

Archaeological ID	Sample ID	Age	Sex	Chamber	Status
201	JOY106	21 - 35	F	main	Owner

- Joyeong 1B-40

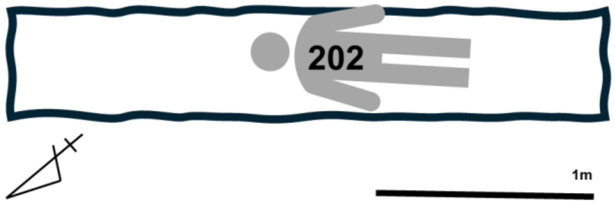

Joyeong 1B-40 is a tomb with a single wooden chamber. The tomb was dated to the last quarter of the 5th century CE, based on archaeological evidence. Only one individual was discovered, with their head placing northeastward with the body laid out in a supine position. The skeletal remains were extended across the central and northern parts of the tomb, with the right os coxa and both tibiae and fibulae displaced from their original position, though they were relatively neatly exposed.

Archaeological ID	Sample ID	Age	Sex	Chamber	Status
202	JOY107	31 - 40	M	main	Owner

- Joyeong 3B-1

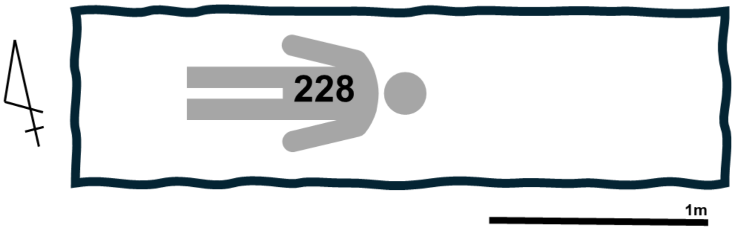

Joyeong 3B-1 is a tomb with a single wooden chamber. The tomb was dated to the middle of the 6th century based on archaeological evidence. Only one individual was discovered, with their body in a supine position, head oriented towards the east. The bones have undergone heavy decay.

Archaeological ID	Sample ID	Age	Sex	Chamber	Status
228	JOY118	21 - 35	F	main	Owner

- Joyeong 3B-2

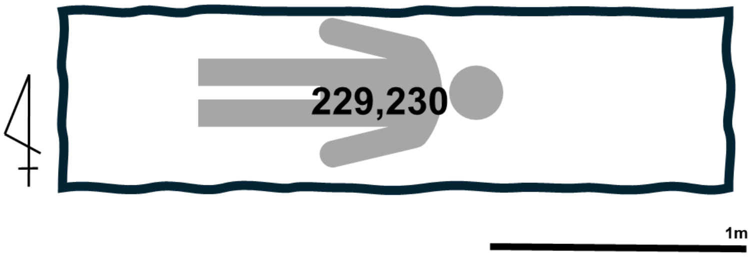

Joyeong 3B-2 is a tomb with a single wooden chamber. The tomb was dated to the 6th century CE based on archaeological evidence. Only one individual was discovered within the chamber. Initially, two individuals were reported to be found within the chamber, making the burial status ambiguous. However, the two bones studied in this study turned out to be from the same individual. The crania were found near the short side of the tomb wall, and the maxilla and mandible were found 150 cm away from the crania near the eastern short wall. The rest of the skeleton, including the humerus, ulna, scapula, femur, tibia, and fibula were intact.

Archaeological ID	Sample ID	Age	Sex	Chamber	Status
229	JOY119	41 – 60	F	Inside	Unknown
230	JOY119	21 - 35	F	Inside	Unknown

- Joyeong 3B-3

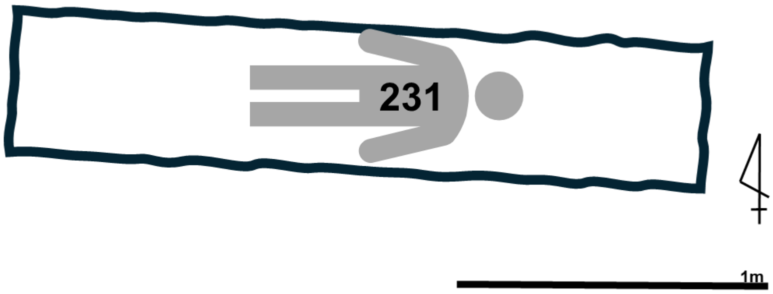

Joyeong 3B-3 is a tomb with a single wooden chamber. The tomb was dated to the 6th century CE based on archaeological evidence. Only one individual was discovered within the tomb. The grave owner was in a supine position with the head oriented towards the east. The bones were not disturbed and were well preserved.

Archaeological ID	Sample ID	Age	Sex	Chamber	Status
231	JOY121	31 - 40	F	Inside	Owner

- Joyeong 3B-8

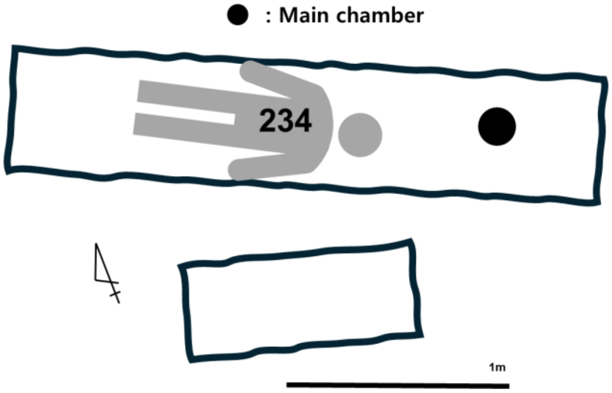

Joyeong 3B-8 is a double chamber tomb of type B. The tomb was dated to the middle of the 6th century CE based on archaeological evidence. Only one individual was discovered from the tomb and was considered as the owner of the grave. The grave owner was in a supine position with the head towards the east, while the bones were heavily decayed.

Archaeological ID	Sample ID	Age	Sex	Chamber	Status
234	JOY123	41 - 60	M	Main	Owner

- Joyeong CI-1

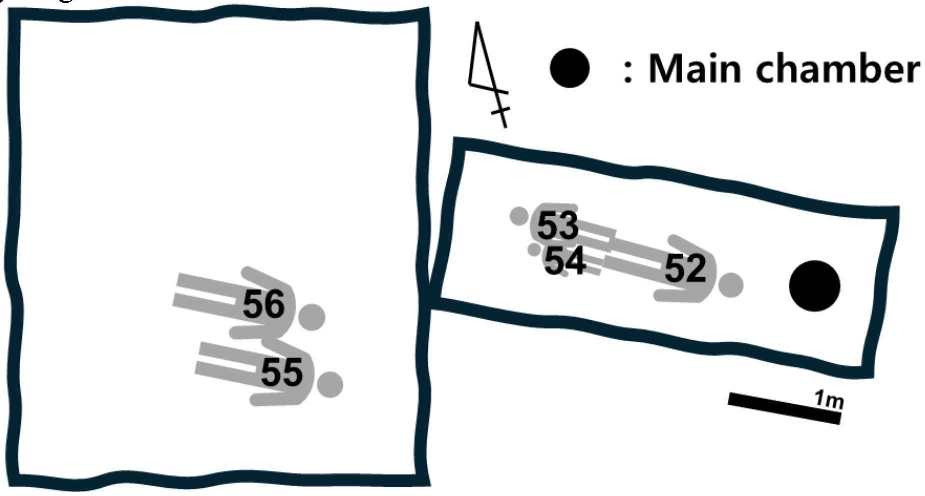

Joyeong CI-1 is a wooden chamber tomb of type A. The tomb was dated to the third quarter of the 6th century CE based on archaeological evidence. A total of five individuals were discovered from the tomb, with three and two individuals from the main and subsidiary chambers, respectively. The individual at the center of the main chamber was decided as the owner of the grave based on ornaments and the position of the head oriented towards the east. The cranial, left humerus, pelvic girdle, femur, tibia, and foot bones were not disturbed. The other two individuals from the main chamber were sacrificed individuals with their heads pointing towards the west. The crania, right humerus, ribs, right femur of one sacrificed individual was discovered towards the north wall of the tomb. The rib bones were disturbed and clustered towards the femur, likely from the result of a forced opening of a grave robbery. The cranial and femur of the second sacrificed individual was discovered towards the south wall of the main chamber. All three individuals of the main chamber were in a supine position. The two individuals from the subsidiary chamber were decided as sacrificed ones. The two individuals were placed in parallel at the southeast side of the chamber with their heads placed towards the east, likely in a supine position. The individual towards the south (055) had his head closely positioned towards the east wall, body close and parallel against the south wall of the chamber. The bones of this individual were heavily decayed, with only the crania, mandible, humerus, radius, ulna, parts of the pelvic girdle, femur, left tibia and fibula remaining. Only the left femur was slightly disturbed. The bones of the second individual consisted of the crania, femur, left tibia, and fibula. Despite the disturbance due to the collapse of the wooden coffin, which scattered some of the bones, the two individuals were clearly positioned parallel to each other.

Archaeological ID	Sample ID	Age	Sex	Chamber	Status
52	JOY001	31-40	M	Main	Owner
53	JOY002	15-18	M	Main	Sacrificed
54	JOY003	4-8	F	Main	Sacrificed
55	JOY004	41-60	M	Subsidiary	Sacrificed
56	JOY005	36-50	F	Subsidiary	Sacrificed

- Joyeong CI-2

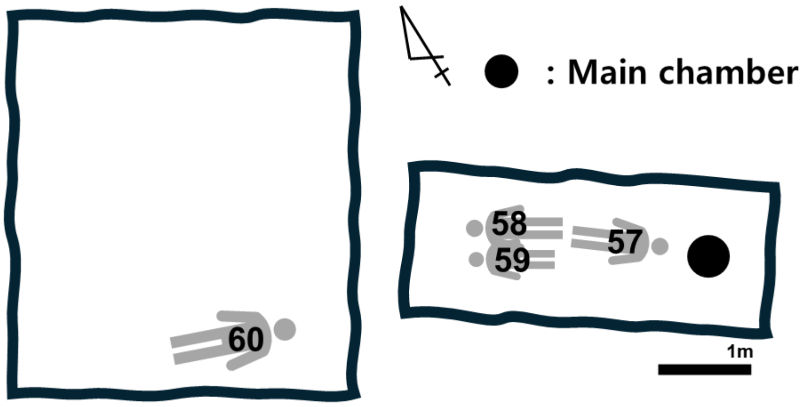

Joyeong CI-2 is a wooden chamber tomb of type A. The tomb was dated to the third quarter of the 6th century CE based on archaeological evidence. A total of four individuals were discovered from the tomb, with three and one from the main and subsidiary chambers, respectively. The crania of the grave owner (057) were discovered in place of the eastern center of the coffin, with a necklace with curved jade pendant and silver balls with a hole in the middle discovered at the position of where the chest should have been. This suggested that the grave owner was buried with their head pointing towards the east. The mandible of one sacrificed individual was discovered at the southwest corner of the main chamber, suggesting that their head was oriented towards the west. In our analysis, the teeth from JOY006 and JOY029 belonged to the same person. Based on the age estimate of the two teeth being closer to that of the sacrificed individual, the samples used in this analysis were classified as sacrificed.

The sacrificed individual from the subsidiary chamber was discovered with his head towards the east at the southeast position of the chamber. The body was heavily disturbed, due to the collapse of the chamber. Judging from the position of the bones, the sacrificed was placed in a supine position parallel to the south wall. One pair of gilt-bronze earrings were found near the crania of the sacrificed individual.

Archaeological ID	Sample ID	Age	Sex	Chamber	Status
57	JOY029 (JOY006)	36 – 50	F	Main	Owner
58	JOY006 (JOY029)	21 – 35	F	Main	Sacrificed
59	JOY030	21 – 35	F	Main	Sacrificed
60	JOY007	15 – 18	M	Subsidiary	Sacrificed

- Joyeong CI-4

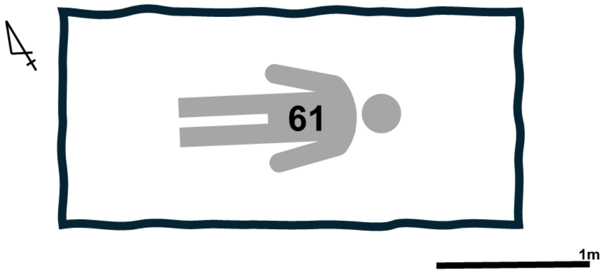

Joyeong CI-4 is a tomb with a single wooden chamber. The tomb was contextually dated between the end of the 3rd century CE and start of the 4th century CE based on archaeological evidence. Only one individual was found in the tomb, who was the grave owner. The individual was positioned at the middle of the chamber with their upper left limb and parts of the lower limbs remaining.

Archaeological ID	Sample ID	Age	Sex	Chamber	Status
61	JOY031	adult	M	Inside	Owner

- Joyeong CII-1

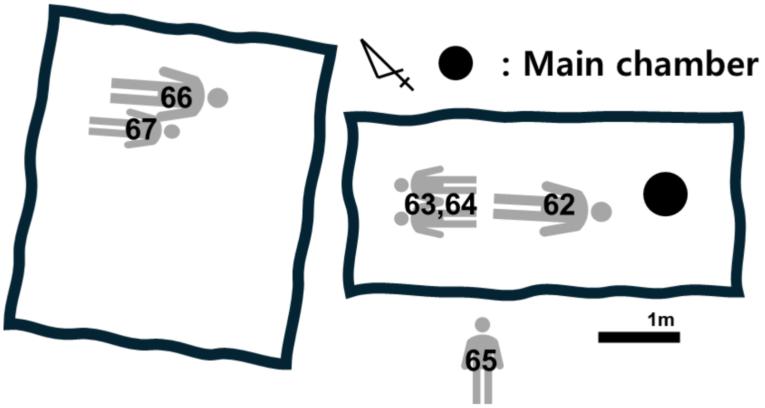

Joyeong CII-1 is a wooden chamber tomb of type A. The tomb was contextually dated to the first quarter of the 5th century CE based on archaeological evidence. A total of six individuals were excavated from the tomb, with four and two individuals from the main and subsidiary chamber, respectively. The owner of the grave was decided based on the ornaments they were wearing and was the individual at the middle of the main chamber. The grave owner was laid in a supine position against the southeast wall. Traces of the crania and both leg bones were exposed, undisturbed. The rest of the individuals were considered as sacrificed. The positions of the remaining individuals from the main chamber were disturbed due to the decay and collapse of the main chamber. While the exact positions of the three sacrificed individuals are not clear, two individuals were buried with their heads pointing the opposite direction of the grave owner's head, and one individual was likely buried on top of the wooden chamber. Two sacrificed individuals were found in the subsidiary chamber. The two individuals were positioned parallel to each other towards the northeast side of the chamber, heads oriented towards the southeast. The position of the bones was heavily disturbed due to the collapse of the subsidiary chamber.

Archaeological ID	Sample ID	Age	Sex	Chamber	Status
62	JOY032	36 – 50	F	Main	Owner
63	JOY008	16 – 18	F	Main	Sacrificed
64	JOY009	10	M	Main	Sacrificed
66	JOY010	36 – 50	M	Subsidiary	Sacrificed
67	JOY011	10	F	Subsidiary	Sacrificed

- Joyeong CII-2

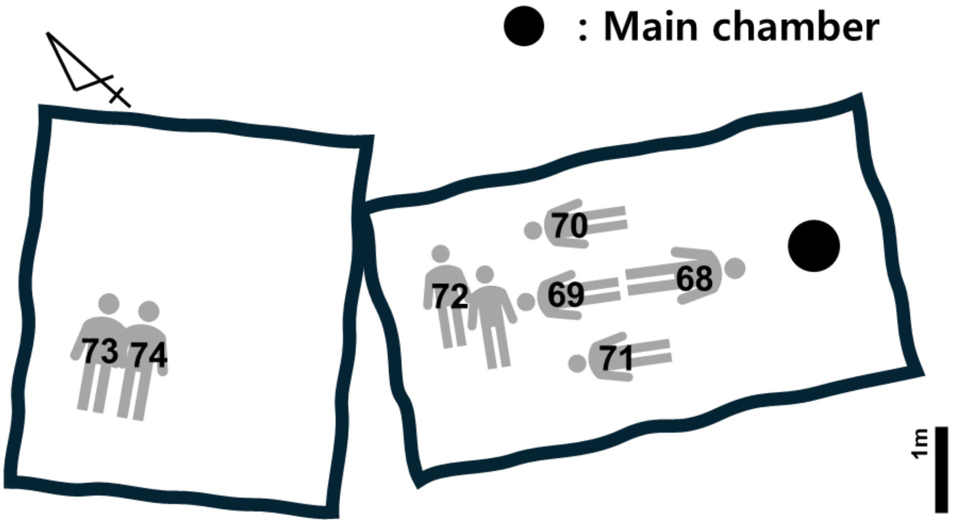

Joyeong CII-2 is a wooden chamber tomb of type A. The main chamber was constructed by digging a hole, constructing an outer and inner wall, then filling stones in between. The chamber floor was filled with stones to serve as a pedestal. The subsidiary chamber was positioned towards the northwest direction. The tomb was contextually dated to the first quarter of the 5th century CE based on archaeological evidence. A total of eight individuals were discovered from Joyeong CII-2, with six and two individuals from the main and subsidiary chamber, respectively. From the main chamber, the grave owner was identified. The other individuals were sacrificed and were each placed around the feet of the grave owner, along the south wall, and along the west wall of the main chamber. The two sacrificed individuals in the subsidiary chamber were placed along the northwest wall of the chamber with their heads placed towards the northeast in a supine position. The upper bones were scattered due to the collapse of the wooden chamber. The lower bones were relatively intact.

Archaeological ID	Sample ID	Age	Sex	Chamber	Status
068	JOY012	30	M	Main	Owner
069	JOY033	18 – 20	F	Main	Sacrificed
070	JOY034	10	F	Main	Sacrificed
072	JOY013	21 – 35	M	Main	Sacrificed
073	JOY035	21 – 35	F	Subsidiary	Sacrificed

- Joyeong EII-2

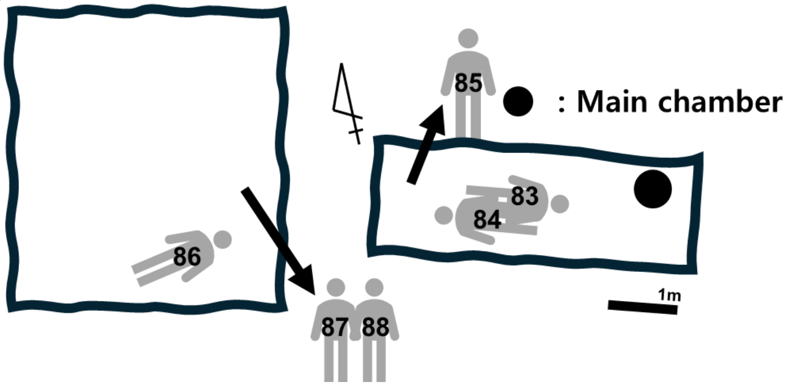

Joyeong EII-2 is a wooden chamber tomb of type A. The tomb was contextually dated to the last quarter of the 5th century CE based on archaeological evidence. A total of six individuals were discovered from Joyeong EII-2, three individuals each from the main and subsidiary chambers. The two sacrificed individuals from the main chamber had each of their heads pointing towards the west and east. The remaining individual from the main chamber was considered the grave owner. In the subsidiary chamber, the location of one individual was clearly identified, while the position of the other two individuals is unknown.

Archaeological ID	Sample ID	Age	Genetic Sex	Chamber	Status
083	JOY015	15 - 18	F	Main	Sacrificed
084	JOY016	7.5 – 12.5	F	Main	Sacrificed

- Joyeong EII-3

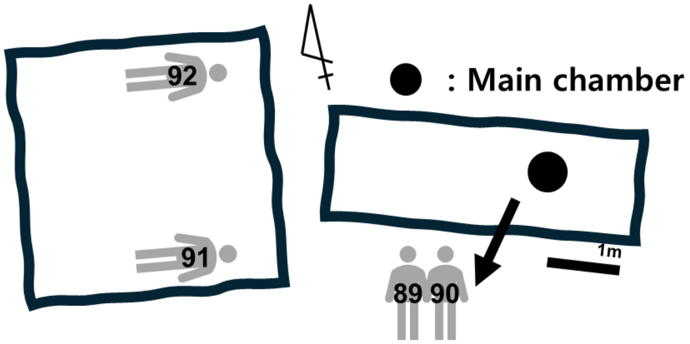

Joyeong EII-3 is a wooden chamber tomb of type A. The tomb was dated to the first quarter of the 6th century CE based on archaeological evidence. A total of four individuals were discovered from EII-3, two each from the main and subsidiary chamber. The ownership of the grave was ambiguous between the two individuals from the main chamber. The two sacrificed individuals from the subsidiary chamber were placed beside the northern and southern wall, respectively.

Archaeological ID	Sample ID	Age	Sex	Chamber	Status
089	JOY017	21 – 35	M	Main	Unknown
090	JOY018	20	M	Main	Unknown
091	JOY019	Adult	F	Subsidiary	Sacrificed
092	JOY020	21 – 35	M	Subsidiary	Sacrificed

- Joyeong EII-6

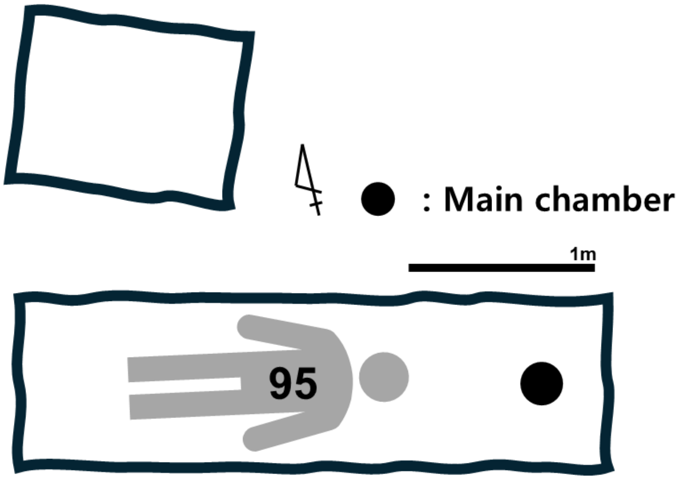

Joyeong EII-6 is a wooden chamber tomb of type B. The main chamber was placed towards the south, and the subsidiary chamber was placed towards the north. The tomb was contextually dated to the start of the 6th century CE based on archaeological evidence. One individual was discovered within the main chamber. While likely the grave owner, the exact position of this individual was uncertain, making their status ambiguous.

Archaeological ID	Sample ID	Age	Sex	Chamber	Status
095	JOY042	21 – 35	F	Main	Unknown

- Joyeong EII-7

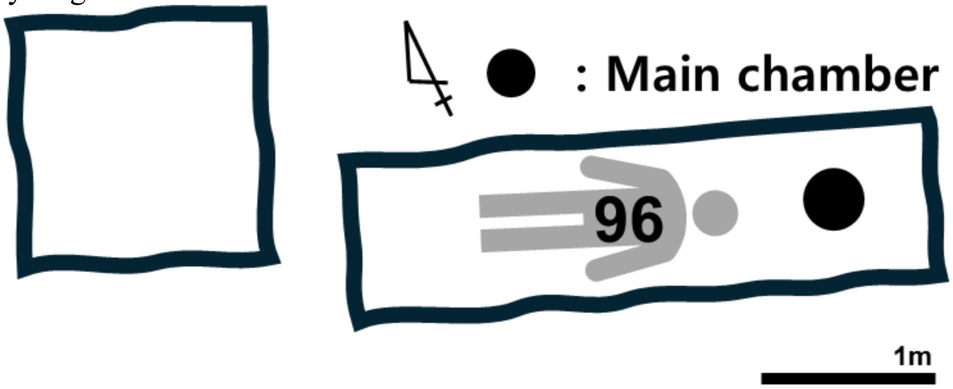

Joyeong EII-7 is a wooden chamber of type A. The main chamber was placed towards the east, and the subsidiary chamber was placed towards the west. The tomb was contextually dated to the start of the 6th century CE based on archaeological evidence. A bow was discovered towards the north wall of the chamber. One individual was discovered within the main chamber, with their head oriented towards the east. The grave owner was buried with knives around his left femur. Iron arrowheads and spearheads were placed above the grave owner's head.

Archaeological ID	Sample ID	Age	Sex	Chamber	Status
096	JOY021	21 – 40	M	Main	Owner

- Joyeong EII-13

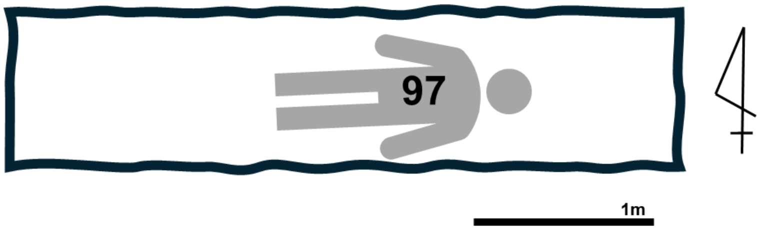

Joyeong EII-13 is a single wooden chamber, and most of it was heavily destroyed prior to excavation. The tomb was contextually dated to the start of the 6th century CE based on archaeological evidence. Only one individual was discovered, with his head oriented towards the east. A substantial amount of grave goods was found around the grave owner's lower legs and head.

Archaeological ID	Sample ID	Age	Sex	Chamber	Status
097	JOY022	21 – 35	M	Inside	Owner

- Joyeong EIII-2

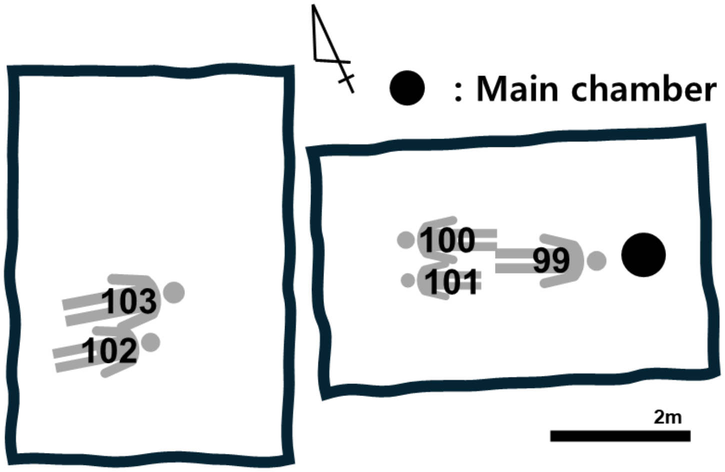

Joyeong EIII-2 is a stone chamber of type A. A total of five individuals were discovered from EIII-2, three and two individuals from the main and subsidiary chambers, respectively. The grave owner from the main chamber had his/her head placed towards the east, while the other two sacrificed individuals from the main chamber were placed around the foot of the grave owner.

Archaeological ID	Sample ID	Age	Sex	Chamber	Status
100	JOY023	21 – 35	F	Main	Sacrificed
101	JOY043	6 – 12	M	Main	Sacrificed
102	JOY024	21 – 35	F	Subsidiary	Sacrificed
103	JOY025	21 – 35	M	Subsidiary	Sacrificed

- Joyeong EIII-3

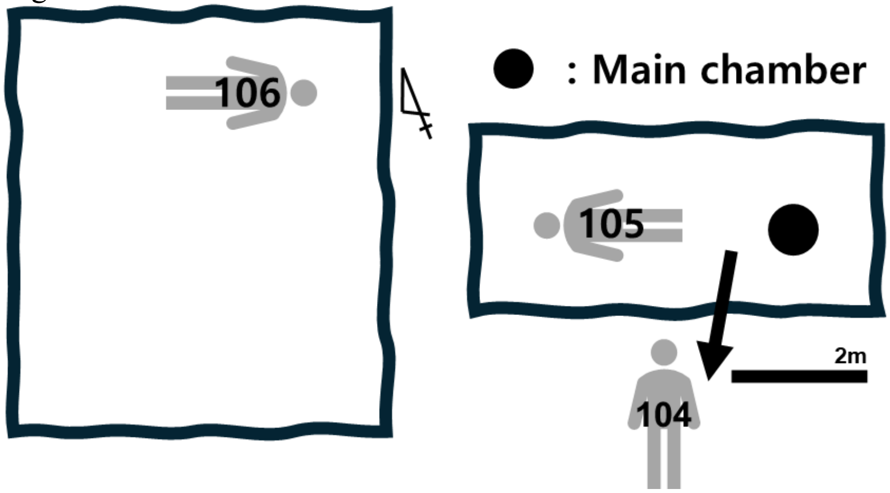

Joyeong EIII-3 is a wooden chamber of type A. The tomb was contextually dated to the second quarter of the 5th century CE based on archaeological evidence. Three individuals were discovered from Joyeong EIII-3, two and one from the main and subsidiary chambers, respectively. Due to the ambiguity of the position of the individual, the status of one individual from the main chamber was ambiguous. The other individual from the main chamber had their head towards the west and was classified as sacrificed. The individual in the subsidiary chamber had his head positioned towards the east, close to the north wall of the chamber. Arms and legs were not disturbed.

Archaeological ID	Sample ID	Age	Sex	Chamber	Status
106	JOY045	21 – 35	M	Subsidiary	Sacrificed

- Joyeong EIII-4

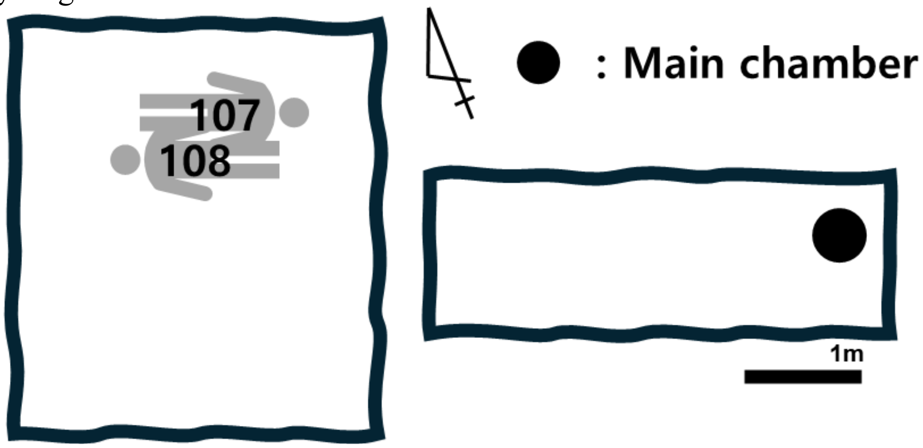

Joyeong EIII-4 is a wooden chamber of type A. The tomb was contextually dated to the second quarter of the 5th century CE based on archaeological evidence. The main chamber was heavily destroyed due to grave robbing. The subsidiary chamber contained two sacrificed individuals. One individual had her head oriented towards the east, the other individual to the west. However, the two samples from the two individuals turned out to be duplicates.

Archaeological ID	Sample ID	Age	Sex	Chamber	Status
107	JOY046 (JOY026)	36 – 50	F	Subsidiary	Sacrificed
108	JOY026 (JOY046)	31 – 60	F	Subsidiary	Sacrificed

- Joyeong EIII-8

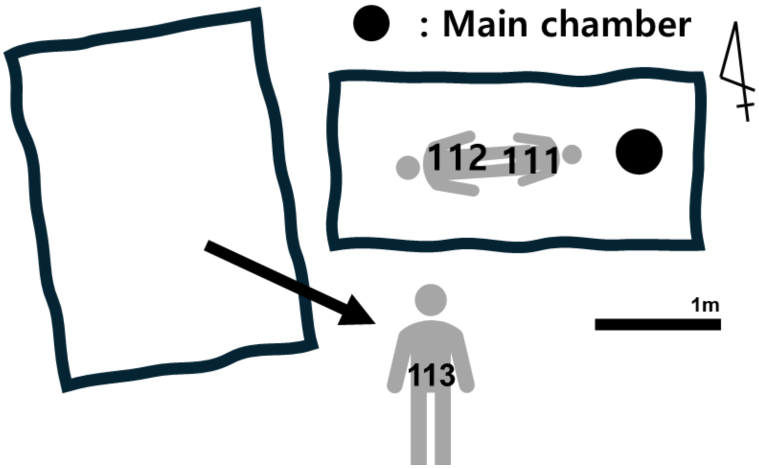

Joyeong EIII-8 is a wooden chamber of type A. The tomb was contextually dated to the second quarter of the 5th century CE based on archaeological evidence. Two individuals were discovered from the main chamber. One individual only had the crania remaining oriented towards the east and was considered as the grave owner. The other individual, who was sacrificed, had her head toward the west and was stacked upon the grave owner. The right tibia and fibula of the sacrificed individual extended beneath pottery placed towards the east, while the left tibia and fibula were bent towards the south.

Archaeological ID	Sample ID	Age	Sex	Chamber	Status
112	JOY049	15	F	Main	Sacrificed

- Joyeong EIII-26

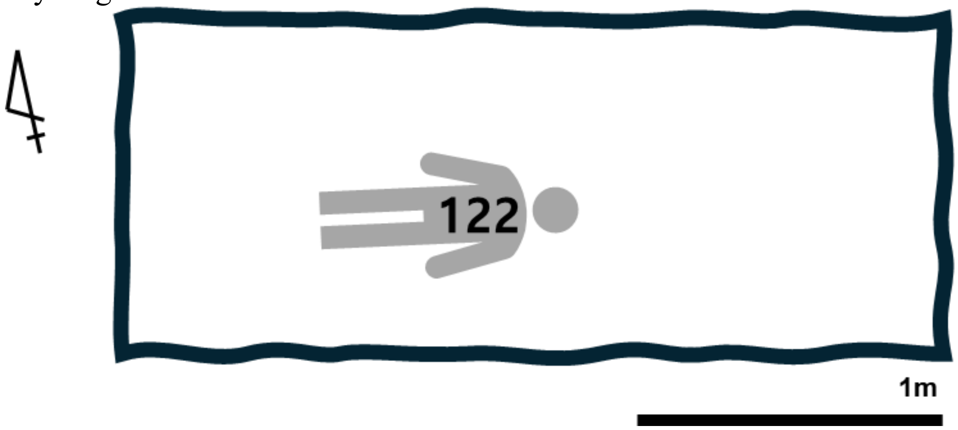

Joyeong EIII-25 is a tomb with a single wooden chamber. The date of the grave is unknown; however, it is likely between the 3rd and 4th century CE. Only one individual was uncovered, who was the grave owner. The individual's head was oriented towards the east. The bones were in bad condition, as only the crania, femur, and tibia remained.

Archaeological ID	Sample ID	Age	Sex	Chamber	Status
122	JOY053	21 – 35	M	Inside	Owner

- Joyeong EIII-29

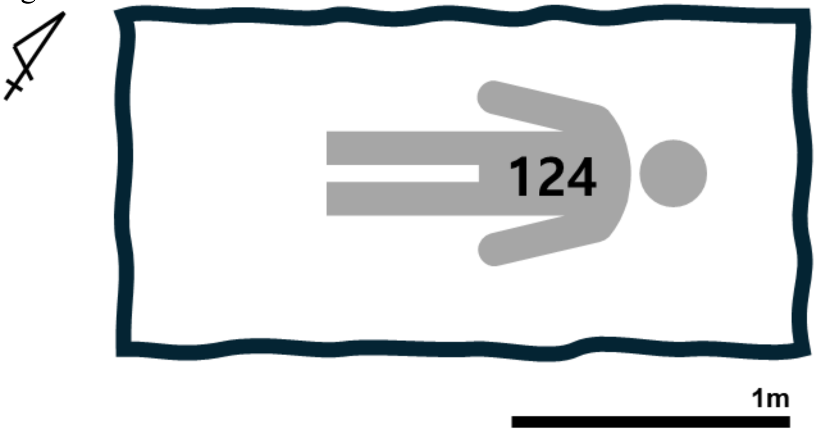

Joyeong EIII-29 is a tomb with a single wooden chamber. The date of the grave is unknown. Only one individual was discovered, who was the grave owner. The head was oriented towards the east, with only part of the legs remaining.

Archaeological ID	Sample ID	Age	Sex	Chamber	Status
124	JOY054	adult	M	Inside	Owner

- Joyeong EIII-31

No archaeological information is available for tomb Joyeong EIII-31.

Archaeological ID	Sample ID	Age	Sex	Chamber	Status
126	JOY056	adult	F	?	Unknown

- Joyeong EIV-8

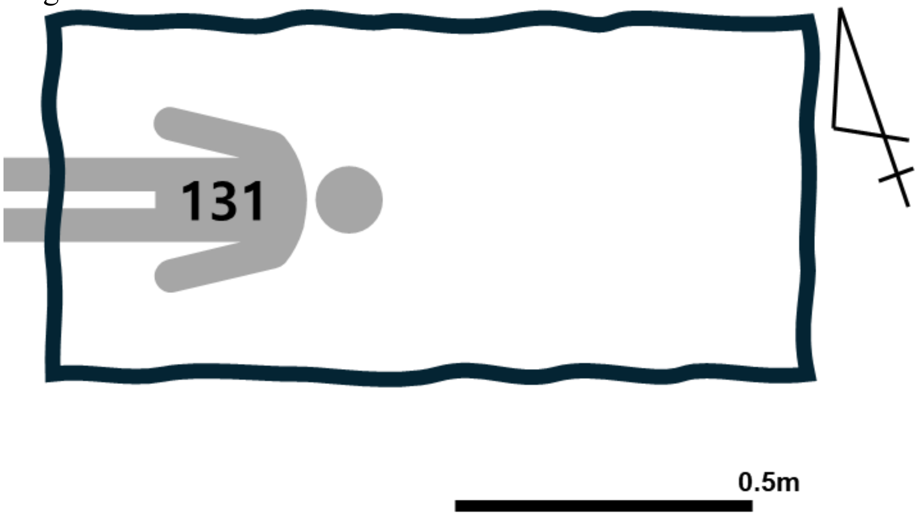

Joyeong EIV-8 is a tomb with a single wooden chamber. The date of the grave is unknown. Only one individual was discovered, who was the grave owner. The bones were not well preserved.

Archaeological ID	Sample ID	Age	Sex	Chamber	Status
131	JOY059	36 – 50	M	Inside	Owner

- Joyeong EIV-27

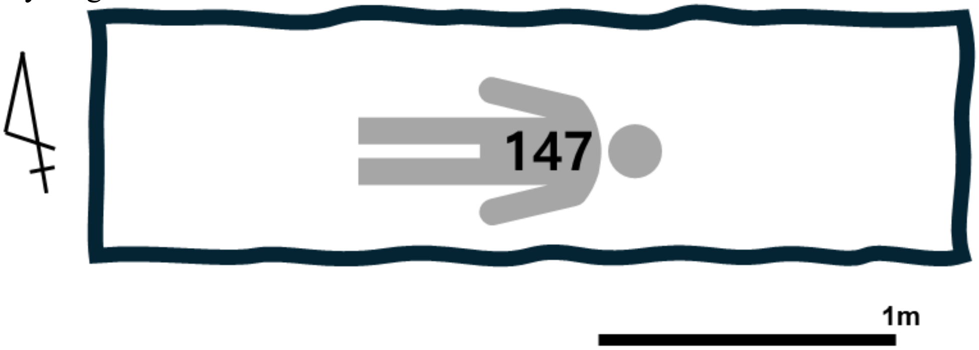

Joyeong EIV-27 is a tomb with a single wooden chamber. The date of the grave is unknown. Only one individual was discovered, who was the grave owner. The bones were not well preserved.

Archaeological ID	Sample ID	Age	Sex	Chamber	Status
147	JOY067	31 – 50	F	Inside	Owner

- Joyeong EIV-39

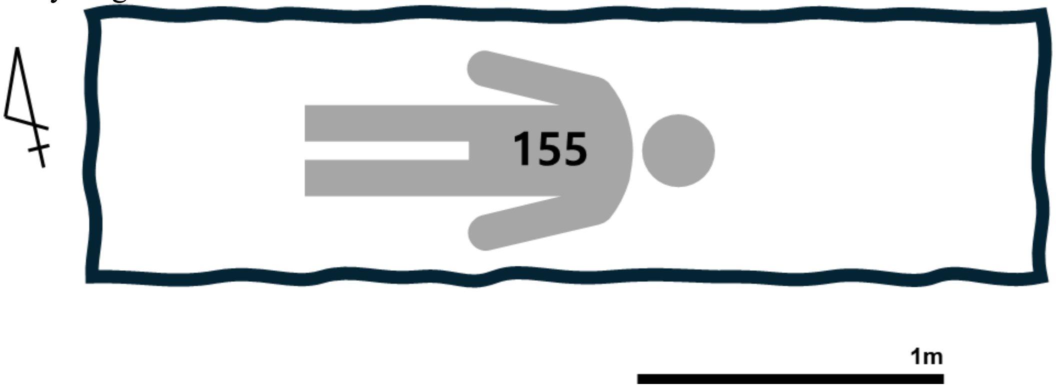

Joyeong EIV-39 is a tomb with a single wooden chamber. The date of the grave is unknown. Only one individual was discovered, who was the grave owner. The bones were not well preserved.

Archaeological ID	Sample ID	Age	Sex	Chamber	Status
155	JOY073	36 – 50	M	Inside	Owner

Supplementary Text 2. Pedigree construction

S2.1 Pedigree construction general strategy

To construct the pedigree, we considered the likelihood information from KIN, the PMR value, the uniparental haplogroups (MT and Y), estimated age at death, and order of construction of the tomb. When there were signals of long ROH, we considered the possible scenarios of consanguineous marriage. Individuals buried at the Imdang burial complex were prefixed as IMD, and individuals buried at the Joyeong burial complex were prefixed as JOY.

For KIN results, we considered all classifications of kinship except the following:

1. We filtered out relationships when the difference between log likelihoods of the most likely model and the null model (of no relatedness) is <2 to exclude spurious distant relationship due to low coverage sequencing data.
2. We removed relationships when the 5th degree had the highest log likelihood value unless we detect IBD sharing by ancIBD.
3. For individuals with the 1240K site mean coverage under 0.05x, we did not consider relationship ranges farther than second-degree since KIN may not have sufficient statistical power to distinguish between distant relatives (3rd degree or more) and the unrelated individual pairs.
4. For pairs of individuals that were both either contaminated or had no contamination estimate, we did not include the kinship relationship unless there was an uncontaminated individual supporting kinship between the two individuals. For our analysis, there were only two pairs of individuals excluded by this criterion (IMD033-JOY107, JOY078-JOY083)

We applied this procedure to recover robust relative pairs ranging between the 1st and 4th degrees among the Imdang-Joyoung individuals.

- Resolving first-degree relationships

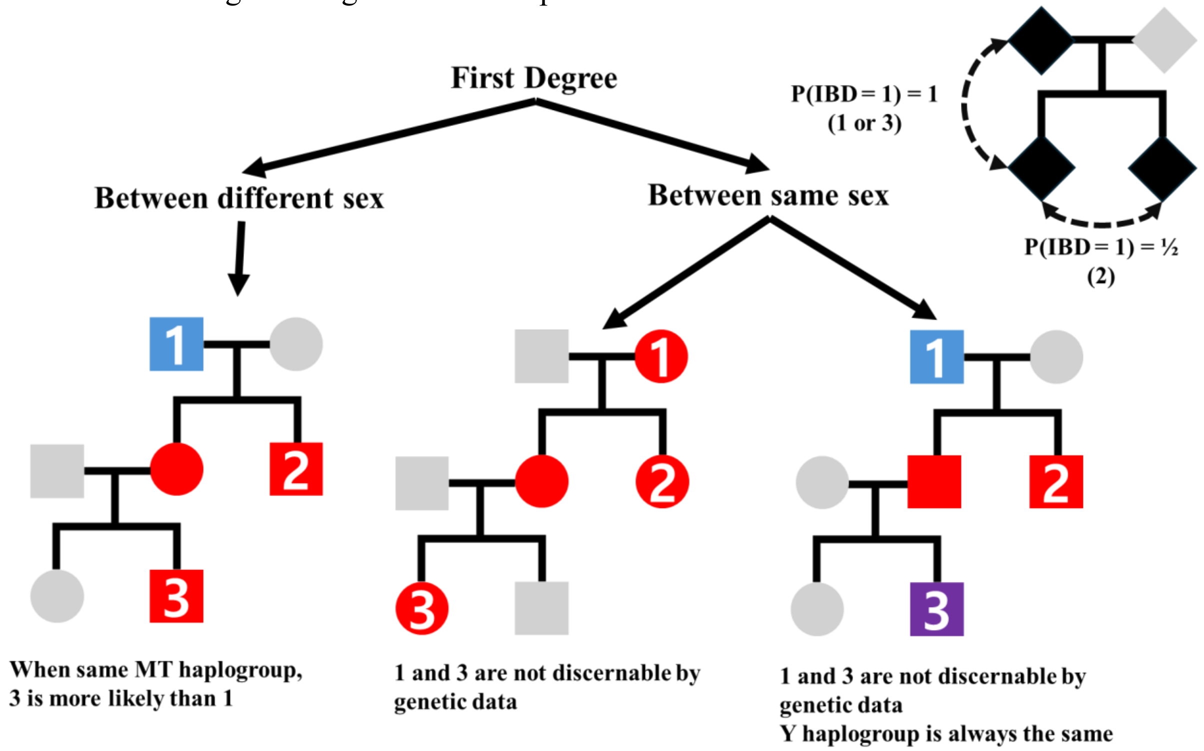

We resolved first-degree relationships based on KIN classification results and uniparental haplogroup information (mitochondrial and Y haplogroup). First-degree relationships are either parent-offspring or full siblings. The simplest case is when two unrelated individuals (one male and one female) share a first-degree relationship with a single individual, suggesting a father-mother-offspring trio.

Because mitochondrial haplogroup is inherited from mother to offspring, and Y haplogroup from father to male offspring, uniparental haplogroups can be used to validate and solidify first-degree relationships.

When a male-female pair holds a first-degree relationship, uniparental haplogroups can rule out unlikely scenarios. If the two have different mitochondrial haplogroups, they cannot be in a sibling or mother-son relationship, confirming a father-daughter relationship. When mitochondrial haplogroups are the same, parent-offspring vs. full sibling relationships can be distinguished primarily by the estimates of IBD0/1/2 from KIN: parent-offspring pairs are supposed to share only one copy of chromosome (IBD1) across the genome, while full siblings are supposed to share 0, 1, or 2 copies of chromosomes with 0.25, 0.5, 0.25 probabilities across the genome, respectively. If the relationship is parent-child, it is likely that the female is the mother of the male, although the opposite is not impossible.

When two individuals are of same sex, males should always have same Y haplogroups, while females should always share a mitochondrial haplogroup. Discrepancy in uniparental haplogroup and first-degree kinship indicates a faulty haplogroup call or a misclassification of kinship, which may happen due to low coverage of sequence data from the corresponding region (mitochondria, Y chromosome, or autosomes).

- Resolving relationships where three individuals share second-degree kinship with each other

Second-degree relationships with no inbreeding fall under four categories: grandparent-grandchild, avuncular, half-sibling, and double cousin. A levirate union where brothers or sisters share a spouse result is a special case of the two offspring having a half-sibling relationship that is slightly closer than second-degree.

In the case of three individuals with second-degree kinship without consanguineous marriage, the pedigree cannot be resolved unless there is at least one half-sibling or double-cousin relationship.

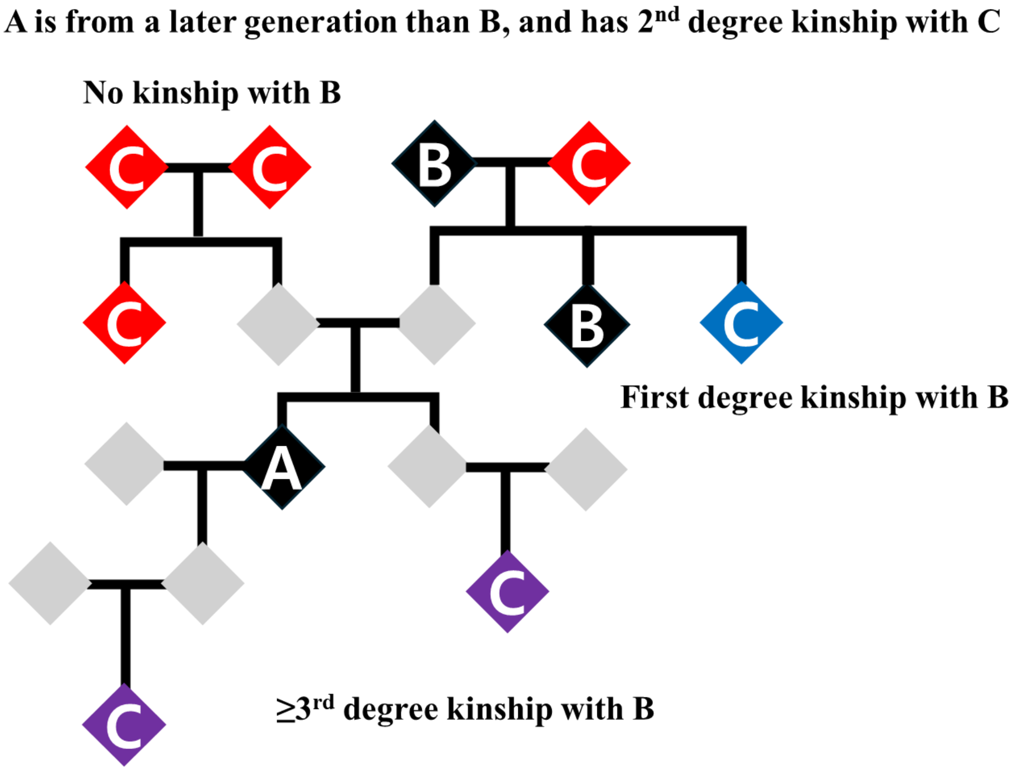

Let's assume A and B have a second-degree kinship which is either a grandparent-grandchild or avuncular relationship, and A is from a younger generation than B (grandchild or nephew), and is related through X, either the mother or father of A. If A has a grandparent-grandchild or avuncular relationship with C, there are three possible scenarios: C can be related to one generation up through X, C can be related to the other parent that is not X, or C is of younger generation than A. In the first case, C is either first-degree (sibling/offspring) or unrelated (married) with B. In the second case, C is unrelated to B by kinship. In the third case, C is 3rd or 4th degree with B. Therefore, at least one half-sibling or double-cousin relationship is required for three individuals to share a second-degree relationship.

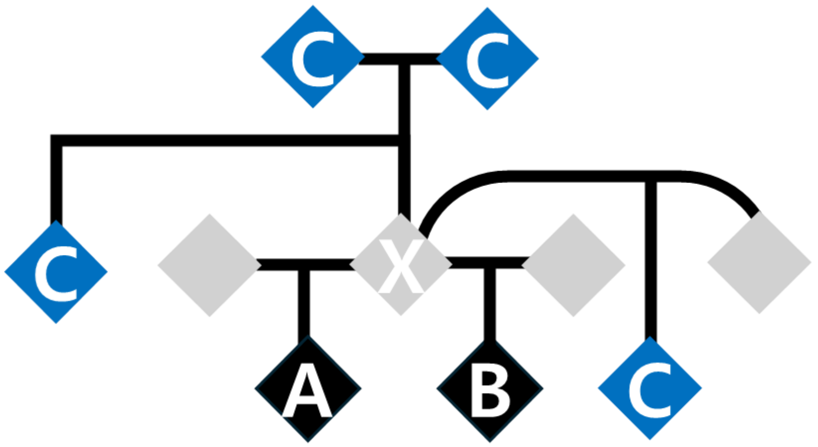

All possible positions of C given A and B are half-siblings

If there is a half-sibling relationship between A and B through X, a common parent, any individual C with a first-degree relationship through X will be related closer at least 2nd degree to A and B. If C is a child of X, it should be half-siblings with A and B to be related by 2nd degree with each of them (should not be first-degree siblings).

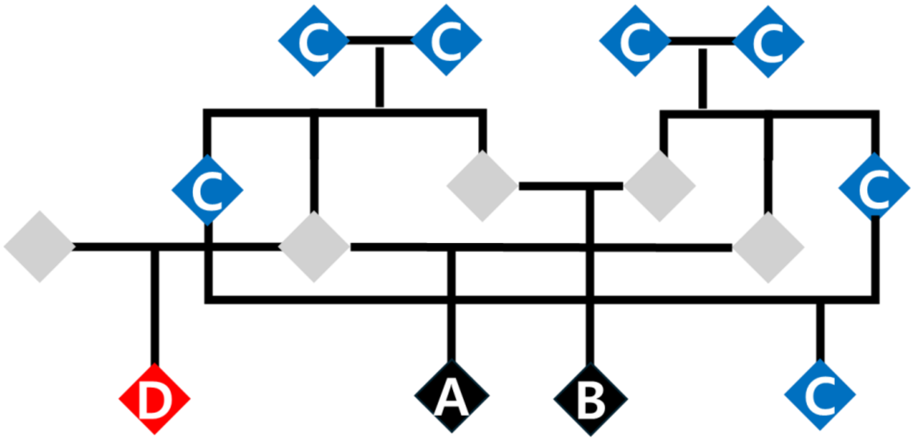

All possible positions of C given A and B are double cousins
C cannot be a half-sibling of A or B unless C is born from a levirate union

If there is a double-cousin relationship between A and B, a third individual C can be a third double-cousin. C can also be a grandparent, uncle or aunt of A and B. C cannot be a half-sibling of A with an unrelated founder or sibling of A's parent, since C will be 3rd degree related to B unless C is born from a levirate union.

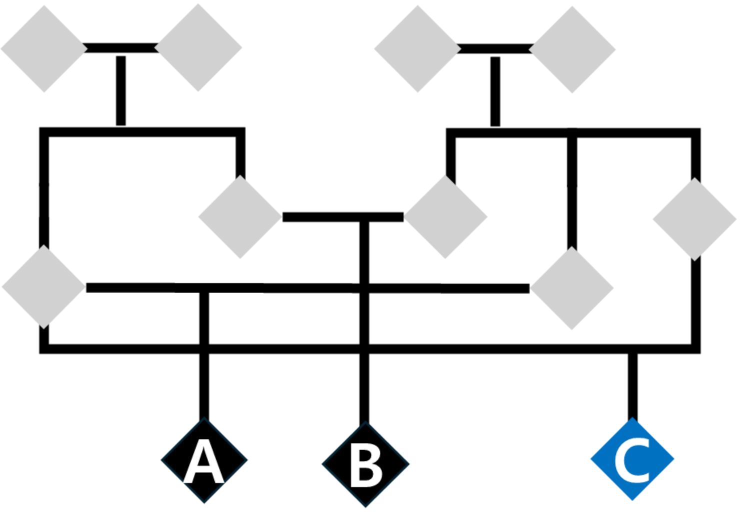

C can be a half-sibling of A if it is born from a levirate union

- Resolving half-siblings based on uniparental haplogroups

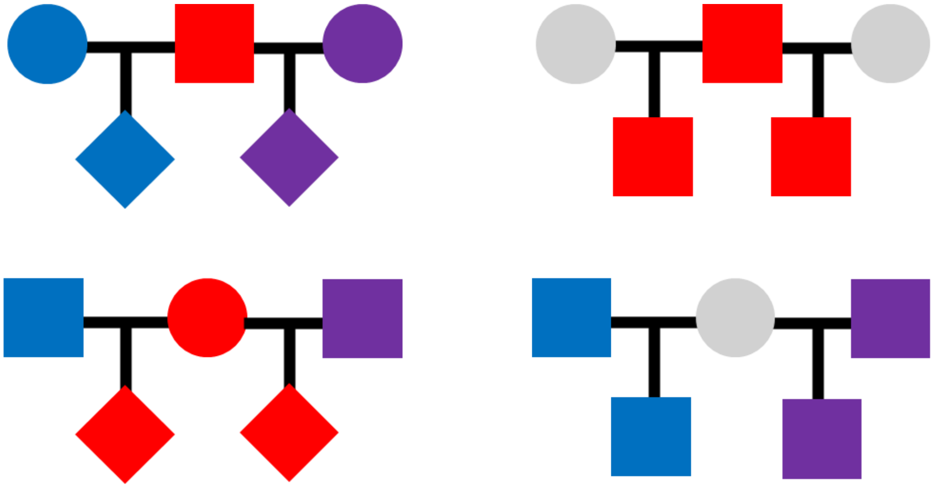

MT haplogroup sharing can rule out the shared parent's sex

Y haplogroup sharing can rule out the shared parent

In the case of half-siblings, they can either share a mother or a father. Different mitochondrial haplogroups rule out the possibility of maternal sharing. Same mitochondrial haplogroups may indicate a shared mother, although it is possible that there was female levirate union or same mitochondrial haplogroup mothers with a shared father. Similarly, different Y haplogroups between half-sibling brothers rules out the possibility of a shared father.

S2.2 Pedigree construction of each family

First degree relationships (n = 11)

We first started with 1st degree relationships. KIN discovered 12 pairs of 1st degree relationships, 9 classified as parent-offspring and 3 classified as full siblings. Among the 12 relationships, one relationship was considered as from the same sample instead of a first-degree relationship.

JOY034 (070) – JOY035 (073)

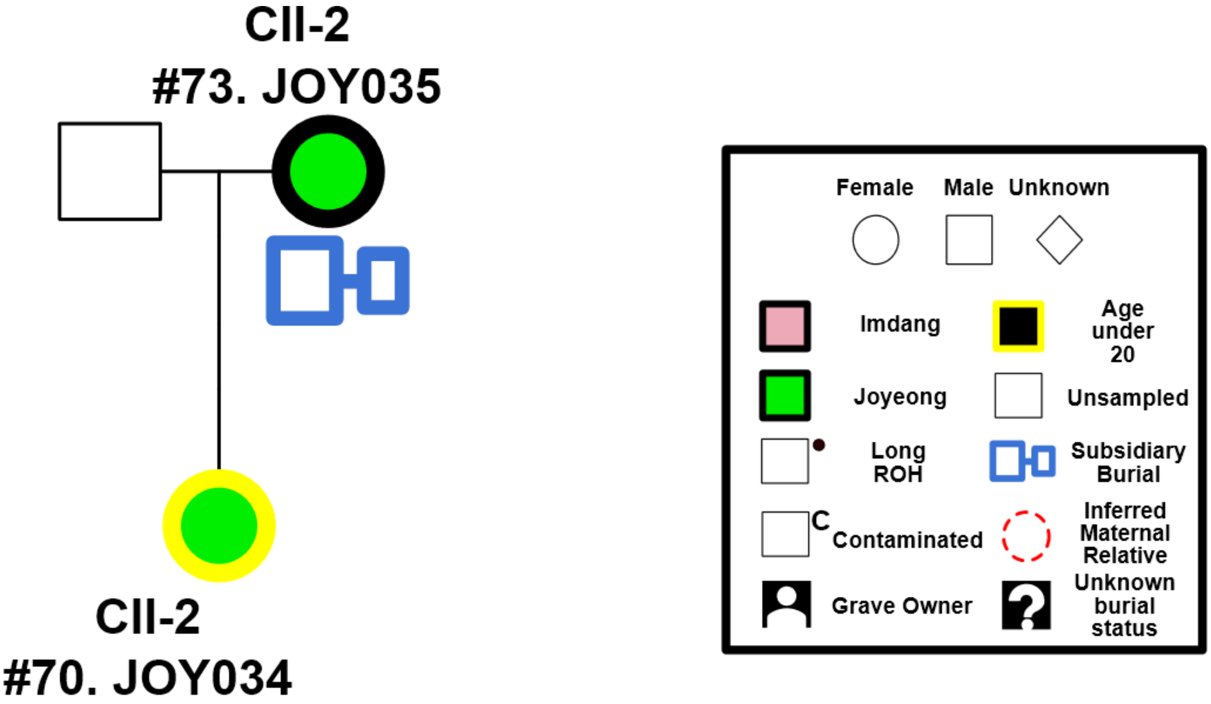

For JOY034 and JOY035 KIN results, a parent-child relationship had the highest likelihood value with likelihood values for second-degree relationships within value 1 with the max likelihood value due to low SNP coverage of both individuals. JOY034 and JOY035 both shared the same mitochondrial haplogroup B5a2a1, and genetic information alone cannot decide who the mother is. Based on the age of JOY035 being between 21 and 35, while JOY034 was at age 10, JOY035 was likely the mother of JOY034.

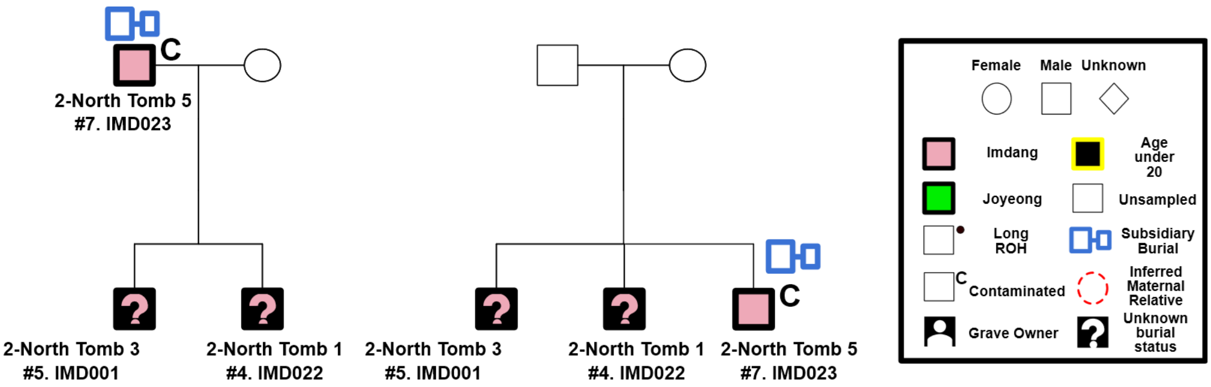

For IMD001 and IMD022 KIN results, sibling relationship had the highest likelihood value with likelihood values for a parent-child relationship within value 1 with the max likelihood value, while between IMD023 and either IMD001 or IMD022, a parent-child relationship had the highest likelihood value. For IMD001 and IMD023, a sibling relationship was within value 1 with the max likelihood value. IMD001 and IMD022 shared MT haplogroups (M8a3a), reinforcing the kinship between them being siblings. IMD023 had not enough genomic coverage for a reliable estimate of the MT haplogroup, so by genomic data alone, there was uncertainty whether IMD023 was truly the father of IMD001 and IMD022. Age information also collides with a parent-child relationship due to the three individuals coming from the same tomb and having similar age (IMD001 and IMD022 were between the age of 21 and 35; IMD023 was estimated to be 30).

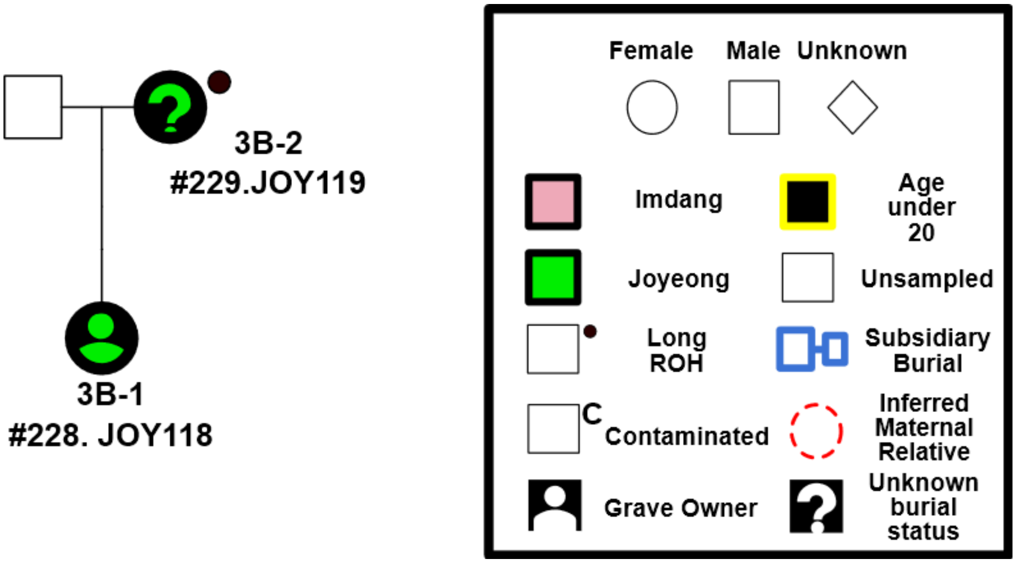

For JOY118 and JOY119 KIN results, parent-child relationship had the highest likelihood value. JOY118 and JOY119 shared the same MT haplogroup F2b1, and genetic data alone cannot decide who was the mother. JOY119 was likely the mother of JOY118 based on age estimates for JOY119 being between either 21 – 35 or 41 – 60, compared to the age estimate of JOY118 which was 21 – 35.

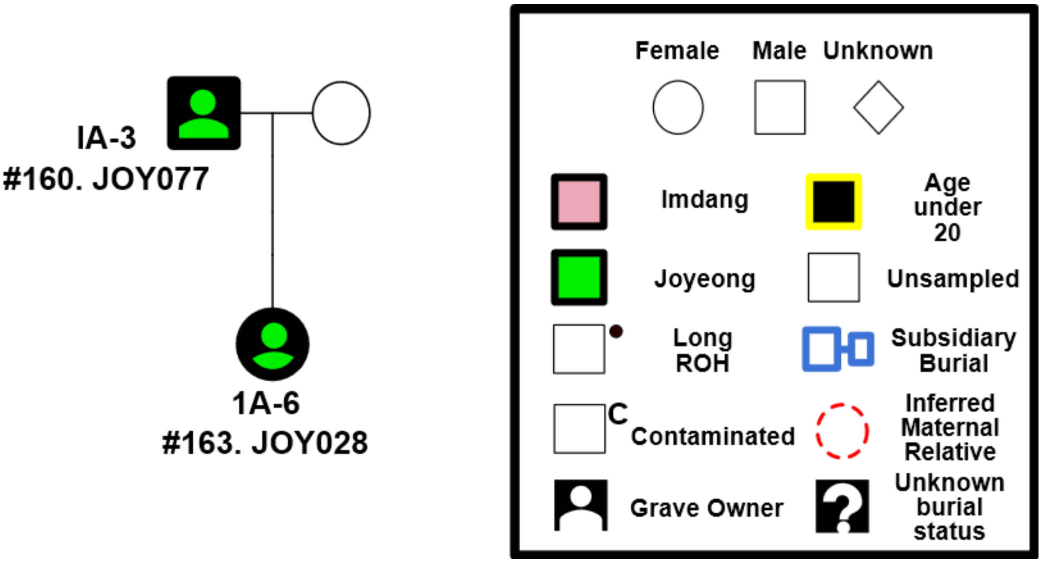

For JOY028 and JOY077 KIN results, parent-child relationship had the highest likelihood value. JOY028 had MT haplogroup D4b2b2 while JOY077 had haplogroup G3a, suggesting JOY077 was the father of JOY028. Age information did not coincide with this inference due to both individuals being estimated to be between 21 and 35.

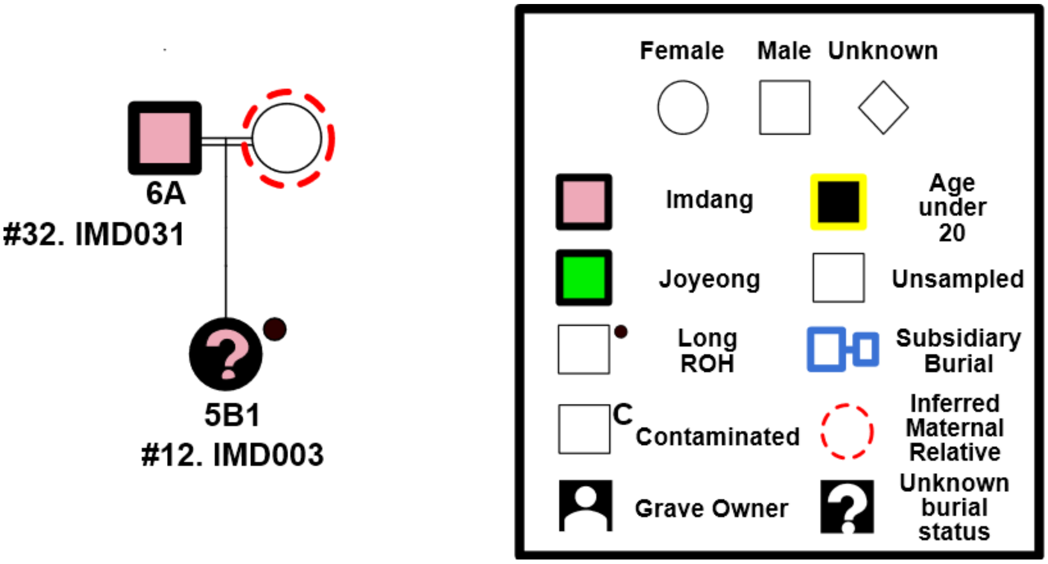

For IMD003 and IMD031 KIN results, parent-child relationship had the highest likelihood value. MT haplogroup of IMD031 was D4b2a2a1, while for IMD003 it was C4a1a+195, suggesting IMD031 was the father of IMD003. IMD031 is estimated to be of age 41 – 60, while IMD003 is estimated to be 21 – 35 or 36 – 50.

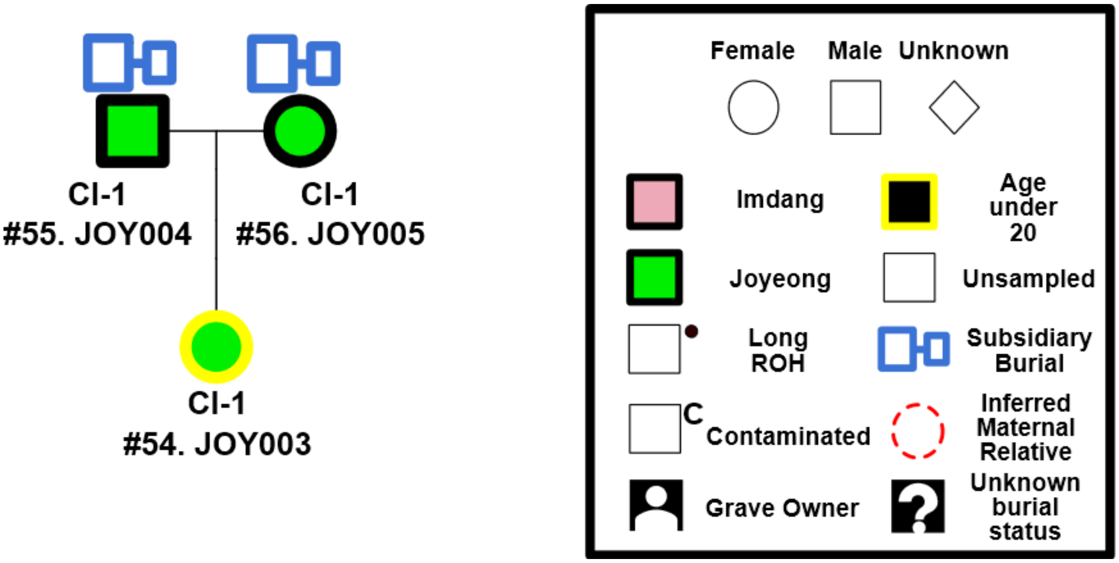

For KIN results of JOY003 between JOY004 and JOY005, parent-child relationship had the highest likelihood value. JOY004 and JOY005 did not share kinship within 4th degree based on KIN results and did not share any IBD longer than 12cM based on ancIBD. JOY003 and JOY005 shared same MT haplogroup D4b2a2, while JOY004 had MT haplogroup G1a3, suggesting JOY004 was the father of JOY003. If JOY003 was the mother of JOY005, JOY004 and JOY005 should share a grandparent-grandchild relationship, which was not true. Therefore, based on genetic evidence, JOY004 and JOY005 were the parents of JOY003.

This is in line with age estimates, where JOY003 was between the ages of 4 and 8, while JOY004 and JOY005 were between 41 – 60 and 36 – 50, respectively.

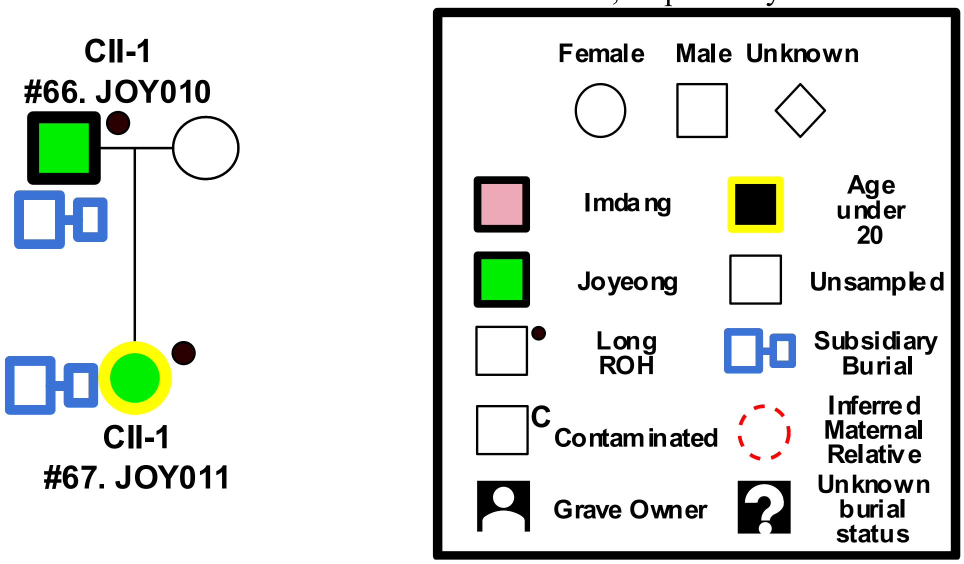

For KIN results of JOY010 and JOY011, parent-child relationship had the highest likelihood value. JOY010 had MT haplogroup D4a, while JOY011 had MT haplogroup D5b1c. The different MT haplogroups suggested that JOY010 was the father of JOY011. This is in line with the age estimate, where JOY010 was at the age between 36 and 50, while JOY011 was 10 years old.

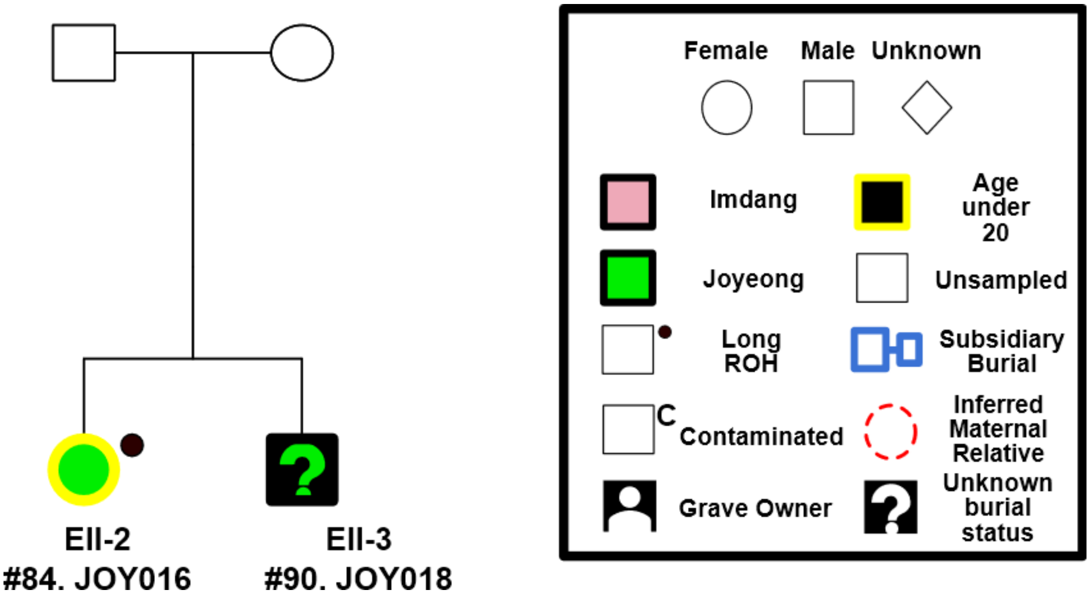

For KIN results of JOY016 and JOY018, full sibling relationship had the highest likelihood value. The two individuals shared the same MT haplogroup N9a4a. Age information reinforces a full sibling relationship since JOY016 cannot be the mother of JOY018.

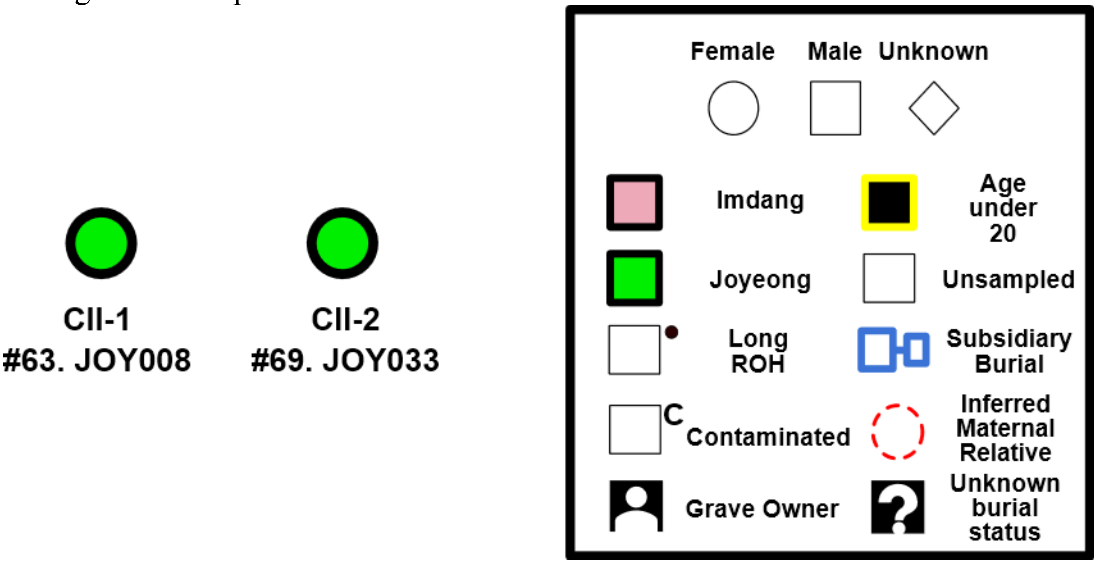

For KIN results of JOY008 and JOY033, sibling relationship had the highest likelihood value. However, the two individuals shared a pairwise mismatch rate of 0.108 from 7,673 SNPs, which is an unlikely value between siblings. The MT haplogroup estimate for JOY033 was not available, and JOY008 had 1240K SNP depth coverage of 0.054. Based on this observation, we classified the two individuals to be duplicate samples.

Relationships further away from 1st degree

KIN detected 22 2nd degree, 13 3rd degree, and 19 4th degree relationships.

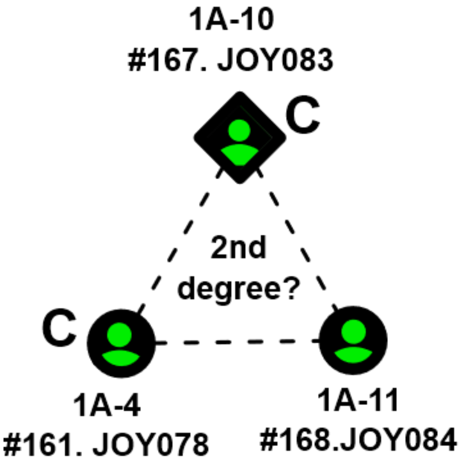

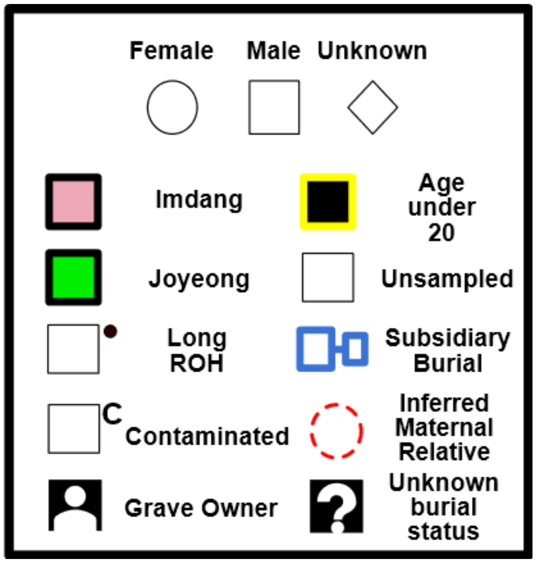

For KIN results of JOY078 and JOY083, an avuncular relationship had the highest likelihood value. For JOY078 and JOY084, an avuncular relationship had the highest likelihood value with a half-sibling relationship having likelihood value within 1 of the highest likelihood value. For JOY083 and JOY084, an avuncular relationship had the highest likelihood value with a grandparent-grandchild relationship having likelihood value within 1 of the highest likelihood value. No estimates for both MT and Y haplogroups were available, and JOY078 and JOY083 were flagged for contamination.

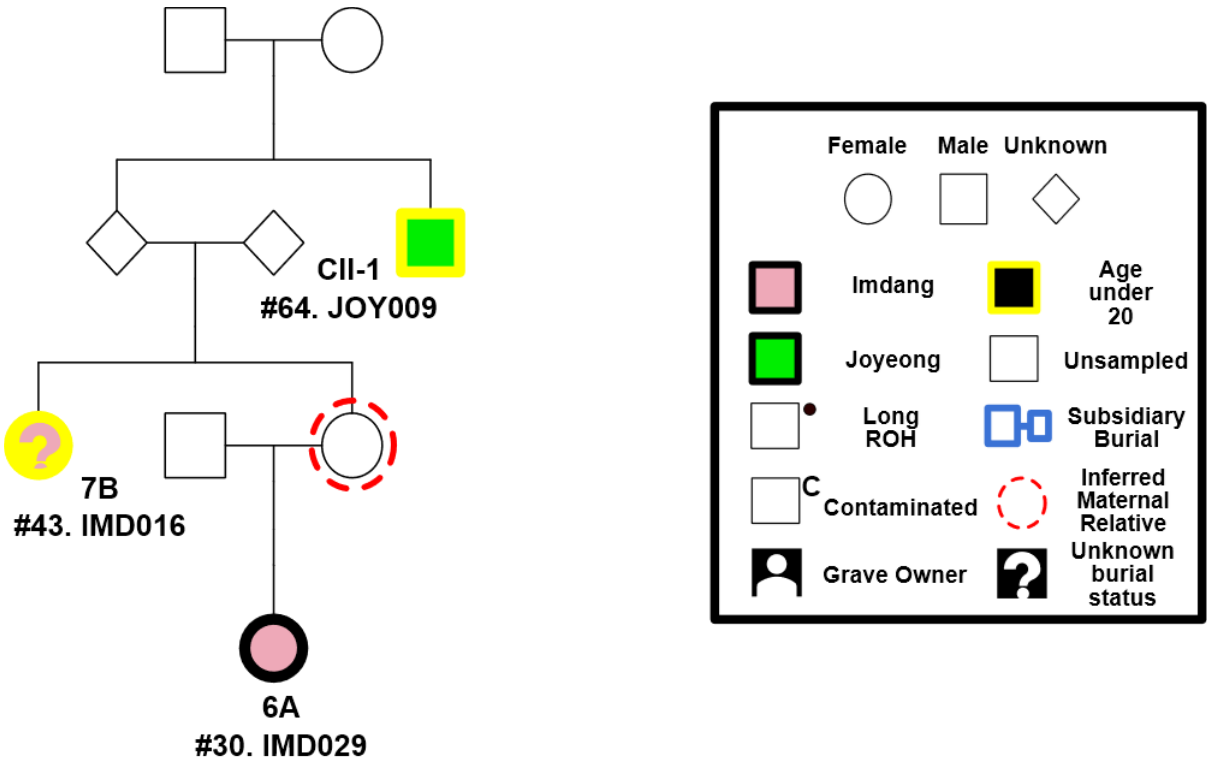

For KIN results between IMD016 and IMD029, an avuncular relationship had the highest likelihood with a half-sibling relationship having likelihood value within 1 of the max likelihood value. IMD016 and JOY009 had the max likelihood value for an avuncular relationship. JOY009 and IMD029 had 4th degree with the highest likelihood value and 3rd degree within difference of 1 of the max likelihood value, although IMD029 had 1240K SNP depth of 0.04 x, making this estimate unreliable. The difference of kinship between IMD016 and IMD029 with JOY009 suggests that IMD016 and IMD029 share an avuncular relationship rather than a half-sibling relationship. IMD016 and IMD029 shared MT haplogroup D4b2a, while JOY009 had MT haplogroup B4a1b1. Based on the plausible relationships, IMD016's sister was likely the mother of IMD029.

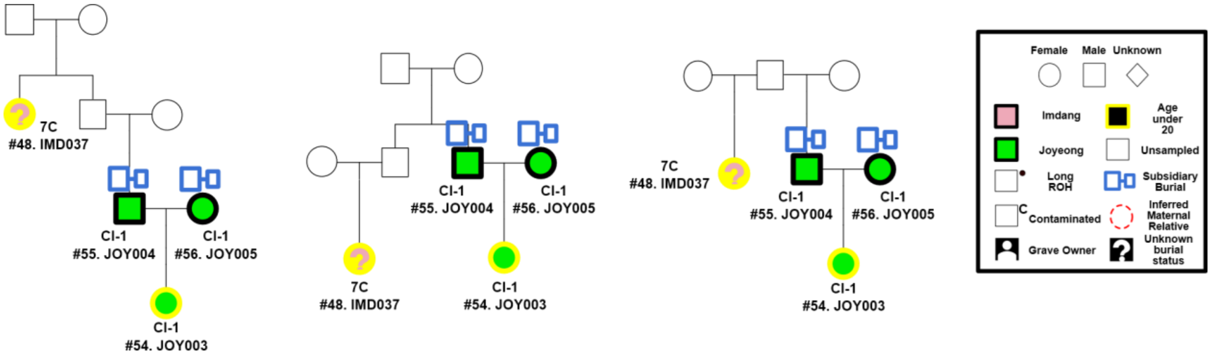

Based on KIN results, IMD037 and JOY004 had max likelihood values for an avuncular relationship, while having likelihood values for a half-sibling relationship within the difference of one with the max likelihood value. IMD037 and JOY004 had different MT haplogroups. Based on the fact that tomb 7C was built earlier than CI-1, the first scenario of IMD037 being the aunt of JOY004 seems more likely, although alternative pedigrees may be possible.

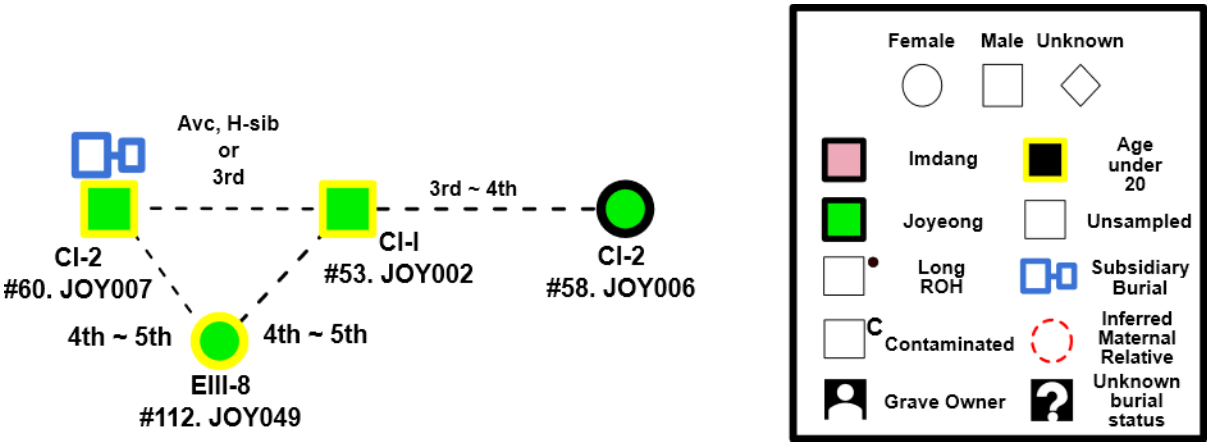

For KIN results of JOY002 and JOY007, an avuncular relationship had the highest likelihood value with a half-sibling or 3rd degree relationship having likelihood value within 1 of the max likelihood value. For JOY002 and JOY006, a 3rd degree relationship had the highest likelihood value with a 4th degree relationship having likelihood value within 1 of the max likelihood value. JOY049 shared IBD blocks with JOY002 and JOY007 detected in ancIBD, with 5th degree relationships in KIN. All individuals did not share any uniparental haplogroups, ruling out the possibility of JOY002 and JOY007 being half-siblings.

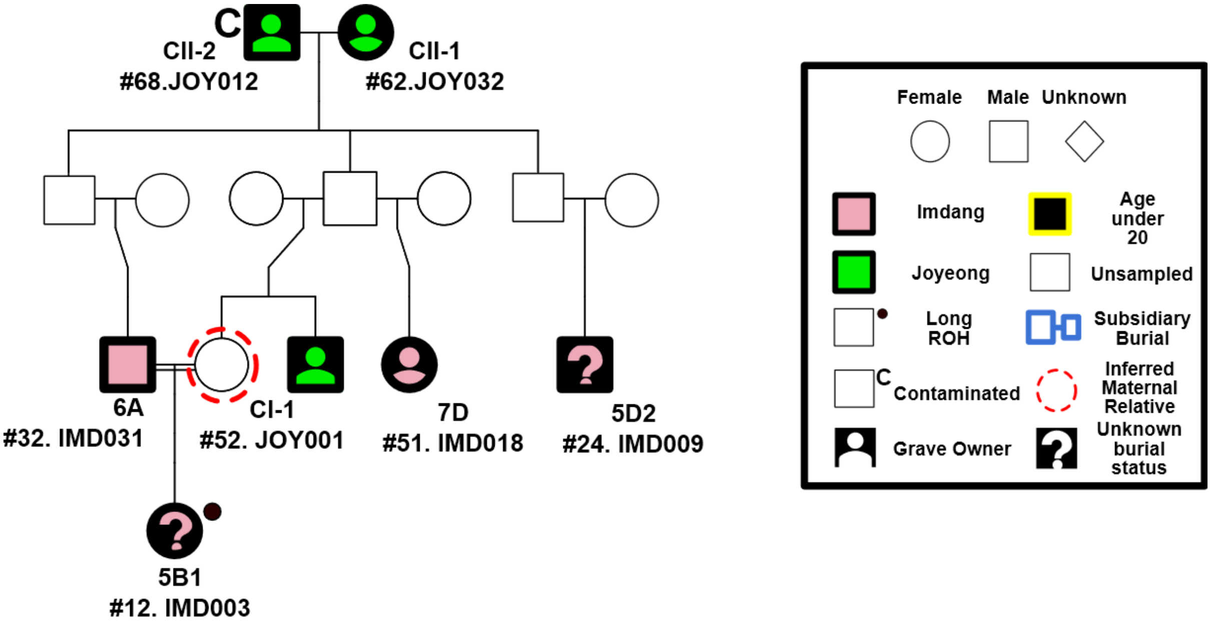

Based on KIN results, JOY032 shares second-degree relationships with IMD003, IMD009, IMD018, IMD031, and JOY001. For IMD003, JOY032 had a grandparent-grandchild or half-sibling relationship, but IMD003 was an inbred individual.

For IMD009, half-sibling relationship had the highest likelihood value, but other second-degree relationships were within value 2 of the max likelihood value. Since IMD009 and JOY032 have different MT haplogroups, if the two shared a half-sibling relationship, the two shared a father.

If JOY032 and IMD009 were half-siblings, IMD031, IMD018, and JOY001 should be 4th degree related with IMD009 if they are the grandchild of JOY032, 2nd degree related with IMD009 if they are the paternal grandparents of JOY032, or unrelated if they are the maternal grandparents. However, JOY001, IMD018, IMD031 have 3rd degree relationship as the max likelihood with IMD009, suggesting that JOY032 and IMD009 were not half-siblings.

If JOY032 is the grandchild of IMD009, the grandparent of JOY032 should be unrelated with IMD009, while the grandchild of JOY032 is 4th degree related with IMD009. Neither is the case for IMD018, IMD031, and JOY001, being 3rd degree related with IMD009. Therefore, JOY032 is the grandparent of IMD009. Similarily, JOY001, IMD018 and IMD031 are grandchildren of JOY032.

The same logic can be applied for JOY012 between the grandchildren of JOY032. We were also unable to detect kinship between JOY012 and JOY032. This suggested that JOY012 and JOY032 were married together. The uniparental haplogroups suggested that JOY012 and JOY032 were paternal grandparents of IMD009, IMD018, IMD031, and JOY001.

JOY001 and IMD018 shared a second-degree relationship with each other. Ruling out the case of double-cousins, at least one half-sibling relationship is required between JOY001, IMD018, and JOY012 or JOY032. Therefore, JOY001 and IMD018 were likely half-siblings, sharing a father based on MT haplogroups.

The daughter of IMD031, IMD003, was an inbred individual with at least full first-cousin consanguinity. Interestingly, IMD003 shared a 2nd degree relationship based on KIN with JOY001 and IMD018 while sharing a third-degree relationship with IMD009, showing closest affinity towards JOY001 among the three based on PMR. IMD003 also shared MT haplogroups with JOY001. This suggested that IMD003 was an inbred individual between IMD031 and the sister of JOY001.

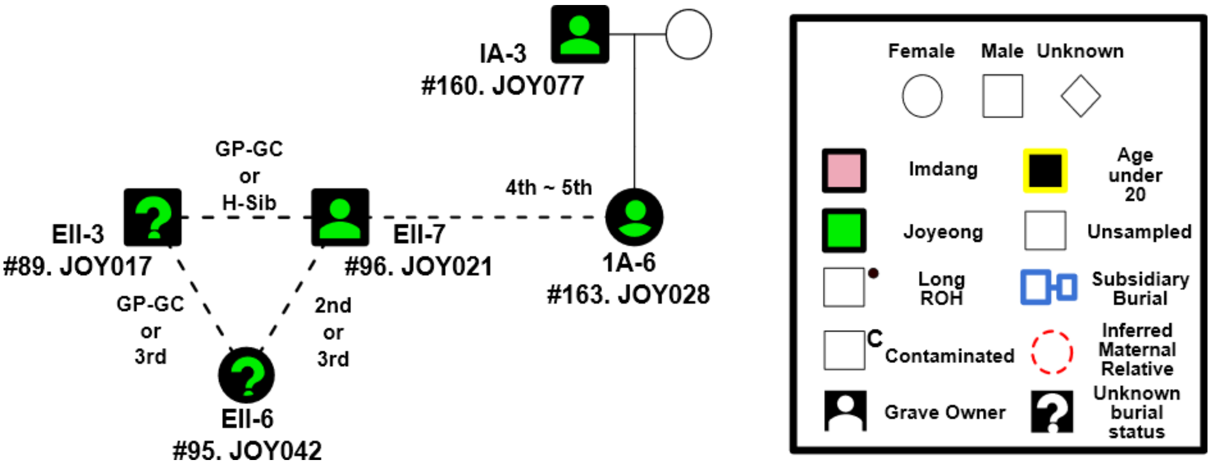

JOY021 is related to JOY017 by grandparent-grandchild relationship. JOY021 is related to JOY042 by 2nd to 3rd degree and related to JOY028 by 4th to 5th degree. JOY017 is related to JOY042 by grandparent-grandchild or 3rd degree. All three individuals did not share uniparental haplogroups. JOY28 may share 4th to 5th degree relationships with JOY21, and JOY042.

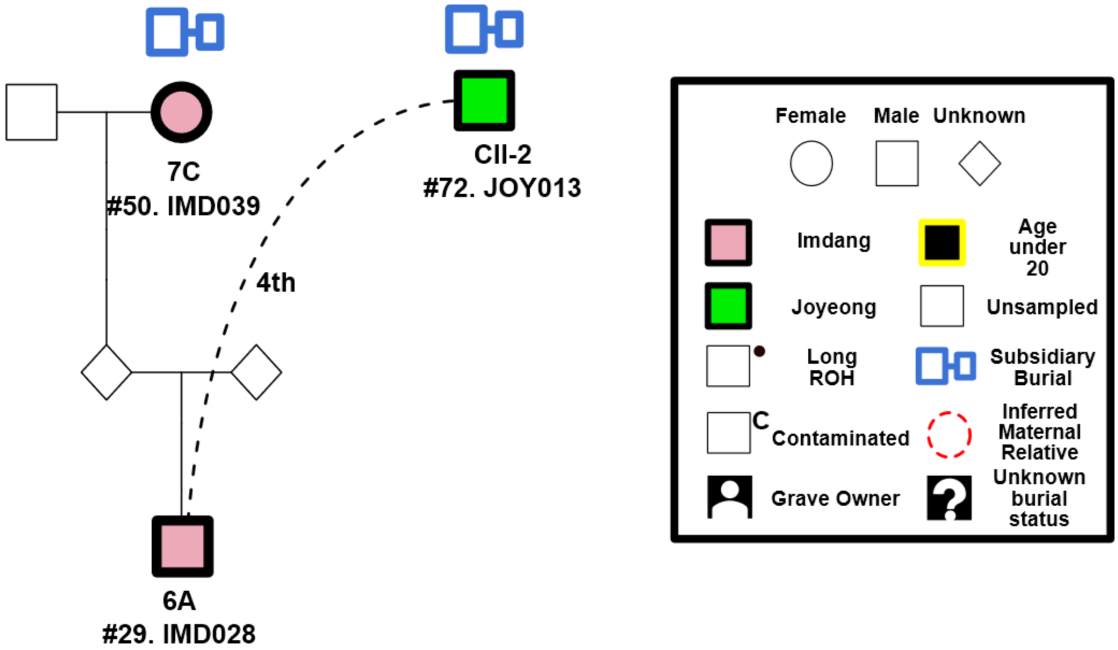

IMD028 and IMD039 shared grandparent-grandchild relationships, while JOY013 and IMD028 were 4th degree relatives. Based on the order of tomb construction, we concluded that IMD039 and JOY013 are ancestral to IMD028.

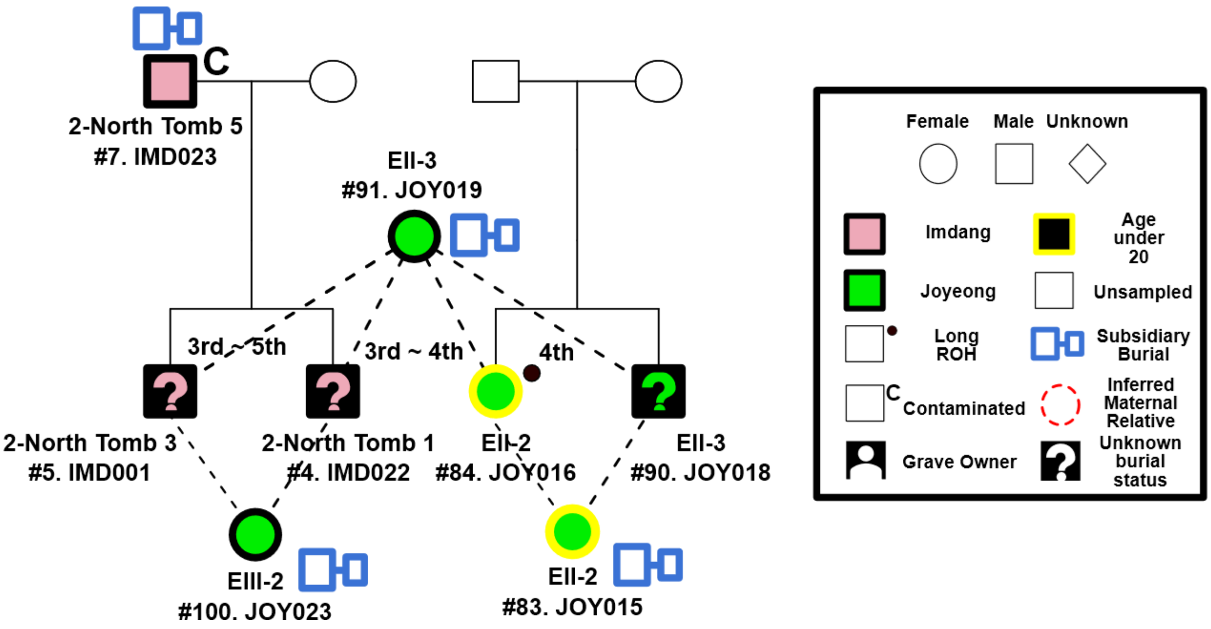

We detected relatives between the IMD001/IMD022/IMD023 family and JOY016/JOY018 family.

JOY019 had relationships from 3rd to 4th degree with IMD022, IMD023, JOY016, and JOY018. JOY015 had 3rd to 4th degree relationships with JOY016, and shared IBD over 12cM with JOY016 and JOY018.

Supplementary Figures

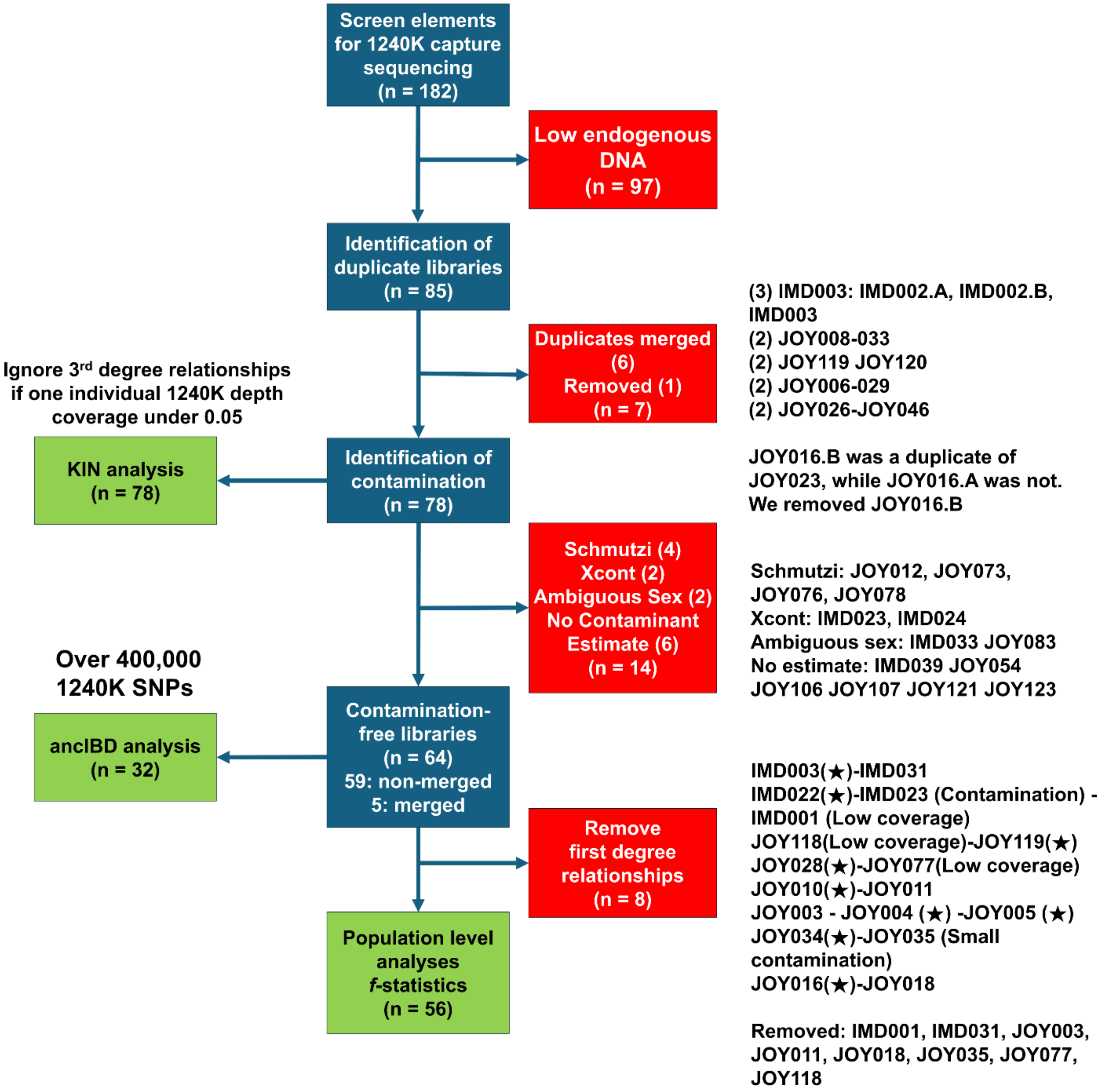

Figure S1. Data curation strategy of Imdang-Joyeong ancient libraries. We present the data curation scheme of Imdang-Joyeong ancient libraries for data analysis. Red boxes represent information and criteria of libraries that were excluded or merged with other libraries in our analysis. Green boxes represent the final dataset used for each analysis. The stars in the text next to "Remove First degree relationships" represent individuals that were kept for consecutive analysis.

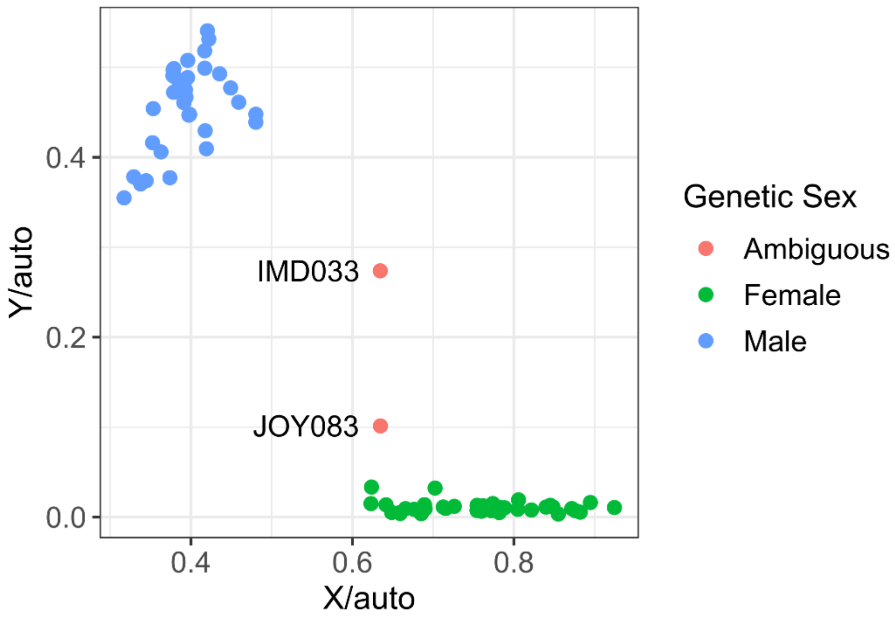

Figure S2. Genetic sex determination of Imdang-Joyeong individuals. We plotted X to autosome read coverage ratio with Y to autosome read coverage ratio to determine genetic sex. Two outlier individuals were flagged as ambiguous.

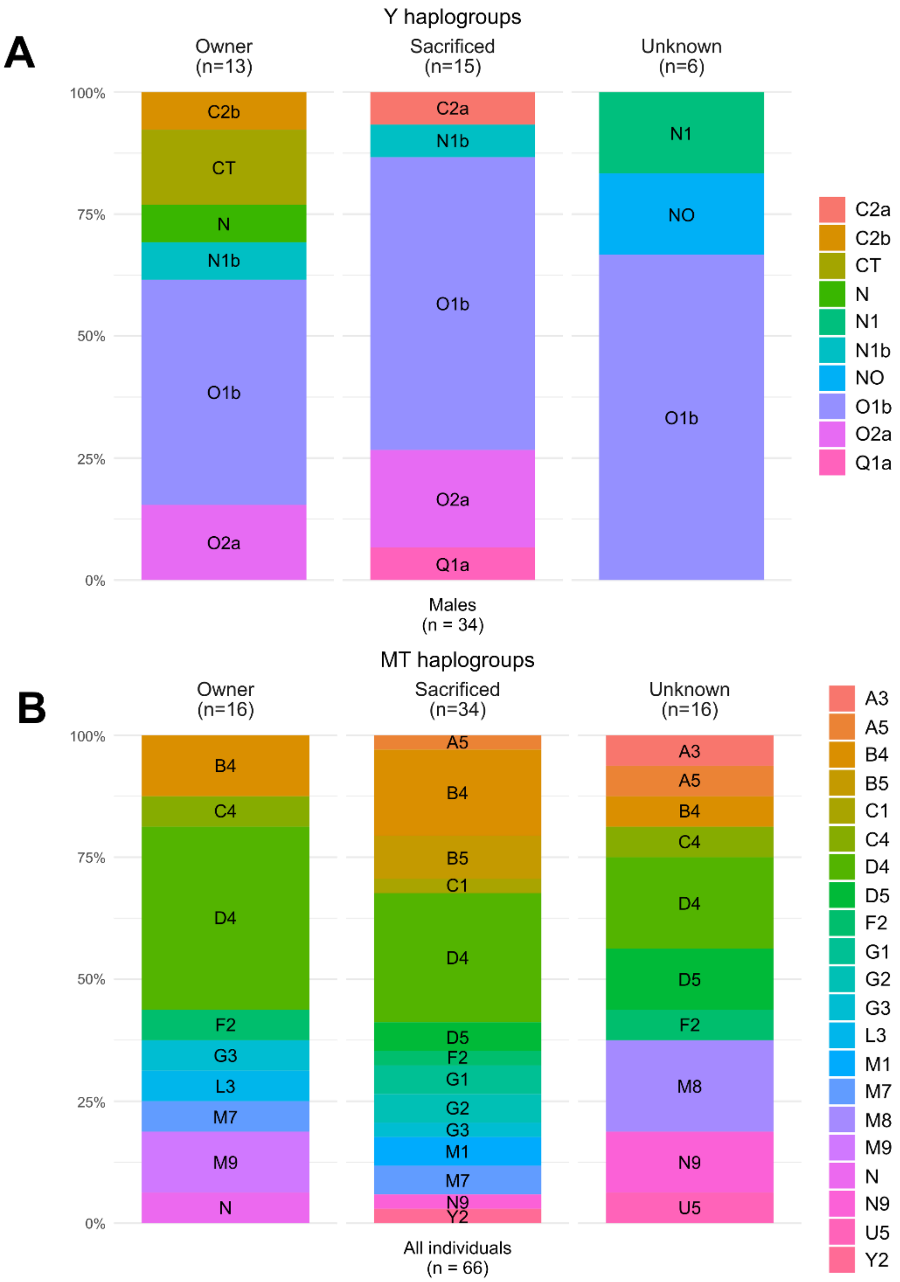

Figure S3. Uniparental haplogroup distribution of Imdang-Joyeong individuals. A. Y haplogroup proportion of Imdang-Joyeong males **B.** Mitochondrial haplogroup proportion of Imdang-Joyeong individuals. Individuals who were unable to retrieve haplogroups were excluded. For Y haplogroups, the first three letters were grouped together. For MT haplogroups, the first two letters were grouped together.

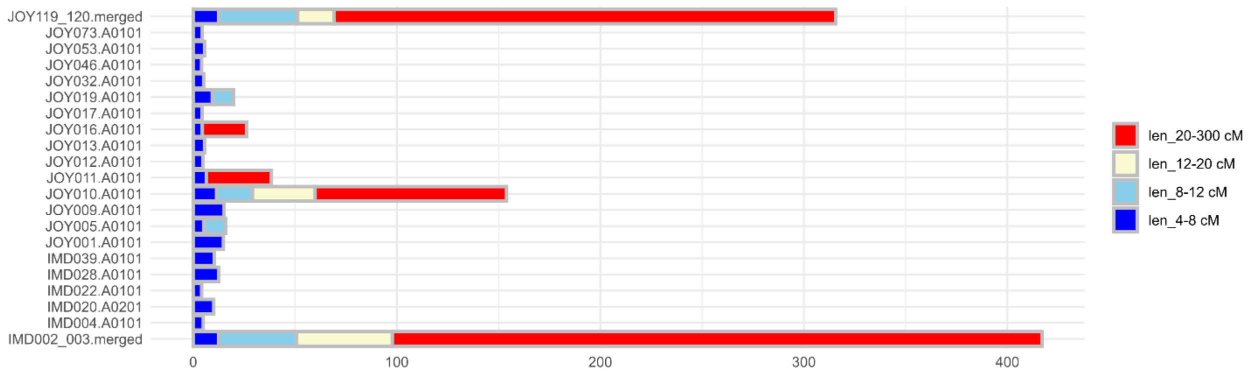

Figure S4. Length distribution of estimated ROH length using hapROH ROH segments were binned based on continuous length. Long lengths of continuous ROH segments indicate recent consanguineous marriage, while long lengths of short ROH segments indicate small effective population size. Only individuals with detected ROH are plotted. We detect 5 individuals with ROH segments longer than 20 cM.

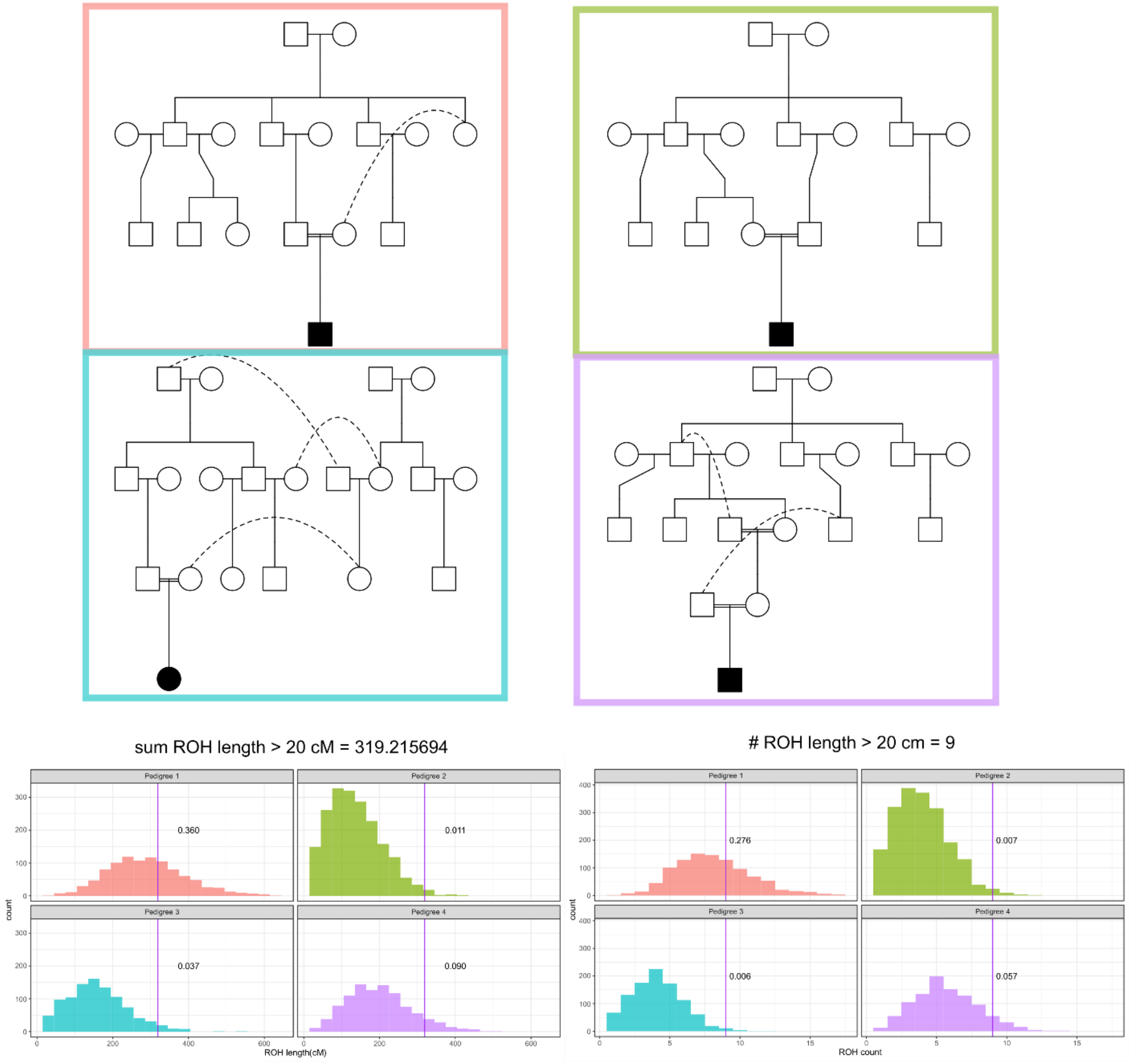

Figure S5. Assessment of multiple scenarios of consanguineous marriage and ROH segment distribution. We used Ped-sim to simulate four scenarios of consanguineous marriage to test the empirical p-value of the length and count of ROH segments longer than 20 cM for individual IMD003. Histograms were created from the result of 1000 simulations. Each panel on the bottom corresponds to the simulated results from the pedigree on the top with the same color of the box surrounding the pedigree and position of quadrant for sum of ROH length and number of ROH segments longer than 20 cM. The observed ROH length and count, along with the empirical p-values are each displayed with a vertical line and text for each panel.

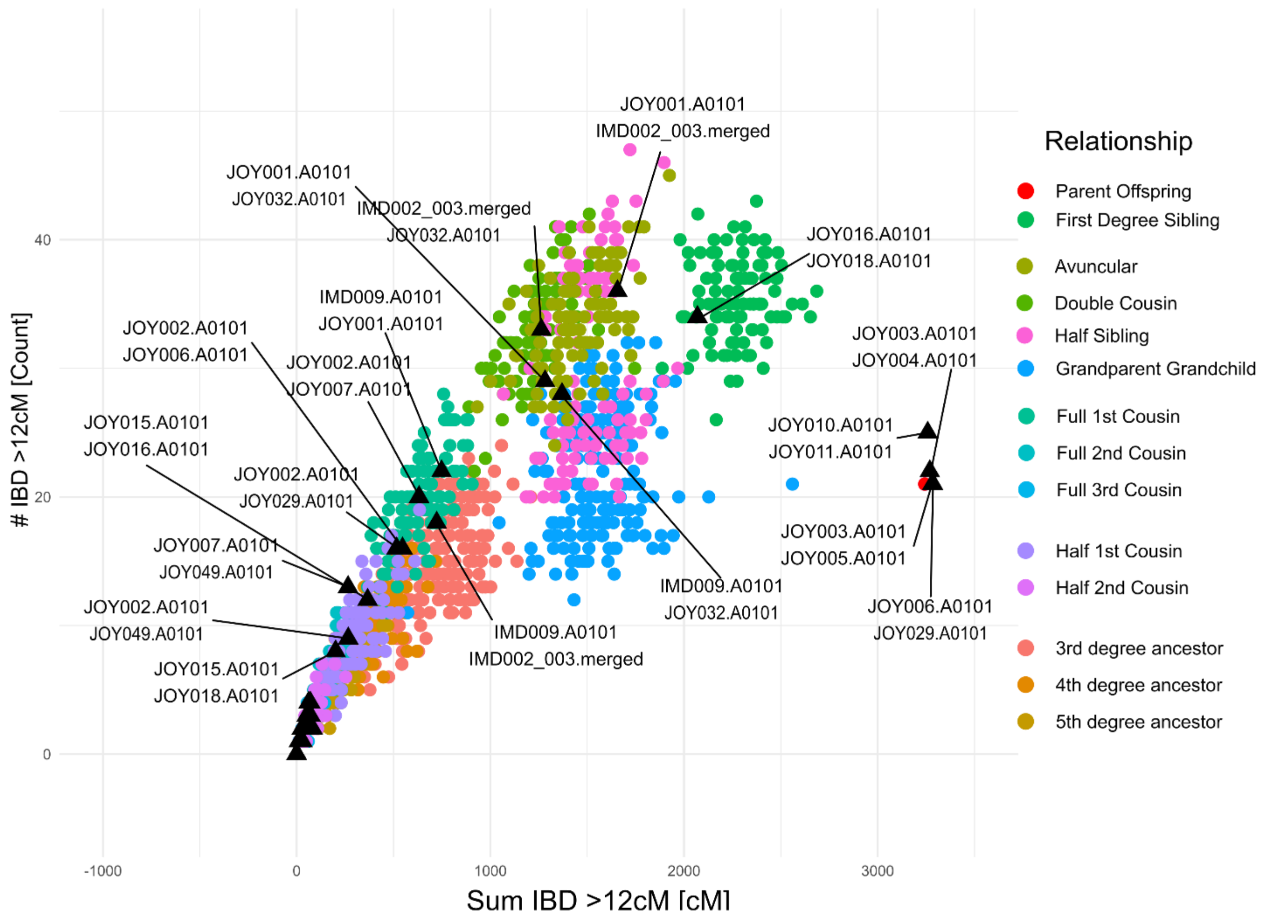

Figure S6. Distribution of IBD segments with different degrees of kinship. We plotted the total length of IBD segments longer than 12cM (Sum IBD >12cM) against the number of such segments (IBD >12cM) for individuals used for ancIBD analysis (black triangle). We compared the value with values from simulated data between different kinship relationships. 100 simulated data were used for each relationship.

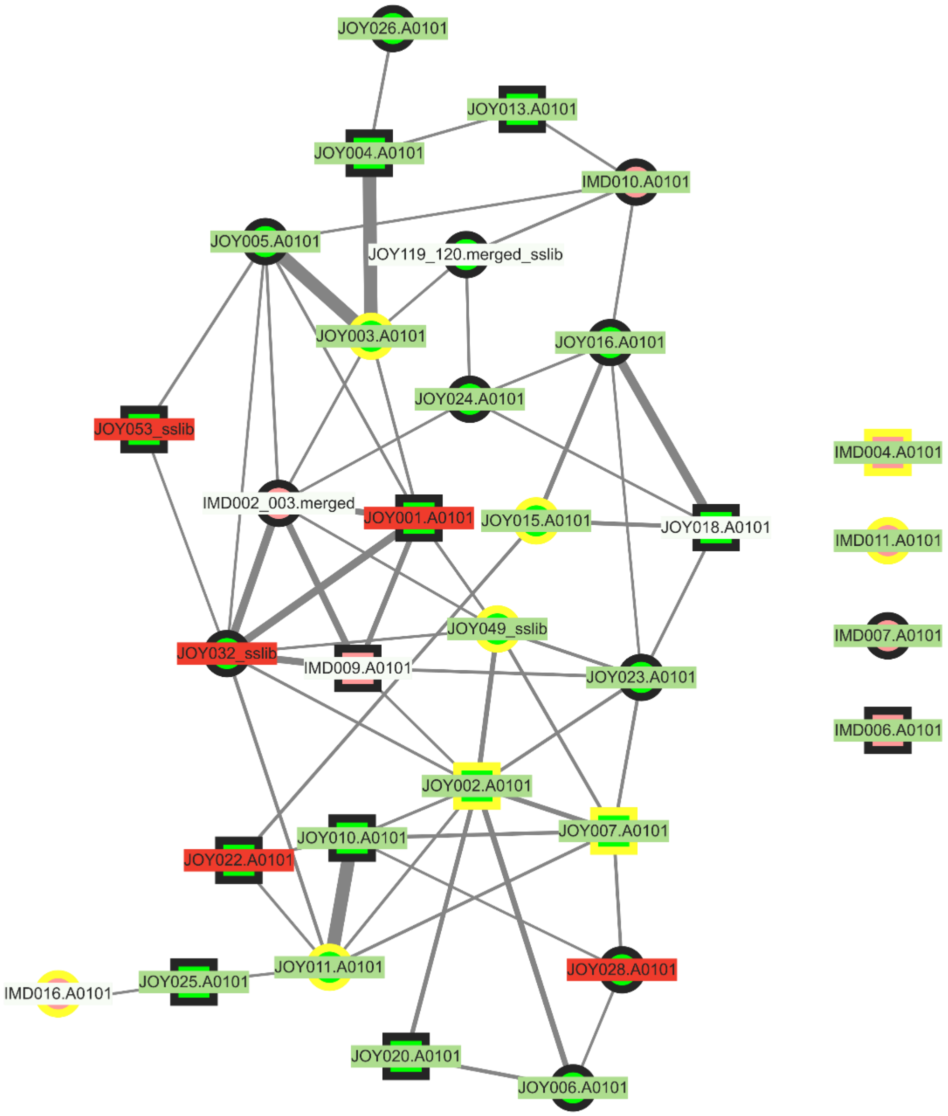

Figure S7. IBD network between Imdang-Joyeong individuals based on ancIBD results with individual labels Network visualization of IBD connections (edge) between individuals (node). The color of the node represents the burial site, while the color of the outline being yellow indicates the age of death under 20. The color of the label indicates burial status. Red indicates grave owners, green indicates sacrificed individuals, and white indicates individuals with unknown burial status. The width of the edges (strength) represents the max IBD length shared between the two individuals.

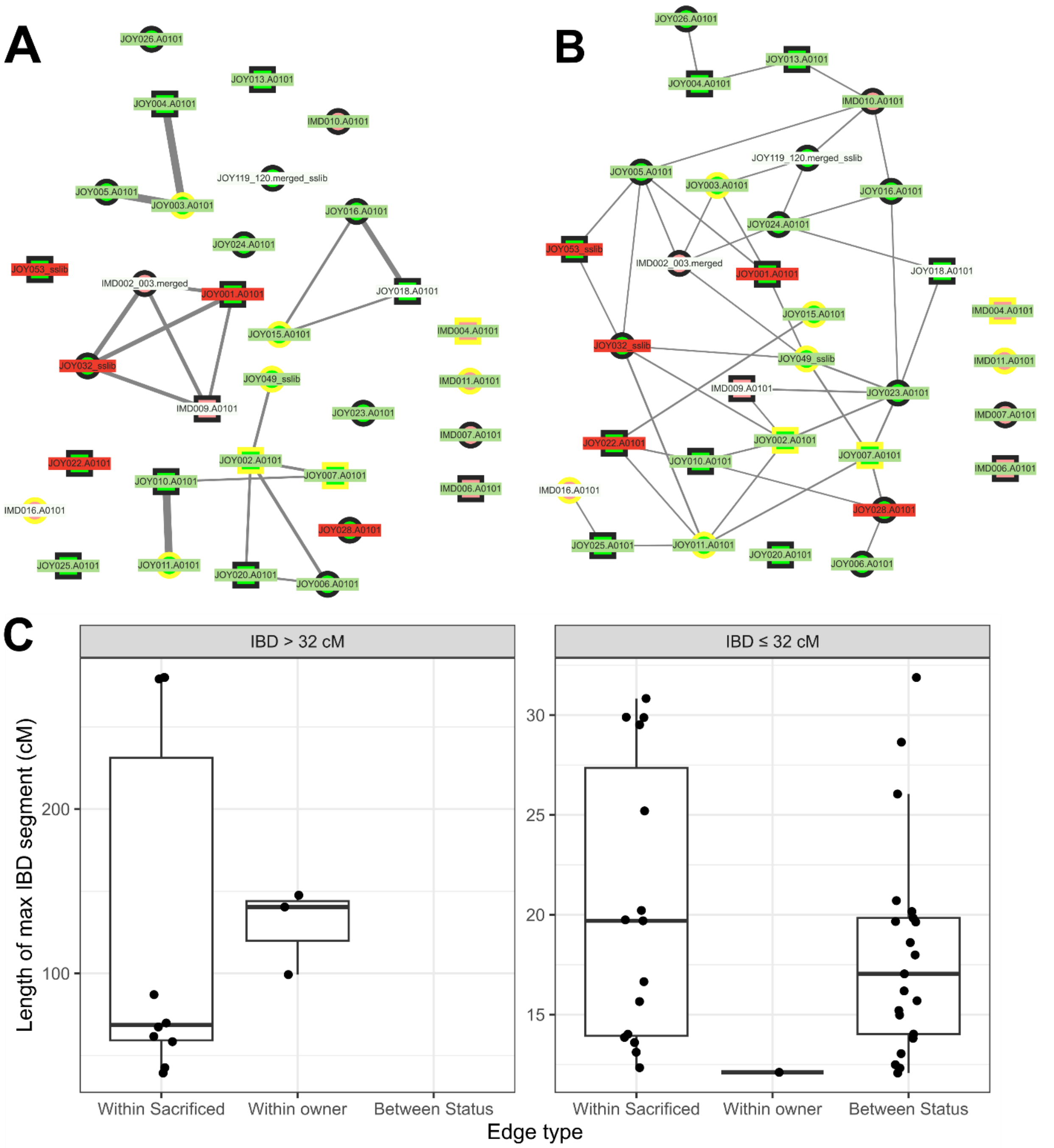

Figure S8. Different patterns of IBD sharing between Imdang-Joyeong grave owners and sacrificed individuals depending on IBD length class. A,B Same network visualization of IBD connections (edge) between individuals (node) as Figure S7. **A.** Network with edges with max IBD length larger than 32 cM. **B.** Network with edges with max IBD length between 12 – 32 cM. **C.** Boxplot of the distribution of max IBD segment length of all network edges, divided by whether the edge is between individuals of same or different status, faceted by the length of max IBD sharing longer or shorter than 32 cM.

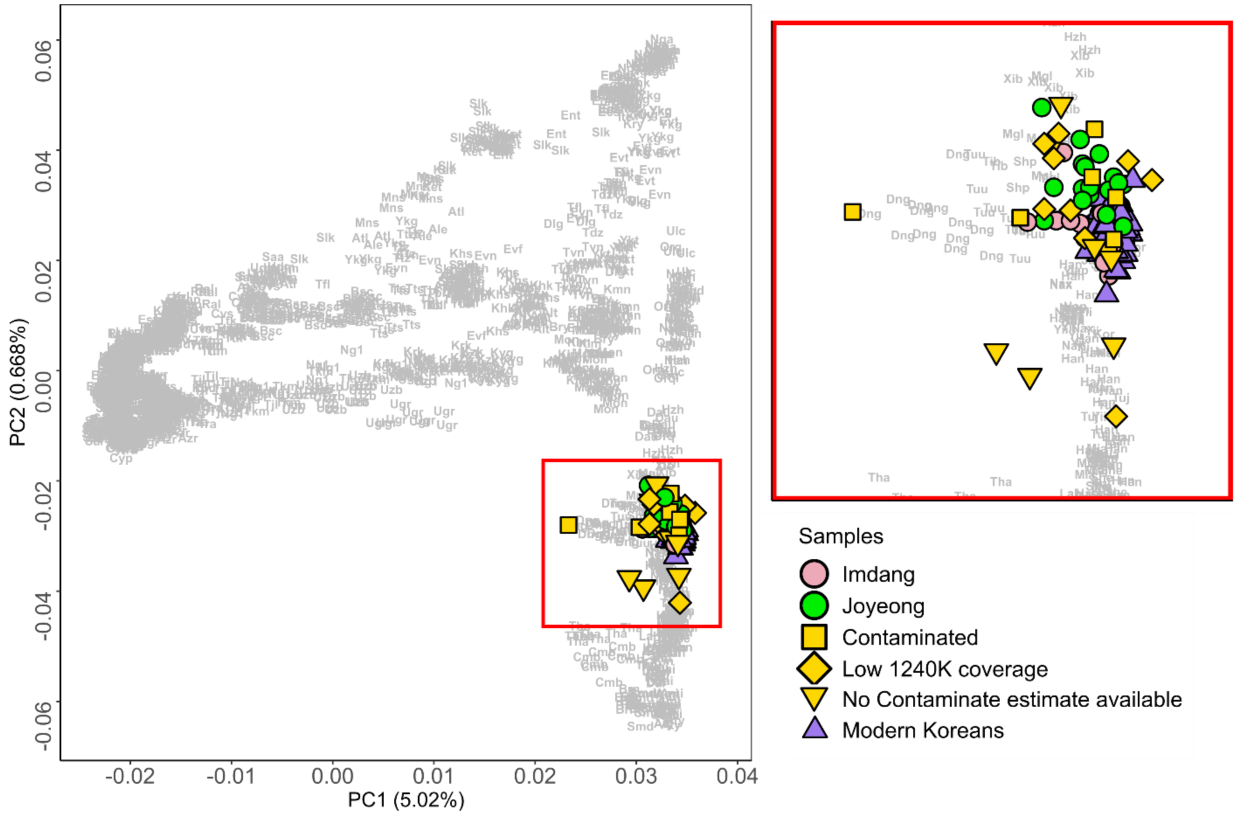

Figure S9. Eurasian PCA (principal component analysis) for Imdang-Joyeong ancient individuals and modern Koreans. Principal component analysis was conducted on 2,077 modern Eurasian humans. Ancient individuals and modern Koreans were projected onto the first two principal components. Ancient individuals are depicted as squares, while modern individuals are labelled as circles. Contaminated or individuals with low SNP coverage are flagged as yellow shapes. Grey letters are three letter abbreviations of the modern populations used for calculation of PCA. The variances explained by the first two PCs are shown in brackets.

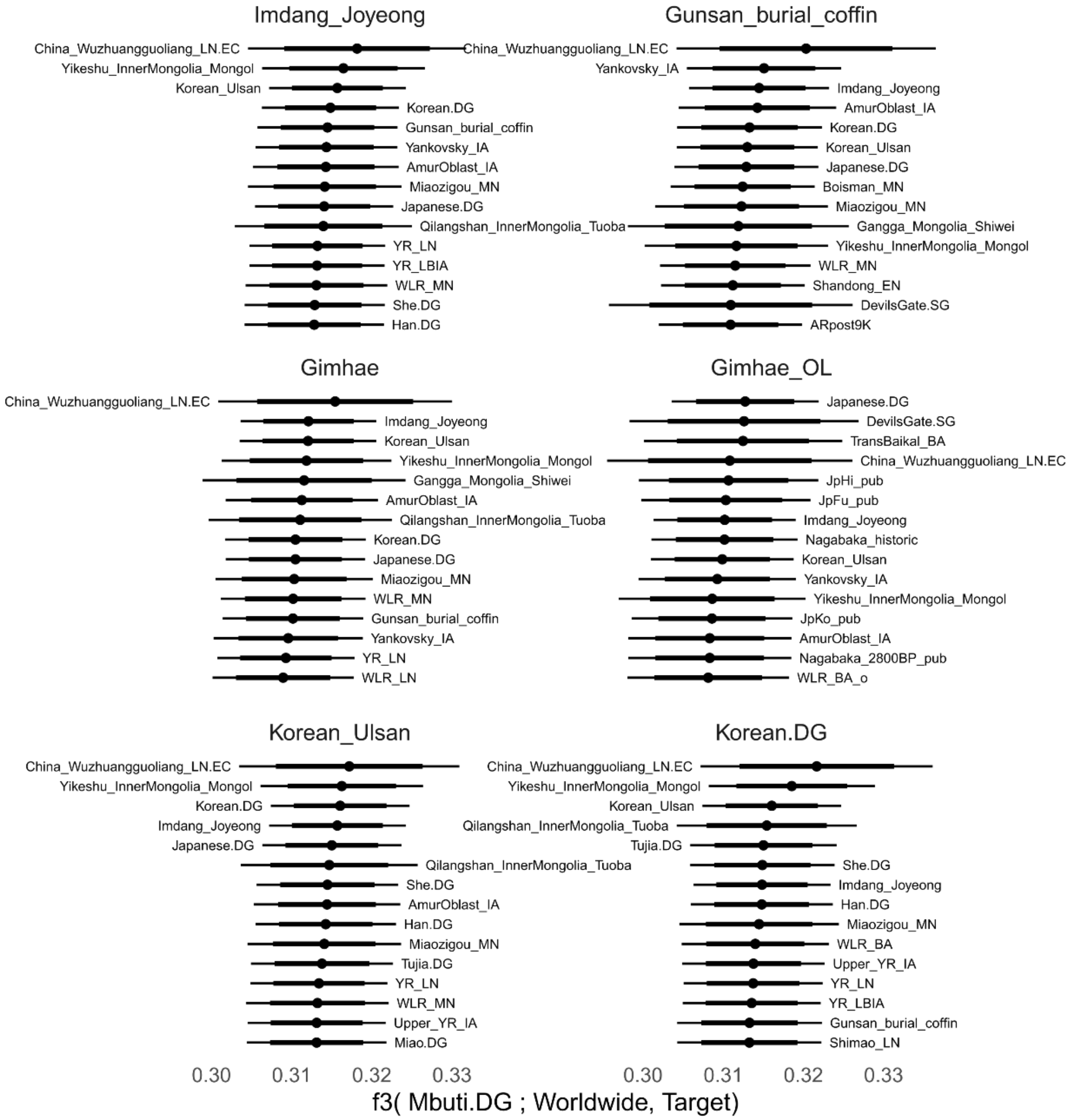

Figure S10. Outgroup *f*3 results of Three-Kingdoms period ancient and modern Koreans. We present the top ten highest estimates of the test *f*3(Mbuti; Korean, World-wide population) for each ancient Three-kingdoms period and modern Koreans. Population names are denoted next to the estimate value. Points represent the estimate value, thick lines represent 2 standard error (SE) intervals, whisker lines represent 3 SE intervals. SE was calculated by using 5 cM block jackknife resampling approach implemented in the admixtools2 R library package.

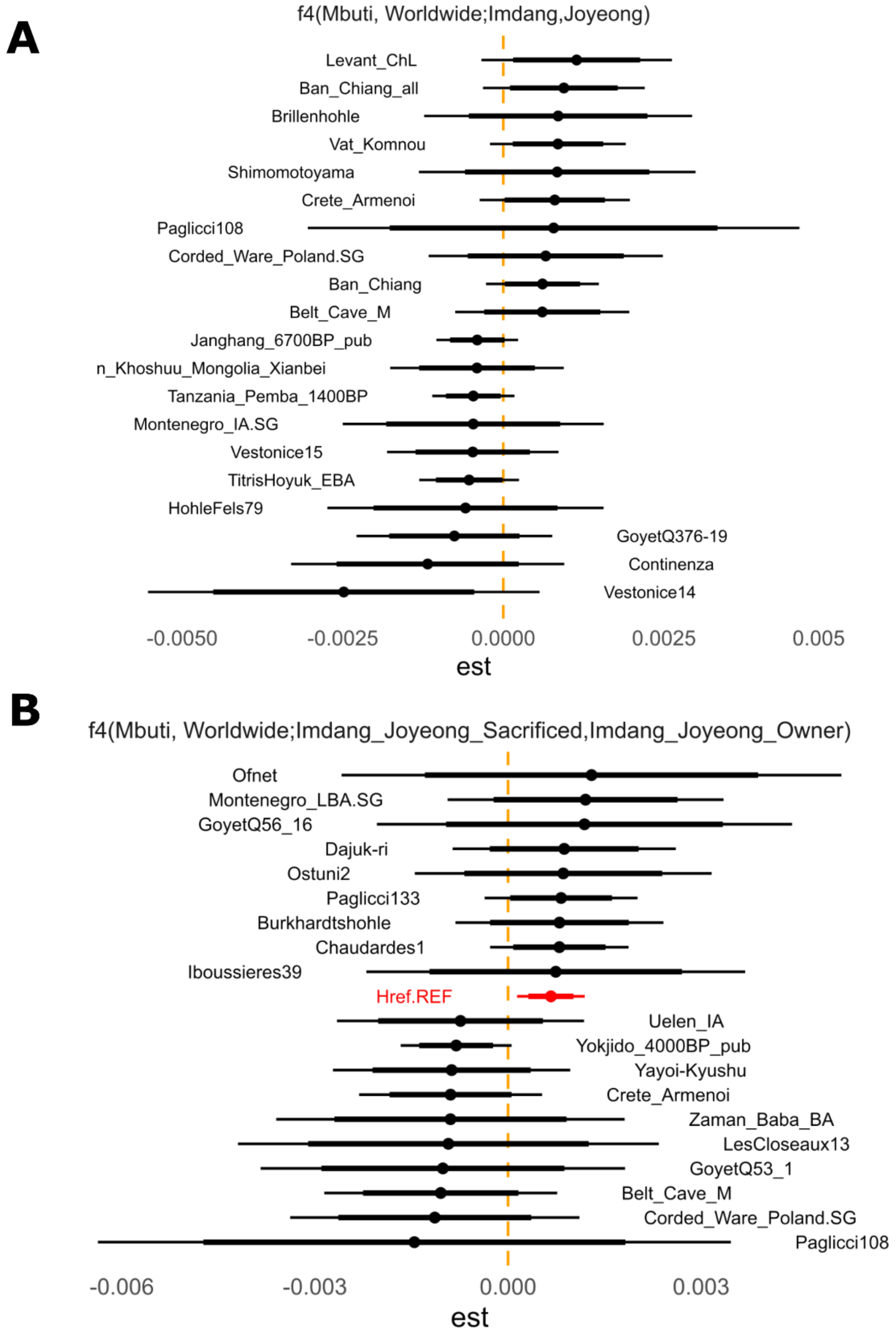

Figure S11. ***f*4 tests comparing the genetic affinity between burial site and buried status.** We present the test results of the top ten largest absolute values for each positive and negative sign of the test **A.** *f*4(Mbuti, Worldwide populations; Imdang site, Joyeong site) and **B.** *f*4(Mbuti, Worldwide populations; Imdang-Joyeong sacrificed group, Imdang-Joyeong grave owner). Worldwide population names are denoted next to the estimate value. Points represent the estimate value, thick lines represent 2 standard error (SE) intervals, whisker lines represent 3 SE intervals. SE was calculated by using 5 cM block jackknife resampling approach implemented in the admixtools2 R library package.

Supplementary Data files

The following data are provided as a spreadsheet file:

Data S1. Summary of Imdang-Joyeong ancient individuals analyzed in this study.
We provide the bioarchaeological metadata, sequencing summary, and analysis labels for each library. Endogenous DNA corresponds to the proportion of trimmed reads mapped to the human reference genome. 5' C>T Deamination refers to the proportion of C>T misincorporations of the first position of 5' end of reads. X Chromosome contamination corresponds to the nuclear contamination estimates and associated standard error, calculated from the contamination estimation module in ANGSD using the Method of Moment (MoM) estimator of Method 1. Schmutzi mtDNA contamination refers to the mitochondrial contamination estimate with upper and lower bounds from Schmutzi. 1240K capture depth coverage refers to the mean depth of reads covering 1240K SNP sites.

Data S2. Information on duplicate libraries.
We provide information about libraries from the same genetic individual that were merged during our analysis. Libraries with same UDG treatment strategies were merged through merging BAM files, while libraries with different UDG treatments were merged during 1240K SNP genotyping. 1240K Depth Coverage refers to the mean number of reads covering 1240K SNP sites after merging BAM files. mtDNA contamination refers to the mitochondrial contamination estimate with upper and lower bounds from Schmutzi.

Data S3. List of previously published modern and ancient populations used in this study.
We present the number of individuals per population, the publication, and the corresponding sample IDs for each population.

Data S4. Relatedness between Imdang and Joyeong individuals.
We present the likelihood values inferred by KIN and the pairwise mismatch rate (PMR) between Imdang-Joyeong ancients. Red cells indicate the max likelihood value. Orange cells indicate the range of likelihood values within 1 of the max likelihood value. Green cells indicate the range of likelihood values between 2 and 1 difference of the max likelihood value. We leave comments on relationships that were filtered out or reconsidered.

Data S5. Statistical values of network analyses.
We provide the p-values and the statistic value of our network analyses where we compare the empirical distribution of degree and strength of connectivity between adult males and females using two different test statistics.

Data S6. Outgroup *f*3 statistics.
We calculate *f*3(Mbuti; World-wide Population, Ancient or Modern Korean) to measure the shared genetic affinity between Koreans and world-wide populations. Standard errors (se) are calculated by 5 cM block jackknifing resampling approach implemented in the admixtools2 R package.

Data S7. Comparing ancestry between sacrificed and owner indivduals using *f*4 statistics.
To detect differences in ancestry between sacrificed individuals and grave owners within the Imdang-Joyeong burial complex, we calculated *f*4(Mbuti, World-wide Populations; Sacrificed, Grave owner). Standard errors (SE) were calculated by 5 cM block jackknife resampling approach in the admixtools2 R package.

Data S8. Comparing ancestry between Imdang and Joyeong indivduals using *f*4 statistics.
To detect differences in ancestry between individuals buried at the Imdang site compared to individuals buried at the Joyeong site within the Imdang-Joyeong burial complex, we calculated *f*4(Mbuti, World-wide Populations; Imdang, Joyeong). Standard errors (SE) were calculated by 5 cM block jackknife resampling approach in the admixtools2 R package.

Data S9. qpAdm modelling results.
To model the ancestry proportions of ancient and modern East Asian populations, we set WLR_BA, Xitoucun, and Jomon_Ikawasu as a possible admixture source. P-values higher than 0.01 are highlighted in dark grey. We present standard errors along with the weight of coefficients, which were calculated by 5cM block jackknife resampling approach in the admixtools2 R package. We highlight the most parsimonious model with a non-significant P-value with the fewest number of coefficients in yellow.

Data S10. Relatedness between published ancient Koreans with Imdang-Joyeong ancients.
We present the likelihood values inferred by KIN and the pairwise mismatch rate (PMR) between published ancient Koreans with Imdang-Joyeong ancients. Red cells indicate the max likelihood value. Orange cells indicate the range of likelihood values within 1 of the max likelihood value. Green cells indicate the range of likelihood values between 2 and 1 difference of the max likelihood value. We leave comments on relationships that were filtered out or reconsidered. Individual AKG_10204 from Gimhae city was filtered out due to low 1240K SNP depth coverage (0.00841x).

REFERENCES

1. M. Rivollat, A. B. Rohrlach, H. Ringbauer, A. Childebayeva, F. Mendisco, R. Barquera, A. Szolek, M. Le Roy, H. Colleran, J. Tuke, F. Aron, M.-H. Pemonge, E. Späth, P. Télouk, L. Rey, G. Goude, V. Balter, J. Krause, S. Rottier, M.-F. Deguilloux, W. Haak, Extensive pedigrees reveal the social organization of a Neolithic community. *Nature* **620**, 600–606 (2023).

2. C. Fowler, I. Olalde, V. Cummings, I. Armit, L. Büster, S. Cuthbert, N. Rohland, O. Cheronet, R. Pinhasi, D. Reich, A high-resolution picture of kinship practices in an Early Neolithic tomb. *Nature* **601**, 584–587 (2022).

3. S. Penske, M. Küßner, A. B. Rohrlach, C. Knipper, J. Nováček, A. Childebayeva, J. Krause, W. Haak, Kinship practices at the early bronze age site of Leubingen in Central Germany. *Sci. Rep.* **14**, 3871 (2024).

4. V. Villalba-Mouco, C. Oliart, C. Rihuete-Herrada, A. B. Rohrlach, M. I. Fregeiro, A. Childebayeva, H. Ringbauer, I. Olalde, E. Celdrán Beltrán, C. Puello-Mora, M. Valério, J. Krause, V. Lull, R. Micó, R. Risch, W. Haak, Kinship practices in the early state El Argar society from Bronze Age Iberia. *Sci. Rep.* **12**, 22415 (2022).

5. A. Mittnik, K. Massy, C. Knipper, F. Wittenborn, R. Friedrich, S. Pfrengle, M. Burri, N. Carlichi-Witjes, H. Deeg, A. Furtwängler, M. Harbeck, K. von Heyking, C. Kociumaka, I. Kucukkalipci, S. Lindauer, S. Metz, A. Staskiewicz, A. Thiel, J. Wahl, W. Haak, E. Pernicka, S. Schiffels, P. W. Stockhammer, J. Krause, Kinship-based social inequality in Bronze Age Europe. *Science* **366**, 731–734 (2019).

6. G. A. Gnecchi-Ruscone, Z. Rácz, L. Samu, T. Szeniczey, N. Faragó, C. Knipper, R. Friedrich, D. Zlámalová, L. Traverso, S. Liccardo, S. Wabnitz, D. Popli, K. Wang, R. Radzeviciute, B. Gulyás, I. Koncz, C. Balogh, G. M. Lezsák, V. Mácsai, M. M. E. Bunbury, O. Spekker, P. le Roux, A. Szécsényi-Nagy, B. G. Mende, H. Colleran, T. Hajdu, P. Geary, W. Pohl, T. Vida, J. Krause, Z. Hofmanová, Network of large pedigrees reveals social practices of Avar communities. *Nature* **629**, 376–383 (2024).

7. D. J. Kennett, S. Plog, R. J. George, B. J. Culleton, A. S. Watson, P. Skoglund, N. Rohland, S. Mallick, K. Stewardson, L. Kistler, S. A. LeBlanc, P. M. Whiteley, D. Reich, G. H. Perry,

Archaeogenomic evidence reveals prehistoric matrilineal dynasty. *Nat. Commun.* **8**, 14115 (2017).

8. J. Wang, S. Yan, Z. Li, J. Zan, Y. Zhao, J. Zhao, K. Chen, X. Wang, T. Ji, C. Zhang, T. Yang, T. Zhang, R. Qiao, M. Guo, Z. Rao, J. Zhang, G. Wang, Z. Ran, C. Duan, F. Zhang, Y. Song, X. Wu, R. Mace, B. Sun, Y. Pang, Y. Huang, H. Zhang, C. Ning, Ancient DNA reveals a two-clanned matrilineal community in Neolithic China. *Nature* **643**, 1304–1311 (2025).

9. E. Yüncü, A. K. Doğu, D. Kaptan, M. S. Kılıç, C. Mazzucato, M. N. Güler, E. Eker, B. Katırcıoğlu, M. Chyleński, K. B. Vural, E. Sağlıcan, G. Atağ, D. Bozkurt, J. Pearson, A. Sevkar, N. E. Altınışık, M. Milella, C. Karamurat, Ş. Aktürk, E. D. Yurttaş, N. Yıldız, D. Koptekin, S. Yorulmaz, D. D. Kazancı, A. Aydoğan, K. Gürün, E. M. J. Schotsmans, J. Anvari, E. Rosenstock, J. Byrnes, P. F. Biehl, D. Orton, V. K. Lagerholm, H. C. Gemici, M. Vasic, A. Marciniak, Ç. Atakuman, Y. S. Erdal, E. Kırdök, M. Pilloud, C. S. Larsen, S. D. Haddow, A. Götherström, C. J. Knüsel, F. Özer, I. Hodder, M. Somel, Female lineages and changing kinship patterns in Neolithic Çatalhöyük. *Science* **388**, eadr2915 (2025).

10. H. Ringbauer, J. Novembre, M. Steinrücken, Parental relatedness through time revealed by runs of homozygosity in ancient DNA. *Nat. Commun.* **12**, 5425 (2021).

11. E. Skourtanioti, H. Ringbauer, G. A. Gnecchi-Ruscone, R. A. Bianco, M. Burri, C. Freund, A. Furtwängler, N. F. Gomes Martins, F. Knolle, G. U. Neumann, A. Tiliakou, A. Agelarakis, M. Andreadaki-Vlazaki, P. Betancourt, B. P. Hallager, O. A. Jones, O. Kakavogianni, A. Kanta, P. Karkanas, E. Kataki, K. Kissas, R. Koehl, L. Kvapil, J. Maran, P. J. P. McGeorge, A. Papadimitriou, A. Papathanasiou, L. Papazoglou-Manioudaki, K. Paschalidis, N. Polychronakou-Sgouritsa, S. Preve, E.-A. Prevedorou, G. Price, E. Protopapadaki, T. Schmidt-Schultz, M. Schultz, K. Shelton, M. H. Wiener, J. Krause, C. Jeong, P. W. Stockhammer, Ancient DNA reveals admixture history and endogamy in the prehistoric Aegean. *Nat. Ecol. Evol.* **7**, 290–303 (2023).

12. C. Ning, F. Zhang, Y. Cao, L. Qin, M. J. Hudson, S. Gao, P. Ma, W. Li, S. Zhu, C. Li, T. Li, Y. Xu, C. Li, M. Robbeets, H. Zhang, Y. Cui, Ancient genome analyses shed light on kinship organization and mating practice of Late Neolithic society in China. *iScience* **24**, 103352 (2021).

13. R. Song-Nai, C. Mong-Lyong, Emergence of complex society in prehistoric Korea. *J. World Prehist.* **6**, 51–95 (1992).

14. M. Conte, J. Kim, An economy of human sacrifice: The practice of sunjang in an ancient state of Korea. *J. Anthropol. Archaeol.* **44**, 14–30 (2016).

15. D.-w. Kim, A study on the characteristics of buried person and The culture of sacrificial burial of Ancient tombs in Im-dang. *Sogang journal of early Korean history* **20**, 147–183 (2015).

16. K. Choy, H. Y. Yun, S. H. Kim, S. Jung, B. T. Fuller, D. W. Kim, Isotopic investigation of skeletal remains at the Imdang tombs reveals high consumption of game birds and social stratification in ancient Korea. *Sci. Rep.* **11**, 22551 (2021).

17. G. M. Schwartz, The archaeological study of sacrifice. *Ann. Rev. Anthropol.* **46**, 223–240 (2017).

18. A. Baadsgaard, J. Monge, R. L. Zettler, in Sacred Killing, P. Anne, M. S. Glenn, Eds. (Penn State Univ. Press, 2012), pp. 125–158.

19. R. Campbell, in Sacred Killing, P. Anne, M. S. Glenn, Eds. (Penn State Univ. Press, 2012), pp. 305–324.

20. M. Winkelman, Aztec human sacrifice: Cross-cultural assessments of the ecological hypothesis. *Ethnology* **37**, 285–298 (1998).

21. M. Harner, The enigma of aztec sacrifice. *Nat. Hist.* **86**, 46–51 (1977).

22. A. Porter, Divine Power and political aspiration in third millennium Mesopotamia and beyond, in *The archaeology of violence: Interdisciplinary approaches* (State University of New York Press, 2012), pp. 185–202.

23. T. Besom, *Inka Human Sacrifice and Mountain Worship: Strategies For Empire Unification* (UNM Press, 2013).

24. B. G. Trigger, *Understanding Early Civilizations: A Comparative Study* (Cambridge Univ. Press, 2014).

25. "The life and death of a 16 year old woman 1500 years ago," (Gaya National Research Institute of Cultural Heritage, Gaya National Research Institute of Cultural Heritage, 2009) (in Korean).

26. B. Kim, *The History of the Three Kingdoms (Samguk Sagi)* (Hangilsa Publishing Co., 2000) (in Korean).

27. Y. Kim, The Ancient Tombs and the Circumference of the Silla's Capital (Hakyeon Munhwasa, Seoul, 2009) (in Korean).

28. S. Young-Kyo, King Jijeung's funeral law and Silla's monogamy. *Hist. World* **53**, 91–116 (2018). (In Korean).

29. K. R. Lee, The Succession to the throne of king Chicheung's lineage and role of park clan in the early silla dynasty (The Journal of the Institute for East Asian Studies Sogang University, 1990), pp. 67–104 (in Korean).

30. K. Soo Tae, Changes in Marriage Rules of the Koguryo Royal family: The levirate revisited (The Journal of Korean Ancient History, 2005), pp. 85–111 (in Korean).

31. D.-N. Lee, C. L. Jeon, J. Kang, M. Burri, J. Krause, E. J. Woo, C. Jeong, Genomic detection of a secondary family burial in a single jar coffin in early Medieval Korea. *Am. J. Biol. Anthropol.* **179**, 585–597 (2022).

32. P. Gelabert, A. Blazyte, Y. Chang, D. M. Fernandes, S. Jeon, J. G. Hong, J. Yoon, Y. Ko, V. Oberreiter, O. Cheronet, K. T. Özdoğan, S. Sawyer, S. Yang, E. M. Greytak, H. Choi, J. Kim, J.-I. Kim, C. Jeong, K. Bae, J. Bhak, R. Pinhasi, Northeastern Asian and Jomon-related genetic structure in the Three Kingdoms period of Gimhae, Korea. *Curr. Biol.* **32**, 3232–3244.e6 (2022).

33. M. Robbeets, R. Bouckaert, M. Conte, A. Savelyev, T. Li, D.-I. An, K.-i. Shinoda, Y. Cui, T. Kawashima, G. Kim, J. Uchiyama, J. Dolińska, S. Oskolskaya, K.-Y. Yamano, N. Seguchi, H.

Tomita, H. Takamiya, H. Kanzawa-Kiriyama, H. Oota, H. Ishida, R. Kimura, T. Sato, J.-H. Kim, B. Deng, R. Bjørn, S. Rhee, K.-D. Ahn, I. Gruntov, O. Mazo, J. R. Bentley, R. Fernandes, P. Roberts, I. R. Bausch, L. Gilaizeau, M. Yoneda, M. Kugai, R. A. Bianco, F. Zhang, M. Himmel, M. J. Hudson, C. Ning, Triangulation supports agricultural spread of the Transeurasian languages. *Nature* **599**, 616–621 (2021).

34. D.-w. Kim, *The Excavation of Imdang and the World of Archaeology* (Yeungnam University Museum, 2021) (in Korean).

35. D.-w. Kim, "A study on the funeral system of ancient tombs at the Im-dang site," thesis, Yeungnam University, Gyeongsangbuk-do, South Korea (2014) (in Korean).

36. Y. Kim, K. Kim, S. Park, J. Kang, Y. Kim, D. Kim, J. Kim, G. City, D. Sahakhoe, *Gyeongsan in Ancient Korean History* (Daegu Sahakhoe, Gyeongsan City, 2008), pp. 274 (in Korean).

37. Y. s. Kim, *Silla's Ancient Tombs and Regional Communities*. [Chun-chu-gak (□□□), 1998], pp. 485 (in Korean).

38. J. Lee, D. Ha, S. Pak, E. Woo, C. Lee, D. Kim, J. Kim, Y. Han, The relations of the dead: Identifying the relationship of individuals buried at Imdang, Gyeongsan, through the analysis of mitochondrial DNA from human skeletal remains interred in large mounded tombs. *J. Korean Archaeol. Soc.* **68**, 128–155 (2008).

39. D. Ha, A study on the status of the victims of the Silla sacrificial burials at Imdang. *J. Korean Archaeol. Soc.* **79**, 175–204 (2011).

40. Q. Fu, M. Hajdinjak, O. T. Moldovan, S. Constantin, S. Mallick, P. Skoglund, N. Patterson, N. Rohland, I. Lazaridis, B. Nickel, B. Viola, K. Prüfer, M. Meyer, J. Kelso, D. Reich, S. Pääbo, An early modern human from Romania with a recent Neanderthal ancestor. *Nature* **524**, 216–219 (2015).

41. I. Mathieson, I. Lazaridis, N. Rohland, S. Mallick, N. Patterson, S. A. Roodenberg, E. Harney, K. Stewardson, D. Fernandes, M. Novak, K. Sirak, C. Gamba, E. R. Jones, B. Llamas, S. Dryomov, J. Pickrell, J. L. Arsuaga, J. M. B. de Castro, E. Carbonell, F. Gerritsen,

A. Khokhlov, P. Kuznetsov, M. Lozano, H. Meller, O. Mochalov, V. Moiseyev, M. A. R. Guerra, J. Roodenberg, J. M. Vergès, J. Krause, A. Cooper, K. W. Alt, D. Brown, D. Anthony, C. Lalueza-Fox, W. Haak, R. Pinhasi, D. Reich, Genome-wide patterns of selection in 230 ancient Eurasians. *Nature* **528**, 499–503 (2015).

42. W. Haak, I. Lazaridis, N. Patterson, N. Rohland, S. Mallick, B. Llamas, G. Brandt, S. Nordenfelt, E. Harney, K. Stewardson, Q. Fu, A. Mittnik, E. Bánffy, C. Economou, M. Francken, S. Friederich, R. G. Pena, F. Hallgren, V. Khartanovich, A. Khokhlov, M. Kunst, P. Kuznetsov, H. Meller, O. Mochalov, V. Moiseyev, N. Nicklisch, S. L. Pichler, R. Risch, M. A. Rojo Guerra, C. Roth, A. Szécsényi-Nagy, J. Wahl, M. Meyer, J. Krause, D. Brown, D. Anthony, A. Cooper, K. W. Alt, D. Reich, Massive migration from the steppe was a source for Indo-European languages in Europe. *Nature* **522**, 207–211 (2015).

43. D. Popli, S. Peyrégne, B. M. Peter, KIN: A method to infer relatedness from low-coverage ancient DNA. *Genome Biol.* **24**, 10 (2023).

44. S. Liccardo, S. Wabnitz, Family Matters: The Levirate Marriage as a Nomadic Custom in Medieval Eurasia. *Medieval Worlds* **20**, 191–227 (2024).

45. International Society of Genetic Genealogy, Y-DNA Haplogroup Tree, Version: V.9.129. International Society of Genetic Genealogy (2014); https://isogg.org/tree/2014/ISOGG_HapgrpO14.html [accessed 6 April 2025].

46. International Society of Genetic Genealogy, Y-DNA Haplogroup Tree, Version: V.10.120. International Society of Genetic Geneology (2015); https://isogg.org/tree/2015/ISOGG_HapgrpO15.html [accessed 6 April 2025].

47. J. Kim, S. Jeon, J.-P. Choi, A. Blazyte, Y. Jeon, J.-I. Kim, J. Ohashi, K. Tokunaga, S. Sugano, S. Fucharoen, F. Al-Mulla, J. Bhak, The origin and composition of korean ethnicity analyzed by ancient and present-day genome sequences. *Genome Biol. Evol.* **12**, 553–565 (2020).

48. M. J. Park, H. Y. Lee, W. I. Yang, K.-J. Shin, Understanding the Y chromosome variation in Korea—Relevance of combined haplogroup and haplotype analyses. *Int. J. Leg. Med.* **126**, 589–599 (2012).

49. A. H. Zhang, H. Y. Lee, S. B. Seo, H. J. Lee, H. X. Jin, S. H. Cho, S. H. Lyoo, K. H. Kim, J. W. Lee, S. D. Lee, Y Haplogroup distribution in korean and other populations. *Korean J. Leg. Med.* **36**, 34–44 (2012).

50. N. Patterson, A. L. Price, D. Reich, Population structure and eigenanalysis. *PLOS Genet.* **2**, e190 (2006).

51. A. H. Bittles, M. L. Black, Consanguinity, human evolution, and complex diseases. *Proc. Natl. Acad. Sci. U.S.A.* **107**, 1779–1786 (2010).

52. F. C. Ceballos, P. K. Joshi, D. W. Clark, M. Ramsay, J. F. Wilson, Runs of homozygosity: Windows into population history and trait architecture. *Nat. Rev. Genet.* **19**, 220–234 (2018).

53. J.-g. Noh, T.-s. Kim, S.-g. Kim, H.-s. Lee, Y.-s. Lee, H.-s. Kang, Y.-s. Kim, C.-h. Lee, J.-h. Shin, *Gaya Tumuli in the Gyeongbuk Region and World Cultural Heritage*. (Academia Koreana, Keimyung University, 2012) (in Korean).

54. M. Meyer, M. Kircher, Illumina sequencing library preparation for highly multiplexed target capture and sequencing. *Cold Spring Harbor Protocols* **2010**, pdb.prot5448 (2010).

55. J. Dabney, M. Meyer, "Extraction of highly degraded dna from ancient bones and teeth," in *Ancient DNA: Methods and Protocols*, B. Shapiro, A. Barlow, P. D. Heintzman, M. Hofreiter, L. A. Paijmans, A. E. R. Soares, Eds. (Springer, 2019), pp. 25–29.

56. N. Rohland, E. Harney, S. Mallick, S. Nordenfelt, D. Reich, Partial uracil–DNA–glycosylase treatment for screening of ancient DNA. *Philos. Trans. R. Soc. B. Biol. Sci.* **370**, 20130624 (2015).

57. M. Schubert, S. Lindgreen, L. Orlando, AdapterRemoval v2: Rapid adapter trimming, identification, and read merging. *BMC. Res. Notes* **9**, 88 (2016).

58. M. Meyer, J.-L. Arsuaga, C. de Filippo, S. Nagel, A. Aximu-Petri, B. Nickel, I. Martínez, A. Gracia, J. M. B. de Castro, E. Carbonell, B. Viola, J. Kelso, K. Prüfer, S. Pääbo, Nuclear DNA sequences from the Middle Pleistocene Sima de los Huesos hominins. *Nature* **531**, 504–507 (2016).

59. H. Li, R. Durbin, Fast and accurate short read alignment with Burrows–Wheeler transform. *Bioinformatics* **25**, 1754–1760 (2009).

60. A. Peltzer, G. Jäger, A. Herbig, A. Seitz, C. Kniep, J. Krause, K. Nieselt, EAGER: Efficient ancient genome reconstruction. *Genome Biol.* **17**, 60 (2016).

61. H. Li, B. Handsaker, A. Wysoker, T. Fennell, J. Ruan, N. Homer, G. Marth, G. Abecasis, R. Durbin, 1000 Genome Project Data Processing Subgroup, The sequence alignment/map format and SAMtools. *Bioinformatics* **25**, 2078–2079 (2009).

62. H. Jónsson, A. Ginolhac, M. Schubert, P. L. F. Johnson, L. Orlando, mapDamage2.0: Fast approximate Bayesian estimates of ancient DNA damage parameters. *Bioinformatics* **29**, 1682–1684 (2013).

63. T. S. Korneliussen, A. Albrechtsen, R. Nielsen, ANGSD: Analysis of next generation sequencing data. *BMC Bioinformatics* **15**, 356 (2014).

64. G. Renaud, V. Slon, A. T. Duggan, J. Kelso, Schmutzi: Estimation of contamination and endogenous mitochondrial consensus calling for ancient DNA. *Genome Biol.* **16**, 224 (2015).

65. G. Jun, M. K. Wing, G. R. Abecasis, H. M. Kang, An efficient and scalable analysis framework for variant extraction and refinement from population-scale DNA sequence data. *Genome Res.* **25**, 918–925 (2015).

66. J. Kim, J. A. Weber, S. Jho, J. Jang, J. Jun, Y. S. Cho, H.-M. Kim, H. Kim, Y. Kim, O. Chung, C. G. Kim, H. Lee, B. C. Kim, K. Han, I. Koh, K. S. Chae, S. Lee, J. S. Edwards, J. Bhak, KoVariome: Korean National Standard Reference Variome database of whole genomes with comprehensive SNV, indel, CNV, and SV analyses. *Sci. Rep.* **8**, 5677 (2018).

67. H. Weissensteiner, D. Pacher, A. Kloss-Brandstätter, L. Forer, G. Specht, H.-J. Bandelt, F. Kronenberg, A. Salas, S. Schönherr, HaploGrep 2: Mitochondrial haplogroup classification in the era of high-throughput sequencing. *Nucleic Acids Res.* **44**, W58–W63 (2016).

68. International Society of Genetic Genealogy, Y-DNA Haplogroup Tree, Version: V.15.73, (2020); https://isogg.org/tree/ [accessed 6 April 2025].

69. G. D. Poznik, Identifying Y-chromosome haplogroups in arbitrarily large samples of sequenced or genotyped men. bioRxiv 088716 [Preprint] (2016); www.biorxiv.org/content/10.1101/088716v1.

70. H. Ringbauer, Y. Huang, A. Akbari, S. Mallick, I. Olalde, N. Patterson, D. Reich, Accurate detection of identity-by-descent segments in human ancient DNA. *Nat. Genet.* **56**, 143–151 (2024).

71. M. Caballero, D. N. Seidman, Y. Qiao, J. Sannerud, T. D. Dyer, D. M. Lehman, J. E. Curran, R. Duggirala, J. Blangero, S. Carmi, A. L. Williams, Crossover interference and sex-specific genetic maps shape identical by descent sharing in close relatives. *PLOS Genet.* **15**, e1007979 (2019).

72. C. Bhérer, C. L. Campbell, A. Auton, Refined genetic maps reveal sexual dimorphism in human meiotic recombination at multiple scales. *Nat. Commun.* **8**, 14994 (2017).

73. C. L. Campbell, N. A. Furlotte, N. Eriksson, D. Hinds, A. Auton, Escape from crossover interference increases with maternal age. *Nat. Commun.* **6**, 6260 (2015).

74. I. Lazaridis, D. Nadel, G. Rollefson, D. C. Merrett, N. Rohland, S. Mallick, D. Fernandes, M. Novak, B. Gamarra, K. Sirak, S. Connell, K. Stewardson, E. Harney, Q. Fu, G. Gonzalez-Fortes, E. R. Jones, S. A. Roodenberg, G. Lengyel, F. Bocquentin, B. Gasparian, J. M. Monge, M. Gregg, V. Eshed, A.-S. Mizrahi, C. Meiklejohn, F. Gerritsen, L. Bejenaru, M. Blüher, A. Campbell, G. Cavalleri, D. Comas, P. Froguel, E. Gilbert, S. M. Kerr, P. Kovacs, J. Krause, D. McGettigan, M. Merrigan, D. A. Merriwether, S. O'Reilly, M. B. Richards, O. Semino, M. Shamoon-Pour, G. Stefanescu, M. Stumvoll, A. Tönjes, A. Torroni, J. F. Wilson, L. Yengo, N. A. Hovhannisyan, N. Patterson, R. Pinhasi, D. Reich, Genomic insights into the origin of farming in the ancient Near East. *Nature* **536**, 419–424 (2016).

75. R. Maier, P. Flegontov, O. Flegontova, U. Işıldak, P. Changmai, D. Reich, On the limits of fitting complex models of population history to f-statistics. *eLife* **12**, e85492 (2023).

76. C. Jeong, O. Balanovsky, E. Lukianova, N. Kahbatkyzy, P. Flegontov, V. Zaporozhchenko, A. Immel, C.-C. Wang, O. Ixan, E. Khussainova, B. Bekmanov, V. Zaibert, M. Lavryashina, E.

Pocheshkhova, Y. Yusupov, A. Agdzhoyan, S. Koshel, A. Bukin, P. Nymadawa, S. Turdikulova, D. Dalimova, M. Churnosov, R. Skhalyakho, D. Daragan, Y. Bogunov, A. Bogunova, A. Shtrunov, N. Dubova, M. Zhabagin, L. Yepiskoposyan, V. Churakov, N. Pislegin, L. Damba, L. Saroyants, K. Dibirova, L. Atramentova, O. Utevska, E. Idrisov, E. Kamenshchikova, I. Evseeva, M. Metspalu, A. K. Outram, M. Robbeets, L. Djansugurova, E. Balanovska, S. Schiffels, W. Haak, D. Reich, J. Krause, The genetic history of admixture across inner Eurasia. *Nat. Ecol. Evol.* **3**, 966–976 (2019).

77. I. Lazaridis, N. Patterson, A. Mittnik, G. Renaud, S. Mallick, K. Kirsanow, P. H. Sudmant, J. G. Schraiber, S. Castellano, M. Lipson, B. Berger, C. Economou, R. Bollongino, Q. Fu, K. I. Bos, S. Nordenfelt, H. Li, C. de Filippo, K. Prüfer, S. Sawyer, C. Posth, W. Haak, F. Hallgren, E. Fornander, N. Rohland, D. Delsate, M. Francken, J.-M. Guinet, J. Wahl, G. Ayodo, H. A. Babiker, G. Bailliet, E. Balanovska, O. Balanovsky, R. Barrantes, G. Bedoya, H. Ben-Ami, J. Bene, F. Berrada, C. M. Bravi, F. Brisighelli, G. B. J. Busby, F. Cali, M. Churnosov, D. E. C. Cole, D. Corach, L. Damba, G. van Driem, S. Dryomov, J.-M. Dugoujon, S. A. Fedorova, I. Gallego Romero, M. Gubina, M. Hammer, B. M. Henn, T. Hervig, U. Hodoglugil, A. R. Jha, S. Karachanak-Yankova, R. Khusainova, E. Khusnutdinova, R. Kittles, T. Kivisild, W. Klitz, V. Kučinskas, A. Kushniarevich, L. Laredj, S. Litvinov, T. Loukidis, R. W. Mahley, B. Melegh, E. Metspalu, J. Molina, J. Mountain, K. Näkkäläjärvi, D. Nesheva, T. Nyambo, L. Osipova, J. Parik, F. Platonov, O. Posukh, V. Romano, F. Rothhammer, I. Rudan, R. Ruizbakiev, H. Sahakyan, A. Sajantila, A. Salas, E. B. Starikovskaya, A. Tarekegn, D. Toncheva, S. Turdikulova, I. Uktveryte, O. Utevska, R. Vasquez, M. Villena, M. Voevoda, C. A. Winkler, L. Yepiskoposyan, P. Zalloua, T. Zemunik, A. Cooper, C. Capelli, M. G. Thomas, A. Ruiz-Linares, S. A. Tishkoff, L. Singh, K. Thangaraj, R. Villems, D. Comas, R. Sukernik, M. Metspalu, M. Meyer, E. E. Eichler, J. Burger, M. Slatkin, S. Pääbo, J. Kelso, D. Reich, J. Krause, Ancient human genomes suggest three ancestral populations for present-day Europeans. *Nature* **513**, 409–413 (2014).

78. N. Patterson, P. Moorjani, Y. Luo, S. Mallick, N. Rohland, Y. Zhan, T. Genschoreck, T. Webster, D. Reich, Ancient admixture in human history. *Genetics* **192**, 1065–1093 (2012).

79. P. Qin, M. Stoneking, Denisovan ancestry in east eurasian and native american populations. *Mol. Biol. Evol.* **32**, 2665–2674 (2015).

80. P. Flegontov, N. E. Altınışık, P. Changmai, N. Rohland, S. Mallick, N. Adamski, D. A. Bolnick, N. Broomandkhoshbacht, F. Candilio, B. J. Culleton, O. Flegontova, T. M. Friesen, C. Jeong, T. K. Harper, D. Keating, D. J. Kennett, A. M. Kim, T. C. Lamnidis, A. M. Lawson, I. Olalde, J. Oppenheimer, B. A. Potter, J. Raff, R. A. Sattler, P. Skoglund, K. Stewardson, E. J. Vajda, S. Vasilyev, E. Veselovskaya, M. G. Hayes, D. H. O'Rourke, J. Krause, R. Pinhasi, D. Reich, S. Schiffels, Palaeo-Eskimo genetic ancestry and the peopling of Chukotka and North America. *Nature* **570**, 236–240 (2019).

81. S. Mallick, H. Li, M. Lipson, I. Mathieson, M. Gymrek, F. Racimo, M. Zhao, N. Chennagiri, S. Nordenfelt, A. Tandon, P. Skoglund, I. Lazaridis, S. Sankararaman, Q. Fu, N. Rohland, G. Renaud, Y. Erlich, T. Willems, C. Gallo, J. P. Spence, Y. S. Song, G. Poletti, F. Balloux, G. van Driem, P. de Knijff, I. G. Romero, A. R. Jha, D. M. Behar, C. M. Bravi, C. Capelli, T. Hervig, A. Moreno-Estrada, O. L. Posukh, E. Balanovska, O. Balanovsky, S. Karachanak-Yankova, H. Sahakyan, D. Toncheva, L. Yepiskoposyan, C. Tyler-Smith, Y. Xue, M. S. Abdullah, A. Ruiz-Linares, C. M. Beall, A. Di Rienzo, C. Jeong, E. B. Starikovskaya, E. Metspalu, J. Parik, R. Villems, B. M. Henn, U. Hodoglugil, R. Mahley, A. Sajantila, G. Stamatoyannopoulos, J. T. S. Wee, R. Khusainova, E. Khusnutdinova, S. Litvinov, G. Ayodo, D. Comas, M. F. Hammer, T. Kivisild, W. Klitz, C. A. Winkler, D. Labuda, M. Bamshad, L. B. Jorde, S. A. Tishkoff, W. S. Watkins, M. Metspalu, S. Dryomov, R. Sukernik, L. Singh, K. Thangaraj, S. Pääbo, J. Kelso, N. Patterson, D. Reich, the simons genome diversity project: 300 Genomes from 142 diverse populations. *Nature* **538**, 201–206 (2016).

82. V. M. Narasimhan, N. Patterson, P. Moorjani, N. Rohland, R. Bernardos, S. Mallick, I. Lazaridis, N. Nakatsuka, I. Olalde, M. Lipson, A. M. Kim, L. M. Olivieri, A. Coppa, M. Vidale, J. Mallory, V. Moiseyev, E. Kitov, J. Monge, N. Adamski, N. Alex, N. Broomandkhoshbacht, F. Candilio, K. Callan, O. Cheronet, B. J. Culleton, M. Ferry, D. Fernandes, S. Freilich, B. Gamarra, D. Gaudio, M. Hajdinjak, É. Harney, T. K. Harper, D. Keating, A. M. Lawson, M. Mah, K. Mandl, M. Michel, M. Novak, J. Oppenheimer, N. Rai, K. Sirak, V. Slon, K. Stewardson, F. Zalzala, Z. Zhang, G. Akhatov, A. N. Bagashev, A. Bagnera, B. Baitanayev, J. Bendezu-Sarmiento, A. A. Bissembaev, G. L. Bonora, T. T. Chargynov, T. Chikisheva, P. K. Dashkovskiy, A. Derevianko, M. Dobeš, K. Douka, N. Dubova, M. N. Duisengali, D. Enshin, A. Epimakhov, A. V. Fribus, D. Fuller, A. Goryachev, A. Gromov, S. P. Grushin, B. Hanks, M. Judd, E. Kazizov, A. Khokhlov, A. P. Krygin, E.

Kupriyanova, P. Kuznetsov, D. Luiselli, F. Maksudov, A. M. Mamedov, T. B. Mamirov, C. Meiklejohn, D. C. Merrett, R. Micheli, O. Mochalov, S. Mustafokulov, A. Nayak, D. Pettener, R. Potts, D. Razhev, M. Rykun, S. Sarno, T. M. Savenkova, K. Sikhymbaeva, S. M. Slepchenko, O. A. Soltobaev, N. Stepanova, S. Svyatko, K. Tabaldiev, M. Teschler-Nicola, A. A. Tishkin, V. V. Tkachev, S. Vasilyev, P. Velemínský, D. Voyakin, A. Yermolayeva, M. Zahir, V. S. Zubkov, A. Zubova, V. S. Shinde, C. Lalueza-Fox, M. Meyer, D. Anthony, N. Boivin, K. Thangaraj, D. J. Kennett, M. Frachetti, R. Pinhasi, D. Reich, The formation of human populations in South and Central Asia. *Science* **365**, eaat7487 (2019).

83. M. E. Allentoft, M. Sikora, K.-G. Sjögren, S. Rasmussen, M. Rasmussen, J. Stenderup, P. B. Damgaard, H. Schroeder, T. Ahlström, L. Vinner, A.-S. Malaspinas, A. Margaryan, T. Higham, D. Chivall, N. Lynnerup, L. Harvig, J. Baron, P. D. Casa, P. Dąbrowski, P. R. Duffy, A. V. Ebel, A. Epimakhov, K. Frei, M. Furmanek, T. Gralak, A. Gromov, S. Gronkiewicz, G. Grupe, T. Hajdu, R. Jarysz, V. Khartanovich, A. Khokhlov, V. Kiss, J. Kolář, A. Kriiska, I. Lasak, C. Longhi, G. McGlynn, A. Merkevicius, I. Merkyte, M. Metspalu, R. Mkrtchyan, V. Moiseyev, L. Paja, G. Pálfi, D. Pokutta, Ł. Pospieszny, T. D. Price, L. Saag, M. Sablin, N. Shishlina, V. Smrčka, V. I. Soenov, V. Szeverényi, G. Tóth, S. V. Trifanova, L. Varul, M. Vicze, L. Yepiskoposyan, V. Zhitenev, L. Orlando, T. Sicheritz-Pontén, S. Brunak, R. Nielsen, K. Kristiansen, E. Willerslev, Population genomics of Bronze Age Eurasia. *Nature* **522**, 167–172 (2015).

84. C. Jeong, K. Wang, S. Wilkin, W. T. T. Taylor, B. K. Miller, J. H. Bemmann, R. Stahl, C. Chiovelli, F. Knolle, S. Ulziibayar, D. Khatanbaatar, D. Erdenebaatar, U. Erdenebat, A. Ochir, G. Ankhsanaa, C. Vanchigdash, B. Ochir, C. Munkhbayar, D. Tumen, A. Kovalev, N. Kradin, B. A. Bazarov, D. A. Miyagashev, P. B. Konovalov, E. Zhambaltarova, A. V. Miller, W. Haak, S. Schiffels, J. Krause, N. Boivin, M. Erdene, J. Hendy, C. Warinner, A dynamic 6,000-year genetic history of Eurasia's Eastern Steppe. *Cell* **183**, 890–904.e29 (2020).

85. M. Raghavan, P. Skoglund, K. E. Graf, M. Metspalu, A. Albrechtsen, I. Moltke, S. Rasmussen, T. W. Stafford Jr., L. Orlando, E. Metspalu, M. Karmin, K. Tambets, S. Rootsi, R. Mägi, P. F. Campos, E. Balanovska, O. Balanovsky, E. Khusnutdinova, S. Litvinov, L. P. Osipova, S. A. Fedorova, M. I. Voevoda, M. DeGiorgio, T. Sicheritz-Ponten, S. Brunak, S. Demeshchenko, T. Kivisild, R. Villems, R. Nielsen, M. Jakobsson, E. Willerslev, Upper

Palaeolithic Siberian genome reveals dual ancestry of Native Americans. *Nature* **505**, 87–91 (2014).

86. Q. Fu, C. Posth, M. Hajdinjak, M. Petr, S. Mallick, D. Fernandes, A. Furtwängler, W. Haak, M. Meyer, A. Mittnik, B. Nickel, A. Peltzer, N. Rohland, V. Slon, S. Talamo, I. Lazaridis, M. Lipson, I. Mathieson, S. Schiffels, P. Skoglund, A. P. Derevianko, N. Drozdov, V. Slavinsky, A. Tsybankov, R. G. Cremonesi, F. Mallegni, B. Gély, E. Vacca, M. R. G. Morales, L. G. Straus, C. Neugebauer-Maresch, M. Teschler-Nicola, S. Constantin, O. T. Moldovan, S. Benazzi, M. Peresani, D. Coppola, M. Lari, S. Ricci, A. Ronchitelli, F. Valentin, C. Thevenet, K. Wehrberger, D. Grigorescu, H. Rougier, I. Crevecoeur, D. Flas, P. Semal, M. A. Mannino, C. Cupillard, H. Bocherens, N. J. Conard, K. Harvati, V. Moiseyev, D. G. Drucker, J. Svoboda, M. P. Richards, D. Caramelli, R. Pinhasi, J. Kelso, N. Patterson, J. Krause, S. Pääbo, D. Reich, The genetic history of Ice Age Europe. *Nature* **534**, 200–205 (2016).

87. G. A. Gnecchi-Ruscone, E. Khussainova, N. Kahbatkyzy, L. Musralina, M. A. Spyrou, R. A. Bianco, R. Radzeviciute, N. F. G. Martins, C. Freund, O. Iksan, A. Garshin, Z. Zhaniyazov, B. Bekmanov, E. Kitov, Z. Samashev, A. Beisenov, N. Berezina, Y. Berezin, A. Z. Bíró, S. Évinger, A. Bissembaev, G. Akhatov, A. Mamedov, A. Onggaruly, D. Voyakin, A. Chotbayev, Y. Kariyev, A. Buzhilova, L. Djansugurova, C. Jeong, J. Krause, Ancient genomic time transect from the Central Asian Steppe unravels the history of the Scythians. *Sci. Adv.* **7**, eabe4414 (2021).

88. E. Skourtanioti, Y. S. Erdal, M. Frangipane, F. Balossi Restelli, K. A. Yener, F. Pinnock, P. Matthiae, R. Özbal, U.-D. Schoop, F. Guliyev, T. Akhundov, B. Lyonnet, E. L. Hammer, S. E. Nugent, M. Burri, G. U. Neumann, S. Penske, T. Ingman, M. Akar, R. Shafiq, G. Palumbi, S. Eisenmann, M. D'Andrea, A. B. Rohrlach, C. Warinner, C. Jeong, P. W. Stockhammer, W. Haak, J. Krause, Genomic history of neolithic to bronze age Anatolia, Northern Levant, and Southern Caucasus. *Cell* **181**, 1158–1175.e28 (2020).

89. P. d. B. Damgaard, N. Marchi, S. Rasmussen, M. Peyrot, G. Renaud, T. Korneliussen, J. V. Moreno-Mayar, M. W. Pedersen, A. Goldberg, E. Usmanova, N. Baimukhanov, V. Loman, L. Hedeager, A. G. Pedersen, K. Nielsen, G. Afanasiev, K. Akmatov, A. Aldashev, A. Alpaslan, G. Baimbetov, V. I. Bazaliiskii, A. Beisenov, B. Boldbaatar, B. Boldgiv, C. Dorzhu, S.

Ellingvag, D. Erdenebaatar, R. Dajani, E. Dmitriev, V. Evdokimov, K. M. Frei, A. Gromov, A. Goryachev, H. Hakonarson, T. Hegay, Z. Khachatryan, R. Khaskhanov, E. Kitov, A. Kolbina, T. Kubatbek, A. Kukushkin, I. Kukushkin, N. Lau, A. Margaryan, I. Merkyte, I. V. Mertz, V. K. Mertz, E. Mijiddorj, V. Moiyesev, G. Mukhtarova, B. Nurmukhanbetov, Z. Orozbekova, I. Panyushkina, K. Pieta, V. Smrčka, I. Shevnina, A. Logvin, K.-G. Sjögren, T. Štolcová, A. M. Taravella, K. Tashbaeva, A. Tkachev, T. Tulegenov, D. Voyakin, L. Yepiskoposyan, S. Undrakhbold, V. Varfolomeev, A. Weber, M. A. W. Sayres, N. Kradin, M. E. Allentoft, L. Orlando, R. Nielsen, M. Sikora, E. Heyer, K. Kristiansen, E. Willerslev, 137 Ancient human genomes from across the Eurasian steppes. *Nature* **557**, 369–374 (2018).

90. M. Unterländer, F. Palstra, I. Lazaridis, A. Pilipenko, Z. Hofmanová, M. Groß, C. Sell, J. Blöcher, K. Kirsanow, N. Rohland, B. Rieger, E. Kaiser, W. Schier, D. Pozdniakov, A. Khokhlov, M. Georges, S. Wilde, A. Powell, E. Heyer, M. Currat, D. Reich, Z. Samashev, H. Parzinger, V. I. Molodin, J. Burger, Ancestry and demography and descendants of Iron Age nomads of the Eurasian Steppe. *Nat. Commun.* **8**, 14615 (2017).

91. K. Prüfer, F. Racimo, N. Patterson, F. Jay, S. Sankararaman, S. Sawyer, A. Heinze, G. Renaud, P. H. Sudmant, C. de Filippo, H. Li, S. Mallick, M. Dannemann, Q. Fu, M. Kircher, M. Kuhlwilm, M. Lachmann, M. Meyer, M. Ongyerth, M. Siebauer, C. Theunert, A. Tandon, P. Moorjani, J. Pickrell, J. C. Mullikin, S. H. Vohr, R. E. Green, I. Hellmann, P. L. F. Johnson, H. Blanche, H. Cann, J. O. Kitzman, J. Shendure, E. E. Eichler, E. S. Lein, T. E. Bakken, L. V. Golovanova, V. B. Doronichev, M. V. Shunkov, A. P. Derevianko, B. Viola, M. Slatkin, D. Reich, J. Kelso, S. Pääbo, The complete genome sequence of a Neanderthal from the Altai Mountains. *Nature* **505**, 43–49 (2014).

92. K. Wang, H. Yu, R. Radzevičiūtė, Y. F. Kiryushin, A. A. Tishkin, Y. V. Frolov, N. F. Stepanova, K. Y. Kiryushin, A. L. Kungurov, S. V. Shnaider, S. S. Tur, M. P. Tiunov, A. V. Zubova, M. Pevzner, T. Karimov, A. Buzhilova, V. Slon, C. Jeong, J. Krause, C. Posth, Middle Holocene Siberian genomes reveal highly connected gene pools throughout North Asia. *Curr. Biol.* **33**, 423–433.e5 (2023).

93. G. M. Kılınç, N. Kashuba, D. Koptekin, N. Bergfeldt, H. M. Dönertaş, R. Rodríguez-Varela, D. Shergin, G. Ivanov, D. Kichigin, K. Pestereva, D. Volkov, P. Mandryka, A. Kharinskii, A.

Tishkin, E. Ineshin, E. Kovychev, A. Stepanov, L. Dalén, T. Günther, E. Kırdök, M. Jakobsson, M. Somel, M. Krzewińska, J. Storå, A. Götherström, Human population dynamics and Yersinia pestis in ancient northeast Asia. *Sci. Adv.* **7**, eabc4587 (2021).

94. I. Lazaridis, A. Mittnik, N. Patterson, S. Mallick, N. Rohland, S. Pfrengle, A. Furtwängler, A. Peltzer, C. Posth, A. Vasilakis, P. J. P. McGeorge, E. Konsolaki-Yannopoulou, G. Korres, H. Martlew, M. Michalodimitrakis, M. Özsait, N. Özsait, A. Papathanasiou, M. Richards, S. A. Roodenberg, Y. Tzedakis, R. Arnott, D. M. Fernandes, J. R. Hughey, D. M. Lotakis, P. A. Navas, Y. Maniatis, J. A. Stamatoyannopoulos, K. Stewardson, P. Stockhammer, R. Pinhasi, D. Reich, J. Krause, G. Stamatoyannopoulos, Genetic origins of the Minoans and Mycenaeans. *Nature* **548**, 214–218 (2017).

95. P. de Barros Damgaard, R. Martiniano, J. Kamm, J. V. Moreno-Mayar, G. Kroonen, M. Peyrot, G. Barjamovic, S. Rasmussen, C. Zacho, N. Baimukhanov, V. Zaibert, V. Merz, A. Biddanda, I. Merz, V. Loman, V. Evdokimov, E. Usmanova, B. Hemphill, A. Seguin-Orlando, F. E. Yediay, I. Ullah, K.-G. Sjögren, K. H. Iversen, J. Choin, C. de la Fuente, M. Ilardo, H. Schroeder, V. Moiseyev, A. Gromov, A. Polyakov, S. Omura, S. Y. Senyurt, H. Ahmad, C. McKenzie, A. Margaryan, A. Hameed, A. Samad, N. Gul, M. H. Khokhar, O. I. Goriunova, V. I. Bazaliiskii, J. Novembre, A. W. Weber, L. Orlando, M. E. Allentoft, R. Nielsen, K. Kristiansen, M. Sikora, A. K. Outram, R. Durbin, E. Willerslev, The first horse herders and the impact of early Bronze Age steppe expansions into Asia. *Science* **360**, eaar7711 (2018).

96. Z. Hofmanová, S. Kreutzer, G. Hellenthal, C. Sell, Y. Diekmann, D. Díez-del-Molino, L. van Dorp, S. López, A. Kousathanas, V. Link, K. Kirsanow, L. M. Cassidy, R. Martiniano, M. Strobel, A. Scheu, K. Kotsakis, P. Halstead, S. Triantaphyllou, N. Kyparissi-Apostolika, D. Urem-Kotsou, C. Ziota, F. Adaktylou, S. Gopalan, D. M. Bobo, L. Winkelbach, J. Blöcher, M. Unterländer, C. Leuenberger, Ç. Çilingiroğlu, B. Horejs, F. Gerritsen, S. J. Shennan, D. G. Bradley, M. Currat, K. R. Veeramah, D. Wegmann, M. G. Thomas, C. Papageorgopoulou, J. Burger, Early farmers from across Europe directly descended from Neolithic Aegeans. *Proc. Natl. Acad. Sci. U.S.A.* **113**, 6886–6891 (2016).

97. C. Ning, T. Li, K. Wang, F. Zhang, T. Li, X. Wu, S. Gao, Q. Zhang, H. Zhang, M. J. Hudson, G. Dong, S. Wu, Y. Fang, C. Liu, C. Feng, W. Li, T. Han, R. Li, J. Wei, Y. Zhu, Y. Zhou,

C.-C. Wang, S. Fan, Z. Xiong, Z. Sun, M. Ye, L. Sun, X. Wu, F. Liang, Y. Cao, X. Wei, H. Zhu, H. Zhou, J. Krause, M. Robbeets, C. Jeong, Y. Cui, Ancient genomes from northern China suggest links between subsistence changes and human migration. *Nat. Commun.* **11**, 2700 (2020).

98. X. Mao, H. Zhang, S. Qiao, Y. Liu, F. Chang, P. Xie, M. Zhang, T. Wang, M. Li, P. Cao, R. Yang, F. Liu, Q. Dai, X. Feng, W. Ping, C. Lei, J. W. Olsen, E. A. Bennett, Q. Fu, The deep population history of northern East Asia from the Late Pleistocene to the Holocene. *Cell* **184**, 3256–3266.e13 (2021).

99. M. Feldman, D. M. Master, R. A. Bianco, M. Burri, P. W. Stockhammer, A. Mittnik, A. J. Aja, C. Jeong, J. Krause, Ancient DNA sheds light on the genetic origins of early Iron Age Philistines. *Sci. Adv.* **5**, eaax0061 (2019).

100. T. Wang, W. Wang, G. Xie, Z. Li, X. Fan, Q. Yang, X. Wu, P. Cao, Y. Liu, R. Yang, F. Liu, Q. Dai, X. Feng, X. Wu, L. Qin, F. Li, W. Ping, L. Zhang, M. Zhang, Y. Liu, X. Chen, D. Zhang, Z. Zhou, Y. Wu, H. Shafiey, X. Gao, D. Curnoe, X. Mao, E. A. Bennett, X. Ji, M. A. Yang, Q. Fu, Human population history at the crossroads of East and Southeast Asia since 11,000 years ago. *Cell* **184**, 3829–3841.e21 (2021).

101. H. Yu, M. A. Spyrou, M. Karapetian, S. Shnaider, R. Radzevičiūtė, K. Nägele, G. U. Neumann, S. Penske, J. Zech, M. Lucas, P. LeRoux, P. Roberts, G. Pavlenok, A. Buzhilova, C. Posth, C. Jeong, J. Krause, Paleolithic to bronze age siberians reveal connections with first americans and across Eurasia. *Cell* **181**, 1232–1245.e20 (2020).

102. I. Mathieson, S. Alpaslan-Roodenberg, C. Posth, A. Szécsényi-Nagy, N. Rohland, S. Mallick, I. Olalde, N. Broomandkhoshbacht, F. Candilio, O. Cheronet, D. Fernandes, M. Ferry, B. Gamarra, G. G. Fortes, W. Haak, E. Harney, E. Jones, D. Keating, B. Krause-Kyora, I. Kucukkalipci, M. Michel, A. Mittnik, K. Nägele, M. Novak, J. Oppenheimer, N. Patterson, S. Pfrengle, K. Sirak, K. Stewardson, S. Vai, S. Alexandrov, K. W. Alt, R. Andreescu, D. Antonović, A. Ash, N. Atanassova, K. Bacvarov, M. B. Gusztáv, H. Bocherens, M. Bolus, A. Boroneanţ, Y. Boyadzhiev, A. Budnik, J. Burmaz, S. Chohadzhiev, N. J. Conard, R. Cottiaux, M. Čuka, C. Cupillard, D. G. Drucker, N. Elenski, M. Francken, B. Galabova, G. Ganetsovski, B. Gély, T. Hajdu, V. Handzhyiska, K. Harvati, T. Higham, S.

Iliev, I. Janković, I. Karavanić, D. J. Kennett, D. Komšo, A. Kozak, D. Labuda, M. Lari, C. Lazar, M. Leppek, K. Leshtakov, D. L. Vetro, D. Los, I. Lozanov, M. Malina, F. Martini, K. McSweeney, H. Meller, M. Menđušić, P. Mirea, V. Moiseyev, V. Petrova, T. D. Price, A. Simalcsik, L. Sineo, M. Šlaus, V. Slavchev, P. Stanev, A. Starović, T. Szeniczey, S. Talamo, M. Teschler-Nicola, C. Thevenet, I. Valchev, F. Valentin, S. Vasilyev, F. Veljanovska, S. Venelinova, E. Veselovskaya, B. Viola, C. Virag, J. Zaninović, S. Zäuner, P. W. Stockhammer, G. Catalano, R. Krauß, D. Caramelli, G. Zariņa, B. Gaydarska, M. Lillie, A. G. Nikitin, I. Potekhina, A. Papathanasiou, D. Borić, C. Bonsall, J. Krause, R. Pinhasi, D. Reich, The genomic history of southeastern Europe. *Nature* **555**, 197–203 (2018).

103. M. Lipson, O. Cheronet, S. Mallick, N. Rohland, M. Oxenham, M. Pietrusewsky, T. O. Pryce, A. Willis, H. Matsumura, H. Buckley, K. Domett, G. H. Nguyen, H. H. Trinh, A. A. Kyaw, T. T. Win, B. Pradier, N. Broomandkhoshbacht, F. Candilio, P. Changmai, D. Fernandes, M. Ferry, B. Gamarra, E. Harney, J. Kampuansai, W. Kutanan, M. Michel, M. Novak, J. Oppenheimer, K. Sirak, K. Stewardson, Z. Zhang, P. Flegontov, R. Pinhasi, D. Reich, Ancient genomes document multiple waves of migration in Southeast Asian prehistory. *Science* **361**, 92–95 (2018).

104. E. R. Jones, G. Gonzalez-Fortes, S. Connell, V. Siska, A. Eriksson, R. Martiniano, R. L. McLaughlin, M. Gallego Llorente, L. M. Cassidy, C. Gamba, T. Meshveliani, O. Bar-Yosef, W. Müller, A. Belfer-Cohen, Z. Matskevich, N. Jakeli, T. F. G. Higham, M. Currat, D. Lordkipanidze, M. Hofreiter, A. Manica, R. Pinhasi, D. G. Bradley, Upper Palaeolithic genomes reveal deep roots of modern Eurasians. *Nat. Commun.* **6**, 8912 (2015).

105. C.-C. Wang, H.-Y. Yeh, A. N. Popov, H.-Q. Zhang, H. Matsumura, K. Sirak, O. Cheronet, A. Kovalev, N. Rohland, A. M. Kim, S. Mallick, R. Bernardos, D. Tumen, J. Zhao, Y.-C. Liu, J.-Y. Liu, M. Mah, K. Wang, Z. Zhang, N. Adamski, N. Broomandkhoshbacht, K. Callan, F. Candilio, K. S. D. Carlson, B. J. Culleton, L. Eccles, S. Freilich, D. Keating, A. M. Lawson, K. Mandl, M. Michel, J. Oppenheimer, K. T. Özdoğan, K. Stewardson, S. Wen, S. Yan, F. Zalzala, R. Chuang, C.-J. Huang, H. Looh, C.-C. Shiung, Y. G. Nikitin, A. V. Tabarev, A. A. Tishkin, S. Lin, Z.-Y. Sun, X.-M. Wu, T.-L. Yang, X. Hu, L. Chen, H. Du, J. Bayarsaikhan, E. Mijiddorj, D. Erdenebaatar, T.-O. Iderkhangai, E. Myagmar, H. Kanzawa-Kiriyama, M. Nishino, K.-i. Shinoda, O. A. Shubina, J. Guo, W. Cai, Q. Deng, L. Kang, D. Li, D. Li, R. Lin,

Nini, R. Shrestha, L.-X. Wang, L. Wei, G. Xie, H. Yao, M. Zhang, G. He, X. Yang, R. Hu, M. Robbeets, S. Schiffels, D. J. Kennett, L. Jin, H. Li, J. Krause, R. Pinhasi, D. Reich, Genomic insights into the formation of human populations in East Asia. *Nature* **591**, 413–419 (2021).

106. G. M. Kılınç, A. Omrak, F. Özer, T. Günther, A. M. Büyükkarakaya, E. Bıçakçı, D. Baird, H. M. Dönertaş, A. Ghalichi, R. Yaka, D. Koptekin, S. C. Açan, P. Parvizi, M. Krzewińska, E. A. Daskalaki, E. Yüncü, N. D. Dağtaş, A. Fairbairn, J. Pearson, G. Mustafaoğlu, Y. S. Erdal, Y. G. Çakan, İ. Togan, M. Somel, J. Storå, M. Jakobsson, A. Götherström, The demographic development of the first farmers in anatolia. *Curr. Biol.* **26**, 2659–2666 (2016).

107. M. Haber, C. Doumet-Serhal, C. Scheib, Y. Xue, P. Danecek, M. Mezzavilla, S. Youhanna, R. Martiniano, J. Prado-Martinez, M. Szpak, E. Matisoo-Smith, H. Schutkowski, R. Mikulski, P. Zalloua, T. Kivisild, C. Tyler-Smith, Continuity and admixture in the last five millennia of levantine history from ancient canaanite and present-day lebanese genome sequences. *Am. J. Human Genet.* **101**, 274–282 (2017).

108. C.-C. Wang, S. Reinhold, A. Kalmykov, A. Wissgott, G. Brandt, C. Jeong, O. Cheronet, M. Ferry, E. Harney, D. Keating, S. Mallick, N. Rohland, K. Stewardson, A. R. Kantorovich, V. E. Maslov, V. G. Petrenko, V. R. Erlikh, B. C. Atabiev, R. G. Magomedov, P. L. Kohl, K. W. Alt, S. L. Pichler, C. Gerling, H. Meller, B. Vardanyan, L. Yeganyan, A. D. Rezepkin, D. Mariaschk, N. Berezina, J. Gresky, K. Fuchs, C. Knipper, S. Schiffels, E. Balanovska, O. Balanovsky, I. Mathieson, T. Higham, Y. B. Berezin, A. Buzhilova, V. Trifonov, R. Pinhasi, A. B. Belinskij, D. Reich, S. Hansen, J. Krause, W. Haak, Ancient human genome-wide data from a 3000-year interval in the Caucasus corresponds with eco-geographic regions. *Nat. Commun.* **10**, 590 (2019).

109. C. Jeong, A. T. Ozga, D. B. Witonsky, H. Malmström, H. Edlund, C. A. Hofman, R. W. Hagan, M. Jakobsson, C. M. Lewis, M. S. Aldenderfer, A. Di Rienzo, C. Warinner, Long-term genetic stability and a high-altitude East Asian origin for the peoples of the high valleys of the Himalayan arc. *Proc. Natl. Acad. Sci. U.S.A.* **113**, 7485–7490 (2016).

110. M. A. Yang, X. Fan, B. Sun, C. Chen, J. Lang, Y.-C. Ko, C.-h. Tsang, H. Chiu, T. Wang, Q. Bao, X. Wu, M. Hajdinjak, A. M.-S. Ko, M. Ding, P. Cao, R. Yang, F. Liu, B. Nickel, Q. Dai, X. Feng, L. Zhang, C. Sun, C. Ning, W. Zeng, Y. Zhao, M. Zhang, X. Gao, Y. Cui, D.

Reich, M. Stoneking, Q. Fu, Ancient DNA indicates human population shifts and admixture in northern and southern China. *Science* **369**, 282–288 (2020).

111. M. Rasmussen, S. L. Anzick, M. R. Waters, P. Skoglund, M. DeGiorgio, T. W. Stafford, S. Rasmussen, I. Moltke, A. Albrechtsen, S. M. Doyle, G. D. Poznik, V. Gudmundsdottir, R. Yadav, A.-S. Malaspinas, S. S. White V, M. E. Allentoft, O. E. Cornejo, K. Tambets, A. Eriksson, P. D. Heintzman, M. Karmin, T. S. Korneliussen, D. J. Meltzer, T. L. Pierre, J. Stenderup, L. Saag, V. M. Warmuth, M. C. Lopes, R. S. Malhi, S. Brunak, T. Sicheritz-Ponten, I. Barnes, M. Collins, L. Orlando, F. Balloux, A. Manica, R. Gupta, M. Metspalu, C. D. Bustamante, M. Jakobsson, R. Nielsen, E. Willerslev, The genome of a Late Pleistocene human from a Clovis burial site in western Montana. *Nature* **506**, 225–229 (2014).

112. C. E. G. Amorim, S. Vai, C. Posth, A. Modi, I. Koncz, S. Hakenbeck, M. C. La Rocca, B. Mende, D. Bobo, W. Pohl, L. P. Baricco, E. Bedini, P. Francalacci, C. Giostra, T. Vida, D. Winger, U. von Freeden, S. Ghirotto, M. Lari, G. Barbujani, J. Krause, D. Caramelli, P. J. Geary, K. R. Veeramah, Understanding 6th-century barbarian social organization and migration through paleogenomics. *Nat. Commun.* **9**, 3547 (2018).

113. L. Saag, L. Varul, C. L. Scheib, J. Stenderup, M. E. Allentoft, L. Saag, L. Pagani, M. Reidla, K. Tambets, E. Metspalu, A. Kriiska, E. Willerslev, T. Kivisild, M. Metspalu, Extensive farming in estonia started through a sex-biased migration from the steppe. *Curr. Biol.* **27**, 2185–2193.e6 (2017).

114. M. Meyer, M. Kircher, M.-T. Gansauge, H. Li, F. Racimo, S. Mallick, J. G. Schraiber, F. Jay, K. Prüfer, C. de Filippo, P. H. Sudmant, C. Alkan, Q. Fu, R. Do, N. Rohland, A. Tandon, M. Siebauer, R. E. Green, K. Bryc, A. W. Briggs, U. Stenzel, J. Dabney, J. Shendure, J. Kitzman, M. F. Hammer, M. V. Shunkov, A. P. Derevianko, N. Patterson, A. M. Andrés, E. E. Eichler, M. Slatkin, D. Reich, J. Kelso, S. Pääbo, A high-coverage genome sequence from an archaic denisovan individual. *Science* **338**, 222–226 (2012).

115. M. Sikora, V. V. Pitulko, V. C. Sousa, M. E. Allentoft, L. Vinner, S. Rasmussen, A. Margaryan, P. de Barros Damgaard, C. de la Fuente, G. Renaud, M. A. Yang, Q. Fu, I. Dupanloup, K. Giampoudakis, D. Nogués-Bravo, C. Rahbek, G. Kroonen, M. Peyrot, H. McColl, S. V. Vasilyev, E. Veselovskaya, M. Gerasimova, E. Y. Pavlova, V. G. Chasnyk, P.

A. Nikolskiy, A. V. Gromov, V. I. Khartanovich, V. Moiseyev, P. S. Grebenyuk, A. Y. Fedorchenko, A. I. Lebedintsev, S. B. Slobodin, B. A. Malyarchuk, R. Martiniano, M. Meldgaard, L. Arppe, J. U. Palo, T. Sundell, K. Mannermaa, M. Putkonen, V. Alexandersen, C. Primeau, N. Baimukhanov, R. S. Malhi, K.-G. Sjögren, K. Kristiansen, A. Wessman, A. Sajantila, M. M. Lahr, R. Durbin, R. Nielsen, D. J. Meltzer, L. Excoffier, E. Willerslev, The population history of northeastern Siberia since the Pleistocene. *Nature* **570**, 182–188 (2019).

116. V. Siska, E. R. Jones, S. Jeon, Y. Bhak, H.-M. Kim, Y. S. Cho, H. Kim, K. Lee, E. Veselovskaya, T. Balueva, M. Gallego-Llorente, M. Hofreiter, D. G. Bradley, A. Eriksson, R. Pinhasi, J. Bhak, A. Manica, Genome-wide data from two early Neolithic East Asian individuals dating to 7700 years ago. *Sci. Adv.* **3**, e1601877 (2017).

117. F. Zhang, C. Ning, A. Scott, Q. Fu, R. Bjørn, W. Li, D. Wei, W. Wang, L. Fan, I. Abuduresule, X. Hu, Q. Ruan, A. Niyazi, G. Dong, P. Cao, F. Liu, Q. Dai, X. Feng, R. Yang, Z. Tang, P. Ma, C. Li, S. Gao, Y. Xu, S. Wu, S. Wen, H. Zhu, H. Zhou, M. Robbeets, V. Kumar, J. Krause, C. Warinner, C. Jeong, Y. Cui, The genomic origins of the Bronze Age Tarim Basin mummies. *Nature* **599**, 256–261 (2021).

118. A. Mittnik, C.-C. Wang, S. Pfrengle, M. Daubaras, G. Zariņa, F. Hallgren, R. Allmäe, V. Khartanovich, V. Moiseyev, M. Tõrv, A. Furtwängler, A. Andrades Valtueña, M. Feldman, C. Economou, M. Oinonen, A. Vasks, E. Balanovska, D. Reich, R. Jankauskas, W. Haak, S. Schiffels, J. Krause, The genetic prehistory of the Baltic Sea region. *Nat. Commun.* **9**, 442 (2018).

119. M. G. Llorente, E. R. Jones, A. Eriksson, V. Siska, K. W. Arthur, J. W. Arthur, M. C. Curtis, J. T. Stock, M. Coltorti, P. Pieruccini, S. Stretton, F. Brock, T. Higham, Y. Park, M. Hofreiter, D. G. Bradley, J. Bhak, R. Pinhasi, A. Manica, Ancient Ethiopian genome reveals extensive Eurasian admixture in Eastern Africa. *Science* **350**, 820–822 (2015).

120. H. Kanzawa-Kiriyama, T. A. Jinam, Y. Kawai, T. Sato, K. Hosomichi, A. Tajima, N. Adachi, H. Matsumura, K. Kryukov, N. Saitou, K.-I. Shinoda, Late Jomon male and female genome sequences from the Funadomari site in Hokkaido, Japan. *Anthropol. Sci.* **127**, 83–108 (2019).

121. J. Li, Y. Zhang, Y. Zhao, Y. Chen, A. Ochir, H. Sarenbilige, H. Z. Zhu, The genome of an ancient Rouran individual reveals an important paternal lineage in the Donghu population. *Am. J. Phys. Anthropol.* **166**, 895–905 (2018).

122. M. Krzewińska, G. M. Kılınç, A. Juras, D. Koptekin, M. Chyleński, A. G. Nikitin, N. Shcherbakov, I. Shuteleva, T. Leonova, L. Kraeva, F. A. Sungatov, A. N. Sultanova, I. Potekhina, S. Łukasik, M. Krenz-Niedbała, L. Dalén, V. Sinika, M. Jakobsson, J. Storå, A. Götherström, Ancient genomes suggest the eastern Pontic-Caspian steppe as the source of western Iron Age nomads. *Sci. Adv.* **4**, eaat4457 (2018).

123. G. A. Gnecchi-Ruscone, A. Szécsényi-Nagy, I. Koncz, G. Csiky, Z. Rácz, A. B. Rohrlach, G. Brandt, N. Rohland, V. Csáky, O. Cheronet, B. Szeifert, T. Á. Rácz, A. Benedek, Z. Bernert, N. Berta, S. Czifra, J. Dani, Z. Farkas, T. Hága, T. Hajdu, M. Jászberényi, V. Kisjuhász, B. Kolozsi, P. Major, A. Marcsik, B. N. Kovacsóczy, C. Balogh, G. M. Lezsák, J. G. Ódor, M. Szelekovszky, T. Szeniczey, J. Tárnoki, Z. Tóth, E. K. Tutkovics, B. G. Mende, P. Geary, W. Pohl, T. Vida, R. Pinhasi, D. Reich, Z. Hofmanová, C. Jeong, J. Krause, Ancient genomes reveal origin and rapid trans-Eurasian migration of 7th century Avar elites. *Cell* **185**, 1402–1413.e21 (2022).

124. H. McColl, F. Racimo, L. Vinner, F. Demeter, T. Gakuhari, J. V. Moreno-Mayar, G. van Driem, U. Gram Wilken, A. Seguin-Orlando, C. de la Fuente Castro, S. Wasef, R. Shoocongdej, V. Souksavatdy, T. Sayavongkhamdy, M. M. Saidin, M. E. Allentoft, T. Sato, A.-S. Malaspinas, F. A. Aghakhanian, T. Korneliussen, A. Prohaska, A. Margaryan, P. de Barros Damgaard, S. Kaewsutthi, P. Lertrit, T. M. H. Nguyen, H.-c. Hung, T. Minh Tran, H. Nghia Truong, G. H. Nguyen, S. Shahidan, K. Wiradnyana, H. Matsumae, N. Shigehara, M. Yoneda, H. Ishida, T. Masuyama, Y. Yamada, A. Tajima, H. Shibata, A. Toyoda, T. Hanihara, S. Nakagome, T. Deviese, A.-M. Bacon, P. Duringer, J.-L. Ponche, L. Shackelford, E. Patole-Edoumba, A. T. Nguyen, B. Bellina-Pryce, J.-C. Galipaud, R. Kinaston, H. Buckley, C. Pottier, S. Rasmussen, T. Higham, R. A. Foley, M. M. Lahr, L. Orlando, M. Sikora, M. E. Phipps, H. Oota, C. Higham, D. M. Lambert, E. Willerslev, The prehistoric peopling of Southeast Asia. *Science* **361**, 88–92 (2018).

125.. Harney, H. May, D. Shalem, N. Rohland, S. Mallick, I. Lazaridis, R. Sarig, K. Stewardson, S. Nordenfelt, N. Patterson, I. Hershkovitz, D. Reich, Ancient DNA from Chalcolithic Israel reveals the role of population mixture in cultural transformation. *Nat. Commun.* **9**, 3336 (2018).

126. N. P. Cooke, V. Mattiangeli, L. M. Cassidy, K. Okazaki, C. A. Stokes, S. Onbe, S. Hatakeyama, K. Machida, K. Kasai, N. Tomioka, A. Matsumoto, M. Ito, Y. Kojima, D. G. Bradley, T. Gakuhari, S. Nakagome, Ancient genomics reveals tripartite origins of Japanese populations. *Sci. Adv.* **7**, eabh2419 (2021).

127. M. Rasmussen, M. Sikora, A. Albrechtsen, T. S. Korneliussen, J. V. Moreno-Mayar, G. D. Poznik, C. P. E. Zollikofer, M. S. Ponce de León, M. E. Allentoft, I. Moltke, H. Jónsson, C. Valdiosera, R. S. Malhi, L. Orlando, C. D. Bustamante, T. W. Stafford, D. J. Meltzer, R. Nielsen, E. Willerslev, The ancestry and affiliations of Kennewick Man. *Nature* **523**, 455–458 (2015).

128. P. Skoglund, J. C. Thompson, M. E. Prendergast, A. Mittnik, K. Sirak, M. Hajdinjak, T. Salie, N. Rohland, S. Mallick, A. Peltzer, A. Heinze, I. Olalde, M. Ferry, E. Harney, M. Michel, K. Stewardson, J. I. Cerezo-Román, C. Chiumia, A. Crowther, E. Gomani-Chindebvu, A. O. Gidna, K. M. Grillo, I. T. Helenius, G. Hellenthal, R. Helm, M. Horton, S. López, A. Z. P. Mabulla, J. Parkington, C. Shipton, M. G. Thomas, R. Tibesasa, M. Welling, V. M. Hayes, D. J. Kennett, R. Ramesar, M. Meyer, S. Pääbo, N. Patterson, A. G. Morris, N. Boivin, R. Pinhasi, J. Krause, D. Reich, Reconstructing prehistoric african population structure. *Cell* **171**, 59–71.e21 (2017).

129. C. Jeong, S. Wilkin, T. Amgalantugs, A. S. Bouwman, W. T. T. Taylor, R. W. Hagan, S. Bromage, S. Tsolmon, C. Trachsel, J. Grossmann, J. Littleton, C. A. Makarewicz, J. Krigbaum, M. Burri, A. Scott, G. Davaasambuu, J. Wright, F. Irmer, E. Myagmar, N. Boivin, M. Robbeets, F. J. Rühli, J. Krause, B. Frohlich, J. Hendy, C. Warinner, Bronze Age population dynamics and the rise of dairy pastoralism on the eastern Eurasian steppe. *Proc. Natl. Acad. Sci. U.S.A.* **115**, E11248–E11255 (2018).

130. M. Raghavan, M. DeGiorgio, A. Albrechtsen, I. Moltke, P. Skoglund, T. S. Korneliussen, B. Grønnow, M. Appelt, H. C. Gulløv, T. M. Friesen, W. Fitzhugh, H. Malmström, S.

Rasmussen, J. Olsen, L. Melchior, B. T. Fuller, S. M. Fahrni, T. Stafford, V. Grimes, M. A. P. Renouf, J. Cybulski, N. Lynnerup, M. M. Lahr, K. Britton, R. Knecht, J. Arneborg, M. Metspalu, O. E. Cornejo, A.-S. Malaspinas, Y. Wang, M. Rasmussen, V. Raghavan, T. V. O. Hansen, E. Khusnutdinova, T. Pierre, K. Dneprovsky, C. Andreasen, H. Lange, M. G. Hayes, J. Coltrain, V. A. Spitsyn, A. Götherström, L. Orlando, T. Kivisild, R. Villems, M. H. Crawford, F. C. Nielsen, J. Dissing, J. Heinemeier, M. Meldgaard, C. Bustamante, D. H. O'Rourke, M. Jakobsson, M. T. P. Gilbert, R. Nielsen, E. Willerslev, The genetic prehistory of the New World Arctic. *Science* **345**, 1255832 (2014).

131. M. Lipson, E. A. Sawchuk, J. C. Thompson, J. Oppenheimer, C. A. Tryon, K. L. Ranhorn, K. M. de Luna, K. A. Sirak, I. Olalde, S. H. Ambrose, J. W. Arthur, K. J. W. Arthur, G. Ayodo, A. Bertacchi, J. I. Cerezo-Román, B. J. Culleton, M. C. Curtis, J. Davis, A. O. Gidna, A. Hanson, P. Kaliba, M. Katongo, A. Kwekason, M. F. Laird, J. Lewis, A. Z. P. Mabulla, F. Mapemba, A. Morris, G. Mudenda, R. Mwafulirwa, D. Mwangomba, E. Ndiema, C. Ogola, F. Schilt, P. R. Willoughby, D. K. Wright, A. Zipkin, R. Pinhasi, D. J. Kennett, F. K. Manthi, N. Rohland, N. Patterson, D. Reich, M. E. Prendergast, Ancient DNA and deep population structure in sub-Saharan African foragers. *Nature* **603**, 290–296 (2022).

132. P. Skoglund, S. Mallick, M. C. Bortolini, N. Chennagiri, T. Hünemeier, M. L. Petzl-Erler, F. M. Salzano, N. Patterson, D. Reich, Genetic evidence for two founding populations of the Americas. *Nature* **525**, 104–108 (2015).

133. P. Skoglund, H. Malmström, A. Omrak, M. Raghavan, C. Valdiosera, T. Günther, P. Hall, K. Tambets, J. Parik, K.-G. Sjögren, J. Apel, E. Willerslev, J. Storå, A. Götherström, M. Jakobsson, Genomic diversity and admixture differs for stone-age scandinavian foragers and farmers. *Science* **344**, 747–750 (2014).

134. T. C. Lamnidis, K. Majander, C. Jeong, E. Salmela, A. Wessman, V. Moiseyev, V. Khartanovich, O. Balanovsky, M. Ongyerth, A. Weihmann, A. Sajantila, J. Kelso, S. Pääbo, P. Onkamo, W. Haak, J. Krause, S. Schiffels, Ancient Fennoscandian genomes reveal origin and spread of Siberian ancestry in Europe. *Nat. Commun.* **9**, 5018 (2018).

135. M. Rasmussen, Y. Li, S. Lindgreen, J. S. Pedersen, A. Albrechtsen, I. Moltke, M. Metspalu, E. Metspalu, T. Kivisild, R. Gupta, M. Bertalan, K. Nielsen, M. T. P. Gilbert, Y. Wang, M. Raghavan, P. F. Campos, H. M. Kamp, A. S. Wilson, A. Gledhill, S. Tridico, M. Bunce, E. D. Lorenzen, J. Binladen, X. Guo, J. Zhao, X. Zhang, H. Zhang, Z. Li, M. Chen, L. Orlando, K. Kristiansen, M. Bak, N. Tommerup, C. Bendixen, T. L. Pierre, B. Grønnow, M. Meldgaard, C. Andreasen, S. A. Fedorova, L. P. Osipova, T. F. G. Higham, C. B. Ramsey, T. v. O. Hansen, F. C. Nielsen, M. H. Crawford, S. Brunak, T. Sicheritz-Pontén, R. Villems, R. Nielsen, A. Krogh, J. Wang, E. Willerslev, Ancient human genome sequence of an extinct Palaeo-Eskimo. *Nature* **463**, 757–762 (2010).

136. K.-I. Shinoda, H. Kanzawa-Kiriyama, T. Kakuda, N. Adachi, Genetic characteristics of Yayoi people in Northwestern Kyushu—Ancient genome analysis of human bones excavated from Shimomotoyama Rock Shelter, Sasebo, Nagasaki Prefecture, Japan. *Anthropol. Sci.* **127**, 25–43 (2019).

137. M. Sikora, A. Seguin-Orlando, V. C. Sousa, A. Albrechtsen, T. Korneliussen, A. Ko, S. Rasmussen, I. Dupanloup, P. R. Nigst, M. D. Bosch, G. Renaud, M. E. Allentoft, A. Margaryan, S. V. Vasilyev, E. V. Veselovskaya, S. B. Borutskaya, T. Deviese, D. Comeskey, T. Higham, A. Manica, R. Foley, D. J. Meltzer, R. Nielsen, L. Excoffier, M. Mirazon Lahr, L. Orlando, E. Willerslev, Ancient genomes show social and reproductive behavior of early Upper Paleolithic foragers. *Science* **358**, 659–662 (2017).

138. M. A. Yang, X. Gao, C. Theunert, H. Tong, A. Aximu-Petri, B. Nickel, M. Slatkin, M. Meyer, S. Pääbo, J. Kelso, Q. Fu, 40,000-Year-old individual from asia provides insight into early population structure in Eurasia. *Curr. Biol.* **27**, 3202–3208.e9 (2017).

139. Q. Fu, H. Li, P. Moorjani, F. Jay, S. M. Slepchenko, A. A. Bondarev, P. L. F. Johnson, A. Aximu-Petri, K. Prüfer, C. de Filippo, M. Meyer, N. Zwyns, D. C. Salazar-García, Y. V. Kuzmin, S. G. Keates, P. A. Kosintsev, D. I. Razhev, M. P. Richards, N. V. Peristov, M. Lachmann, K. Douka, T. F. G. Higham, M. Slatkin, J.-J. Hublin, D. Reich, J. Kelso, T. B. Viola, S. Pääbo, Genome sequence of a 45,000-year-old modern human from western Siberia. *Nature* **514**, 445–449 (2014).

140. K. Prüfer, C. de Filippo, S. Grote, F. Mafessoni, P. Korlević, M. Hajdinjak, B. Vernot, L. Skov, P. Hsieh, S. Peyrégne, D. Reher, C. Hopfe, S. Nagel, T. Maricic, Q. Fu, C. Theunert, R. Rogers, P. Skoglund, M. Chintalapati, M. Dannemann, B. J. Nelson, F. M. Key, P. Rudan, Ž. Kućan, I. Gušić, L. V. Golovanova, V. B. Doronichev, N. Patterson, D. Reich, E. E. Eichler, M. Slatkin, M. H. Schierup, A. M. Andrés, J. Kelso, M. Meyer, S. Pääbo, A high-coverage Neandertal genome from Vindija Cave in Croatia. *Science* **358**, 655–658 (2017).
